# Large Language Models in Randomized Controlled Trials Design: Observational Study

**DOI:** 10.2196/67469

**Published:** 2025-09-03

**Authors:** Liyuan Jin, Jasmine Chiat Ling Ong, Kabilan Elangovan, Yuhe Ke, Alexandra Pyle, Daniel Shu Wei Ting, Nan Liu

**Affiliations:** 1 Duke-NUS Medical School, 8 College Road, Singapore, 169857, Singapore, 65 66016503; 2 Division of Pharmacy, Singapore General Hospital, Singapore, Singapore; 3 Artificial Intelligence Office, SingHealth, Singapore, Singapore; 4 Department of Anaesthesiology and Perioperative Medicine, Singapore General Hospital, Singapore, Singapore; 5 Singapore National Eye Centre, Singapore, Singapore; 6 NUS Artificial Intelligence Institute, National University of Singapore, Singapore, Singapore

**Keywords:** GPT-4, LLM-generated clinical trial designs, clinical trial design evaluation, recruitment diversity, eligibility criteria, clinical research ethics, trial failure reduction

## Abstract

**Background:**

Randomized controlled trials (RCTs) face challenges such as limited generalizability, insufficient recruitment diversity, and high failure rates, often due to restrictive eligibility criteria and inefficient patient selection. Large language models (LLMs) have shown promise in various clinical tasks, but their potential role in RCT design remains underexplored.

**Objective:**

This study investigates the ability of LLMs, specifically GPT-4-Turbo-Preview, to assist in designing RCTs that enhance generalizability, recruitment diversity, and reduce failure rates, while maintaining clinical safety and ethical standards.

**Methods:**

We conducted a noninterventional, observational study analyzing 20 parallel-arm RCTs, comprising 10 completed and 10 registered studies published after January 2024 to mitigate pretraining biases. The LLM was tasked with generating RCT designs based on input criteria, including eligibility, recruitment strategies, interventions, and outcomes. The accuracy of LLM-generated designs was quantitatively assessed by 2 independent clinical experts by comparing them to clinically validated ground truth data from ClinicalTrials.gov. We have conducted statistical analysis using natural language processing–based methods, including Bilingual Evaluation Understudy (BLEU), Recall-Oriented Understudy for Gisting Evaluation (ROUGE)-L, and Metric for Evaluation of Translation with Explicit ORdering (METEOR), for objective scoring on corresponding LLM outputs. Qualitative assessments were performed using Likert scale ratings (1-3) for domains such as safety, clinical accuracy, objectivity or bias, pragmatism, inclusivity, and diversity.

**Results:**

The LLM achieved an overall accuracy of 72% in replicating RCT designs. Recruitment and intervention designs demonstrated high agreement with the ground truth, achieving 88% and 93% accuracy, respectively. However, LLMs showed lower accuracy in designing eligibility criteria (55%) and outcomes measurement (53%). Natural language processing statistical analysis reported BLEU=0.04, ROUGE-L=0.20, and METEOR=0.18 on average objective scoring of LLM outputs. Qualitative evaluations showed that LLM-generated designs scored above 2 points and closely matched the original designs in scores across all domains, indicating strong clinical alignment. Specifically, both original and LLM-based designs ranked similarly high in safety, clinical accuracy, and objectivity or bias in published RCTs. Moreover, LLM-based design ranked noninferior to original designs in registered RCTs in multiple domains. In particular, LLMs enhanced diversity and pragmatism, which are key factors in improving RCT generalizability and addressing failure rates.

**Conclusions:**

LLMs, such as GPT-4-Turbo-Preview, have demonstrated potential in improving RCT design, particularly in recruitment and intervention planning, while enhancing generalizability and addressing diversity. However, expert oversight and regulatory measures are essential to ensure patient safety and ethical standards. The findings support further integration of LLMs into clinical trial design, although continued refinement is necessary to address limitations in eligibility and outcomes measurement.

## Introduction

Randomized controlled trials (RCTs) serve as the backbone of modern evidence-based clinical practice [1]. RCT provides a carefully controlled environment to investigate cause-effect relationships between therapeutic intervention and clinical outcomes with a high degree of internal validity [2]. Over the years, landmark RCTs have significantly influenced treatment guidelines and improved global standards of care across various medical disciplines [3-5].

However, despite their scientific rigor in evidence, RCTs face persistent and well-documented criticisms of poor generalizability from fixed eligibility criteria [6], lack of diversification in recruitment [7], and practical implementation concerns [6]. Patients with complex comorbidities or late-stage diseases excluded from phase 3 trials fail to benefit from breakthrough discoveries in real-world practice. Thus, challenges need to be addressed to maximize the yield of each study.

In addition to concerns about representativeness, clinical trials face an alarmingly high failure rate, especially in the later stages of development. High failure rate of clinical trials is a key stumbling block in drug development pipelines. RCTs’ failure rate has been reported for various reasons [8-10], including safety and toxicity concerns, poor accrual and recruitment challenges, logistics, and funding. Of which, a key contributory factor to the failure of phase 3 trials is an inefficient patient selection process [11]. Failure of clinical trials bears significant implications for both drug development companies and patients. Clinical research remains the most expensive and time-consuming process of drug development, costing up to a billion dollars in investment and taking more than a decade of work to bring a new drug to market [12]. Reform of clinical research is much needed to accelerate this process.

Given the immense time, cost, and effort involved in clinical research, there is an urgent need to reform the RCT design process to address the aforementioned challenges. Emerging technologies, particularly large language models (LLMs), offer a novel opportunity to address these challenges. LLMs have recently emerged as an efficient tool in various clinical tasks [13] with comparable clinical alignment to human experts [14]. Developments in natural language processing (NLP) empowered LLMs to generate sophisticated and contextually relevant clinical content. Prominent examples, including GPT-4, Gemini, Llama 3, and Claude 3.5, have showcased remarkable versatility and clinical performance in highly specialized clinical tasks [15,16]. As a result, LLM tools are expected to assist clinical practice ranging from basic health care–related administrative work [17,18], educational chatbots for medical knowledge [19,20], to advanced clinical notes generation [21-23], complex clinical cases diagnosis [24], and patient triaging [25,26].

Recently, there has been increasing interest in LLM applications in clinical trials [27-30]. Generative artificial intelligence introduced new paradigms in drug development, from the design and validation of novel pharmaceutical compounds to eligibility screening of patients for clinical trials [27-29]. These approaches show promise in streamlining clinical research but fail to address problems related to trial design and generalizability of RCTs, including eligibility criteria, diversification, and practicability. RCTs provide the highest level of scientific evidence of therapeutic interventions, and their design requires in-depth clinical understanding and rigorous scientific methodologies [31-33].

In this study, we explore the application of LLMs as a tool for designing RCTs with clinical alignment and broader applicability. By piloting the use of LLMs in trial design, we aim to assess their potential to enhance the generalizability of study outcomes, optimize eligibility criteria, and ultimately reduce the failure rate of phase 3 clinical trials. This work contributes to the evolving dialogue on the future of clinical research and offers a practical pathway toward more inclusive, efficient, and evidence-driven trial methodologies.

## Methods

### Overview

We performed an observational, noninterventional study using GPT-4-Turbo-Preview as a state-of-the-art LLM for designing RCTs.

### Validation and Testing Datasets

We randomly selected 20 parallel-arm RCTs (phase 3 or 4): 10 completed RCTs, with results published in leading clinical journals (JAMA, Nature Medicine, NEJM, and The Lancet); and 10 RCTs registered on ClinicalTrials.gov. To mitigate the risks of LLMs’ pretraining use in such studies, we used studies published or newly registered after January 2024 (after the GPT-4-Turbo-Preview pretraining date of December 2023). Details of the dataset are presented in Table S1 in Multimedia Appendix 1.

### Reference Standard and LLM Prompt

We extracted the respective study designs from ClinicalTrials.gov (information cross-checked against publication if available), to serve as our ground truth. We provided the LLM with the following inputs: official titles, brief summaries, study type, study phase, study design, conditions, and intervention or treatment. We then prompted the LLM for the following outputs: eligibility criteria (inclusion and exclusion criteria), recruitment (sex or gender and age), arm or intervention (active and control arms), and outcomes measurement (measurement design and measurement time frame).

### Large Language Model

In this study, we selected GPT-4-Turbo-Preview. We chose a temperature of 0.2 to balance replicability and clinical rigor. Detailed prompts and output are presented in Figure S1 and Table S2 in Multimedia Appendix 1, respectively.

### Quantitative Evaluation

We quantitatively evaluated the accuracy (degree of agreement) of the LLMs’ outputs by comparing them with the clinically defined ground truth. We first collect ground truth for published studies from the publication (cross-examined with the corresponding study from ClinicalTrials.gov), and recent registered trials from ClinicalTrials.gov. For outputs with numerical or categorical answers, such as gender or age in recruitment and measurement time frame in outcome measures, we define correct answers as completely matching numerical values in the ground truth. For outputs with clinical answers, such as eligibility criteria, active and control arms in intervention, and measurement design in outcome measures, we defined answers as correct if clinically aligned with the ground truth. Specifically, for eligibility criteria designs, the accuracy was determined by the number of matched LLM designs divided by the total number of eligibility criteria listed by LLM.

We created a qualitative assessment metric to evaluate both LLM and ground truth designs. This metric comprised safety, clinical accuracy, objectivity (bias), pragmatic (adapted from PRECIS-2 guidance) [34], inclusivity, and diversity (adapted from United States Food and Drug Administration [FDA] draft guidance to clinical trial design) [7] measured on a 3-point Likert Scale (1 is the worst and 3 is the best). For selected registered RCT studies, we performed a blinded qualitative evaluation without knowledge of ground truth designs to provide a more objective analysis. Mean scores were calculated based on blinded human expert ratings stratified into RCTs (published and registered) with designs (ground truths and LLM designs).

### Statistical Analysis

We used average, nonweighted NLP-based objective scoring, including Bilingual Evaluation Understudy (BLEU), Recall-Oriented Understudy for Gisting Evaluation (ROUGE)-L, and Metric for Evaluation of Translation with Explicit ORdering (METEOR) for LLM outputs.

### Ethical Considerations

As this study is retrospective in nature and no real patient was involved in the current research, regulatory approval and informed consent are not applicable. Human clinical experts (reviewer 1–principal clinical pharmacist; reviewer 2–specialist physician in anesthesia, both with >10 years of clinical practice experience) received no compensation for rating.

## Results

**Figure 1.**
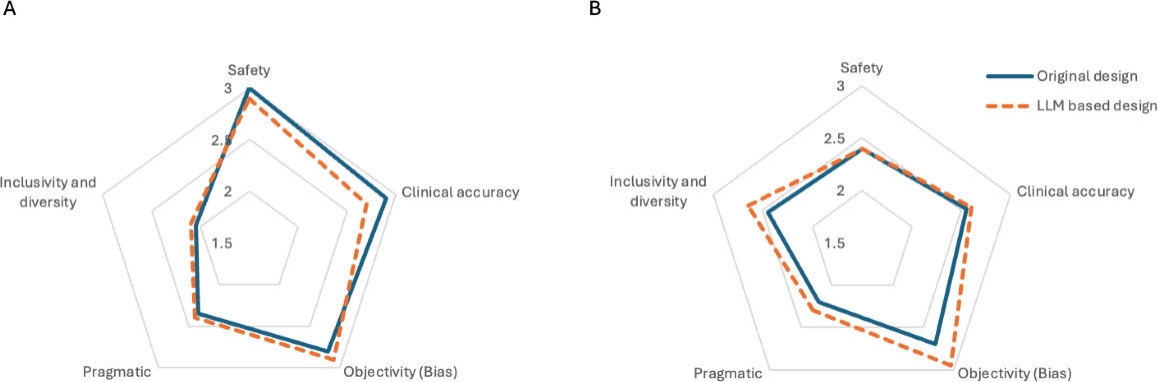
(A) Qualitative metrics for 10 published RCTs. (B) Qualitative metrics for 10 registered RCTs.

Our results show that LLM demonstrated 72% accuracy in overall RCT designs (stratified performance across different design domains is presented in Figure S2 in Multimedia Appendix 1). Specifically, it showed high agreement in Recruitment and Arm or Intervention, with accuracy of 88% and 93%, respectively. However, it demonstrated discrepancies in designing Eligibility Criteria and Outcomes Measurement, with an accuracy of 55% and 53%, respectively. We observed marginal differences in accuracy between LLM outputs and both published RCTs and registered RCTs, except for an improvement in exclusion criteria designs in the latest RCTs. We used statistical analysis using NLP-based methods, including BLEU [35], ROUGE-L [36], and METEOR [37], for corresponding LLM outputs, presented in Table S3 in Multimedia Appendix 1. Specifically, BLEU [35] measures n-gram precision to evaluate textual similarity, ROUGE-L [36] focuses on sequence recall and fluency by identifying the longest common subsequences, and METEOR [37] assesses semantic alignment and linguistic variability, incorporating synonyms, stemming, and word order. These metrics collectively provide a comprehensive evaluation of the generated outputs against the reference text. Qualitatively, LLM designs produced comparable clinical alignment, as observed in closely matched Likert scales, RCT design compared to ground truth, with Likert scales scoring above 2 points across all domains (Figure 1, grading scores were presented in Table S4 in Multimedia Appendix 1).

Our findings suggest that LLM, represented by GPT-4-Turbo-Preview in this study, can replicate RCT designs with reasonable clinical alignment. LLM was able to match RCTs with over 80% accuracy in designing Recruitment requirements and Active or Control Intervention. When assessed qualitatively, we observed marginal differences in the overall clinical accuracy of the LLM design compared with the ground truth, highlighting multiple accepted clinical decisions related to RCT design. Upon qualitative analysis, LLM-based RCT designs closely aligned with documented consensus in safe, accurate, and objective domains, while showing enhanced diversity and pragmatism. Notably, diversity and pragmatism are key determinants of LLM generalizability and reasons for RCT failure. In addition, LLM could avoid critical safety and ethical issues identified in the ground truth from the analysis of the selected registered RCTs.

## Discussion

### Principal Findings

RCTs serve key roles in clinical practice, and inclusivity has been heavily emphasized by the FDA [38] to ensure consistently high-quality design that is scientifically justifiable. Current results highlight the potential role of LLM for such an important design principle. Unique attributes of LLM architecture bring distinct advantages over conventional deep learning and NLP in text-based comprehension capabilities. General-purpose LLMs such as GPT-4 can perform tasks with little or no task-specific fine-tuning. Extensive pretraining on medically related free texts sets them apart from conventional machine learning or deep learning models, simulating clinical reasoning and inferential skills across diverse disciplines [39], allowing potential integration into sophisticated clinical tasks such as in clinical trial design. We infer that LLM could recommend the most commonly used comparator arms for trials of similar nature and discipline; logical deduction of active intervention dosage regimen based on preclinical or phase 1 and phase 2 published studies captured in its knowledge corpus.

Recommended exclusion criteria and outcome measurement time frames differed to a greater extent between LLM-designed trials and the actual published design. These design elements often vary widely across different studies and interventions tested in the real world. Qualitatively, the overall safety and clinical accuracy of these reported differences was not compromised significantly. Stronger performance in recruitment and intervention might be partially explained by the fact that LLMs are trained on previous examples of clinical trial designs, with better understanding in predicting sample sizes for inclusion and standard therapeutic intervention regimes. However, inferior performance in eligibility criteria designs and outcomes measurement emphasizes that critical clinical insights are necessary to facilitate clinically relevant clinical trial designs. Overall, LLM-based clinical trial designs might benefit more administrative aspects of clinical trial design, such as formulating standard intervention regimes and determining patient sample size, while further improvements are necessary to allow designs for highly specialized clinical trial–related domains. Coupled with further tailored RCT designs through prompting with LLMs regarding various patient and condition-related concerns, as well as financial and pragmatic challenges, the current pilot LLM-based RCT framework is expected to improve generalizability, enhance patient recruitment, and reduce RCT failure rates.

### Limitations

Our study has the following limitations. First, the generalizability of our findings is constrained by the specific LLM architecture used, GPT-4-Turbo-Preview, which may not reflect the performance of other LLMs or future versions. Although both human reviewers were experienced clinicians, the lack of a broader multidisciplinary review panel may limit the generalizability of the qualitative findings. Future studies could incorporate more diverse expert raters and a certified medical board. Our analysis was limited to text-based outputs, which do not capture the full complexity of clinical trial design, such as availability of funding, ease of patient recruitment, and ethical considerations. The study also relied on a relatively small sample of RCT designs, which may not provide a comprehensive view of the LLMs’ capabilities across diverse medical specialties. Future studies with larger sample sizes, expanding LLMs of interest for evaluation, and cost-effectiveness analysis stratified by various medical specialties are necessary. Furthermore, for phase 3 and phase 4 trials, substantial work including prior registration and funding would have been published and would affect the interpretation of this study toward the approach of LLM-based RCT designs. Future studies on LLM design from the initial hypothesis and direct comparison with concurrent human expert designs are necessary. Finally, alternative trial designs such as open-label, crossover, or pragmatic trials were not considered in this study.

### Comparison With Prior Work

To identify relevant studies, we used the following literature search strategy: (“clinical trials as topic” [MeSH Terms] OR “randomized controlled trials as topic” [MeSH Terms] OR “clinical trial” [Title or Abstract]) AND (“artificial intelligence” [MeSH Terms] OR “generative AI” [Title or Abstract] OR “language model” [Title or Abstract]) AND (2022:2024[pdat]). We restricted the search to articles published in PubMed between January 1, 2022, and April 1, 2024. We screened a total of 575 articles from PubMed and included a final total of 6 publications. We included peer-reviewed articles investigating the performance of generative artificial intelligence models applied in the conduct of clinical trials or RCTs. We excluded review papers and studies that did not report any model performance.

Existing clinical trial–related LLM studies, presented in Table 1, have only focused on preliminary text classification tasks and are mostly limited to last-generation LLMs, such as Bidirectional Encoder Representations from Transformers (BERT) [40]. For instance, performance over eligibility criteria recognition achieved a moderate *F*_1_-score over BERT-related LLMs [41]. AutoCriteria, leveraging GPT-4 in a zero-shot setting, significantly improved entity extraction across multiple diseases, highlighting the promise of the latest LLMs [42]. Other efforts include classifying exclusion criteria in cancer trials using BERT, again demonstrating LLM feasibility in clinical tasks [43]. GPT-4 has also been explored for sample size calculation, but observed inconsistencies underscore the need for caution in high-stakes applications [44]. In addition, predictive modeling of trial publication outcomes using BERT demonstrated the utility of LLM in combining structured and unstructured clinical trial data [45]. With rapid advancement in LLM development and taking advantage of LLMs’ accessibility and efficiency as demonstrated in this study, it holds great promise as an assistive tool for RCT design. In our quantitative analysis, LLMs could recommend study designs using gold standard control groups and appropriate active group interventions.

**Table 1.** Existing large language model applications in clinical trials−related studies.

Studies	LLM^a^ application	LLM^a^ base model	Testing dataset sample size	Evaluation metrics used	Model performance
A comparative study of pretrained language models for named entity recognition in clinical trial eligibility criteria from multiple corpora [41]	Eligibility screening	BERT^b^	470/230/1000	*F*_1_-score	0.72/0.84/0.62
AutoCriteria: a generalizable clinical trial eligibility criteria extraction system powered by large language models [42]	Eligibility screening	GPT-4^c^	180 trials	*F*_1_-score	0.90
Text classification of cancer clinical trial eligibility criteria [43]	Eligibility screening	BERT^b^	764 trials	ACC^d^	0.27‐0.95
ChatGPT for sample size calculation in sports medicine and exercise sciences: a cautionary note [44]	Sample size calculation	GPT 4^c^	4 trials	ACC	0.75
Medical text classification based on the discriminative pretraining model and prompt-tuning [46]	Assist trial outcome measurement	BERT^b^	5127 outcome entities	ACC	0.86
Predicting publication of clinical trials using structured and unstructured data: model development and validation study [45]	Trial outcome prediction	BERT^b^	76,950 trials	*F*_1_-score	0.70

a LLM: large language models

b BERT: Bidirectional Encoder Representations from Transformers

c GPT: Generative Pre-trained Transformer 4

d ACC: accuracy.

This study contributes significantly to the existing literature by providing empirical data on the accuracy and clinical alignment of LLMs specifically in the context of RCT design. Unlike previous studies, which primarily focus on preliminary text classification tasks, our research applied LLMs to the comprehensive design of RCTs, including elements such as eligibility criteria, recruitment strategies, and intervention arms. Our findings demonstrate that LLMs can replicate existing RCT designs with reasonable accuracy and add value by enhancing the diversity and pragmatism of trial designs. This is crucial in addressing common pitfalls in RCT generalizability and participant diversity. Various factors affect and influence clinical trial accessibility, and a comprehensive, multipronged approach is required. Other factors include the lack of education on the benefits of participating in clinical trials, patient trust, and the lack of incentives to participate [47]. The design of the clinical trial may inadvertently pose a barrier to entry. Clinical trials often exclude certain populations to a greater extent than others, such as patients with late-stage organ dysfunction.

Amid the growing interest in the use of LLMs to accelerate clinical trial processes, there is still a paucity of tools developed to improve the overall quality and inclusivity of clinical trials. Our study demonstrated that LLM is capable of assisting in trial design, encompassing elements of “best practices in clinical trial designs.” This can serve as a good reference point for nonsubject matter experts, including scientific review committees and ethics boards. Moving forward, the development of LLM-based agentic artificial intelligence workflows could further improve the utility and performance of LLMs in this application. Specialized LLM agents can be developed and incorporated into a multistep “checklist” approach to perform critical review and evaluation of various domains of a clinical trial design. Multiagent conversations have been shown to improve LLM output accuracy and mitigate cognitive bias [48].

### Conclusions

This study highlights the potential of LLMs to enhance RCT design, achieving substantial accuracy with key improvements in diversity and pragmatism. Such advancements could significantly improve the efficiency and effectiveness of clinical trials, driving faster development of therapeutic interventions. While LLMs show promise, expert oversight remains crucial for ensuring safety and ethics. Future efforts should aim to better integrate LLMs within clinical research frameworks and develop adaptive regulatory measures.

## Supplementary material

10.2196/67469 Multimedia Appendix 1 Supporting files on study design and evaluations.

## Abbreviations

BERT: Bidirectional Encoder Representations from Transformers
BLEU: bilingual evaluation understudy
FDA: US Food and Drug Administration
LLM: large language models
METEOR: Metric for Evaluation of Translation with Explicit Ordering
NLP: natural language processing
RCT: randomized controlled trial
ROUGE: Recall-Oriented Understudy for Gisting Evaluation
