# Supplementary material for: Large Language Models in Randomized Controlled Trials Design: Observational Study
Source: J Med Internet Res. 2025 Sep 3;27:e67469. doi: 10.2196/67469 (PMC12407223; doi:10.2196/67469)
Supplement: Multimedia Appendix 1 [file jmir-v27-e67469-s001.pdf]

Supplementary Materials

Figure S1: Prompt used for LLM based RCT design

As a clinician-scientist, you are tasked to design randomized control trials (RCT) based on following available Trial Description:

Initial Trial Description
Official Title:
A Phase 3, Multinational, Double-Blind, Randomized, Placebo-Controlled Study of MGL-3196 (Resmetirom) in Patients With Non-Alcoholic Steatohepatitis (NASH) and Fibrosis to Resolve NASH and Reduce Progression to Cirrhosis and/or Hepatic Decompensation

Brief Summary:
A double-blind placebo controlled randomized Phase 3 study to determine if 80 or 100 mg of MGL-3196 as compared with placebo resolves NASH and/or reduces fibrosis on liver biopsy and prevents progression to cirrhosis and/or advanced liver disease

Study Type:
Interventional

Study Phase:
Phase 3

Study Design:
Allocation:
Randomized
Interventional Model:
Parallel Assignment
Masking:
Quadruple (ParticipantCare ProviderInvestigatorOutcomes Assessor)
Primary Purpose:
Treatment

Conditions:
NASH - Nonalcoholic Steatohepatitis

Intervention / Treatment:
Drug: MGL-3196
Tablet
Other Names:
Resmetirom
Drug: Placebo
Matching Tablets

You are tasked to design:
Inclusion Criteria:
Exclusion Criteria:
Sex/Gender: (Male/Female/ALL)
Ages:
Arms and Interventions: Participant Group/Arm and its Intervention/Treatment
Primary Outcome Measures: Outcome Measure with its Measure Description and Time Frame
Secondary Outcome Measures: Outcome Measure with its Measure Description and Time Frame

Figure S2: LLMs’ outputs accuracy (degree of agreement when compared with clinic ground truth) on testing dataset. (10 completed RCTs and 10 registered RCTs).

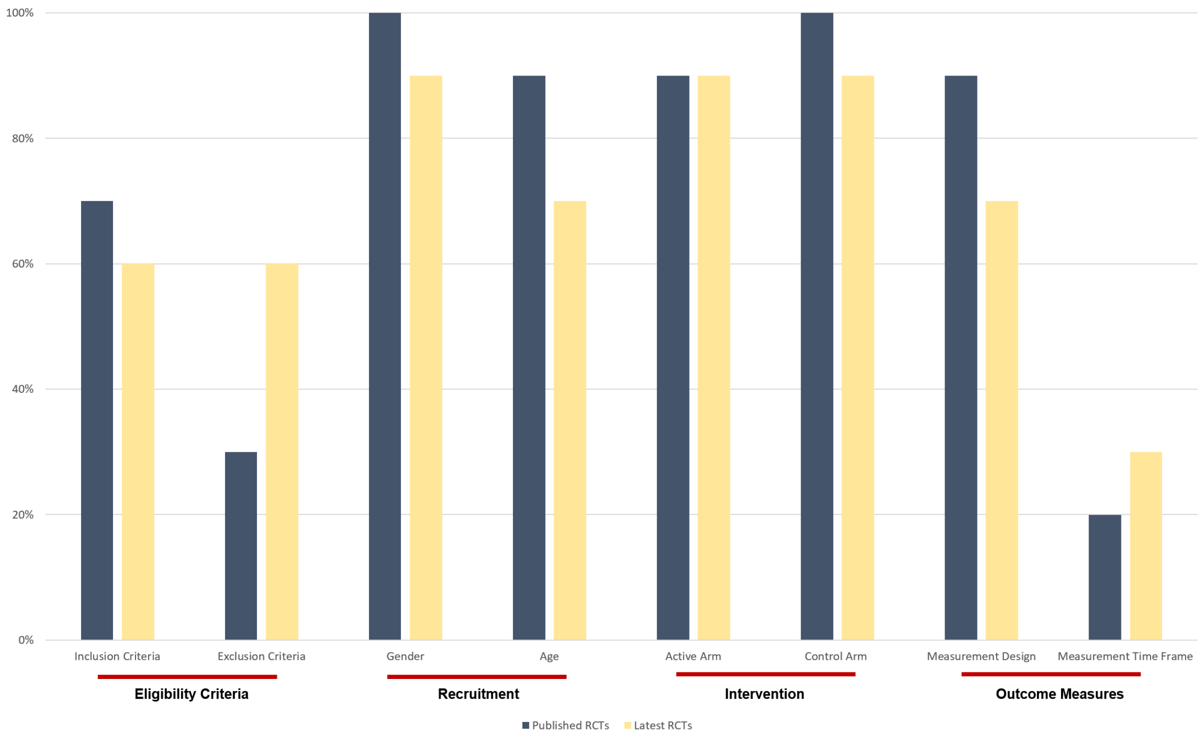

Table S1: Descriptions on selected RCTs. No. 1-10 are published RCTs; No. 11-20 are registered RCTs.

NO	JOURNAL	PUBLISH YEAR	RCT NAME	SPECIALITY	PHASE	FIRST POSTED DATE
1	NEJM	2024/2	A Phase 3 Study to Evaluate the Efficacy and Safety of MGL-3196 (Resmetirom) in Patients With NASH and Fibrosis (MAESTRO-NASH)	Gastroenterology	III	NA
2	NEJM	2024/1	Testosterone Treatment and Fractures in Men with Hypogonadism	Endocrine	IV	NA
3	NEJM	2024/1	Azithromycin during Routine Well-Infant Visits to Prevent Death	Paediatric	IV	NA
4	NEJM	2024/1	Efficacy and Safety of Acoramidis in Transthyretin Amyloid Cardiomyopathy	Cardiology	III	NA
5	JAMA	2024/1	Continued Treatment With Tirzepatide for Maintenance of Weight Reduction in Adults With Obesity The SURMOUNT-4 Randomized Clinical Trial	Endocrine	III	NA
6	The Lancet	2024/2	Clinical Efficacy of Typhoid Conjugate Vaccine (Vi-TCV) Among Children Age 9 Months Through 12 Years in Blantyre, Malawi	Infectious Disease	III	NA
7	The Lancet	2024/1	Chemoprevention for malaria with monthly intermittent preventive treatment with dihydroartemisinin–piperaquine in pregnant women living with HIV on daily co-trimoxazole in Kenya and Malawi: a randomised, double-blind, placebo-controlled trial	Infectious Disease	III	NA
8	The Lancet	2024/1	A Phase III Study of Safety and Efficacy of Ligelizumab in the Treatment of CSU in Adolescents and Adults Inadequately Controlled With H1-antihistamines	Dermatology	III	NA
9	The Lancet	2024/1	Efficacy and safety of the muscarinic receptor agonist KarXT (xanomeline–trospium) in schizophrenia (EMERGENT-2) in the USA: results from a randomised, double-blind, placebo-controlled, flexible-dose phase 3 trial	Psychiatric	III	NA
10	Nature Medicine	2024/1	First-line talazoparib with enzalutamide in HRR-deficient metastatic castration-resistant prostate cancer: the phase 3 TALAPRO-2 trial	Oncology	III	NA
11	NA	NA	Colchicine in Acutely Decompensated HFREF	Cardiology	IV	2/29/2024
12	NA	NA	Clinical Trial of the Efficacy and Safety of Raphamin in Prevention of	Infectious Disease	III	2/28/2024

			Recurrences of Chronic Bacterial Cystitis			
13	NA	NA	A Phase 3 Study of LNK01001 Capsule in Moderately to Severely Active Rheumatoid Arthritis	Rheumatology	III	2/26/2024
14	NA	NA	Comparison of Postoperative Pain Score Between Perioperative Intravenous Ketamine and Placebo in Patients Undergoing Unilateral Total Knee Arthroplasty Under General Anesthesia	Anesthesia	IV	2/20/2024
15	NA	NA	Clinical Trial of the Efficacy and Safety of Raphamin in Combined Treatment of Community-acquired Pneumonia	Infectious Disease	III	2/16/2024
16	NA	NA	A Study of Guselkumab in Pediatric Participants With Moderately to Severely Active Ulcerative Colitis (QUASAR Jr)	Gastroenterology	III	2/15/2024
17	NA	NA	A Study to Evaluate Mavacamten in Adolescents With Symptomatic Obstructive Hypertrophic Cardiomyopathy	Cardiology	III	2/12/2024
18	NA	NA	A Study to Investigate the Effects of PT027 (Budesonide/Albuterol Sulfate) Metered-dose Inhaler Compared With Placebo on Exercise-Induced Bronchoconstriction in Adult Patients With Asthma (BREATH)	Respiratory	III	2/7/2024
19	NA	NA	Perfenidone in Type 2 Diabetic Patients With Diabetic Neuropathy (PenDaNt)	Endocrine	IV	1/25/2024
20	NA	NA	A Study to Assess Long-term Safety of Fezolinetant Given to Japanese Women Going Through Menopause (Starlight 3)	Obstetrics and gynaecology	III	1/16/2024

Table S2: Inputs and outputs for LLMs-based clinical trial designs, with corresponding clinical ground truth.

Study Number	Input	Output	Ground Truth
1	Initial Trial Description<br><br>Official Title: A Phase 3, Multinational, Double-Blind, Randomized, Placebo-Controlled Study of MGL-3196 (Resmetirom) in Patients With Non-Alcoholic Steatohepatitis (NASH) and Fibrosis to Resolve NASH and Reduce Progression to Cirrhosis and/or Hepatic Decompensation<br><br>Brief Summary: A double-blind placebo controlled randomized Phase 3 study to determine if 80 or 100 mg of MGL-3196 as compared with placebo resolves NASH and/or reduces fibrosis on liver biopsy and prevents progression to cirrhosis and/or advanced liver disease<br><br>Study Type: Interventional<br><br>Study Phase: Phase 3<br><br>Study Design:<br>Allocation: Randomized<br>Interventional Model: Parallel Assignment<br>Masking: Quadruple (Participant Care	Inclusion Criteria:<br>Adults aged 18-70 years.<br>Histologically confirmed diagnosis of NASH with fibrosis stages 2 or 3, according to the NASH Clinical Research Network (CRN) Histologic Scoring System.<br>Body Mass Index (BMI) between 25 and 40 kg/m^2.<br>Stable body weight (±5%) for at least 3 months prior to screening.<br>Willingness to adhere to the study protocol and follow-up schedule.<br><br>Exclusion Criteria:<br>Evidence of other forms of chronic liver diseases such as alcoholic liver disease, hepatitis B, or hepatitis C.<br>History of liver transplantation or current listing for liver transplantation.<br>Decompensated liver disease, indicated by the presence of ascites, encephalopathy, or variceal bleeding.<br>Use of other investigational drugs within 30 days prior to screening.<br>Pregnant or breastfeeding women.<br><br>Sex/Gender: ALL<br><br>Ages: 18-70 years<br><br>Arms and Interventions:	Inclusion Criteria:<br>Must be willing to participate in the study and provide written informed consent.<br>Male and female adults ≥ 18 years of age.<br>Suspected or confirmed diagnosis of NASH fibrosis suggested by the historical data. Meet one of the following criteria that is consistent with NASH liver fibrosis:<br>Historical biochemical test for fibrosis: PRO-C3 >14 ng/mL or ELF ≥9<br>FibroScan with transient elastography ≥8.5 kPa and controlled attenuation parameter ≥280 dB.m-1<br>Historical liver biopsy obtained <2 years before expected randomization showing Stage 1B, 2 or 3 fibrosis with NASH based on existing pathology review, with no significant change in body weight >5% or medication that might affect NAS or fibrosis stage.<br>MRI-PDFF fat fraction ≥8% obtained during the screening period<br>Biopsy-proven NASH (baseline liver biopsy) based on a liver biopsy obtained ≤6 months before anticipated date of randomization (if the biopsy is deemed acceptable for interpretation by the central reader) with fibrosis stage 1A/1C, 1B, 2, or 3 on liver biopsy and NAS of ≥4 with a score of at least 1 in each of the following NAS components:<br>Steatosis (scored 0 to 3)<br>Ballooning degeneration (scored 0 to 2)<br>Lobular inflammation (scored 0 to 3)<br><br>Exclusion Criteria:<br>History of significant alcohol consumption for a period of more than 3 consecutive months within 1 year prior to Screening.<br>Regular use of drugs historically associated with NAFLD<br>Thyroid diseases:

	Provider Investigator Outcomes Assessor)<br>Primary Purpose: Treatment<br><br>Conditions:<br>NASH - Nonalcoholic Steatohepatitis<br><br>Intervention / Treatment:<br>Drug: MGL-3196 Tablet<br>Other Names: Resmetirom<br>Drug: Placebo Matching Tablets	Participant Group/Arm 1: MGL-3196 (Resmetirom) 100 mg tablet, orally, once daily.<br>Participant Group/Arm 2: Placebo matching tablet, orally, once daily.<br><br>Primary Outcome Measures:<br>Outcome Measure: Resolution of NASH without worsening of fibrosis.<br>Measure Description: Proportion of participants achieving histological resolution of NASH (no NASH) on liver biopsy without worsening of fibrosis.<br>Time Frame: At 48 weeks.<br><br>Secondary Outcome Measures:<br>Outcome Measure: Improvement in liver fibrosis.<br>Measure Description: Proportion of participants with an improvement in liver fibrosis by at least one stage without worsening of NASH on liver biopsy.<br>Time Frame: At 48 weeks.<br>Outcome Measure: Change in liver enzymes (ALT, AST).<br>Measure Description: Mean change in Alanine Aminotransferase (ALT) and Aspartate Aminotransferase (AST) levels from baseline.<br>Time Frame: At 24 and 48 weeks.<br><br>Clinical Reasoning:<br><br>Inclusion Criteria:<br>Age and BMI criteria ensure the study	Active hyperthyroidism.<br>Untreated clinical hypothyroidism defined by thyroid stimulating hormone (TSH) >7 IU/L with symptoms of hypothyroidism or >10 IU/L without symptoms.<br>Patients who have had a thyroidectomy and are on replacement thyroxine doses >75 µg per day are allowed.<br>History of bariatric surgery or intestinal bypass surgery within the 5 years prior to randomization or planned during the conduct of the study.<br>Recent significant weight gain or loss HbA1c ≥ 9.0%.<br>Glucagon-like peptide 1 [GLP-1] agonist, high dose Vitamin E (> 400 IU/day), or pioglitazone therapy unless stable dose for 24 weeks prior to biopsy.<br>Presence of cirrhosis on liver biopsy defined as stage 4 fibrosis.<br>Diagnosis of hepatocellular carcinoma (HCC).<br>MELD score ≥12, as determined at Screening, unless due to therapeutic anti coagulation.<br>Hepatic decompensation<br>Chronic liver diseases other than NASH<br>Active autoimmune disease<br>Serum ALT > 250 U/L.<br>Active, serious medical disease with a likely life expectancy < 2 years.<br>Participation in an investigational new drug trial in the 60 days or 5 half-lives, whichever is longer.<br>Any other condition which, in the opinion of the Investigator, would impede compliance, hinder completion of the study, compromise the well-being of the patient, or interfere with the study outcomes.<br><br>Sex/Gender: All<br><br>Ages: 18 Years and older (Adult, Older Adult)<br><br>Enrollment size (Estimated): 2000<br><br>Arms and Interventions:

		population is representative of typical NASH patients, who are generally middle-aged and overweight or obese.<br>Histological confirmation of NASH with specific fibrosis stages ensures a homogeneous study population with a significant but not end-stage disease, maximizing the potential to observe treatment effects.<br><br>Exclusion Criteria:<br>2Excluding other liver diseases ensures that observed effects are attributable to the intervention and not confounded by other conditions.<br>Excluding those with decompensated liver disease focuses the study on patients more likely to benefit from intervention before reaching a point of irreversible liver damage.<br><br>Sex/Gender and Ages:<br>Including all genders and a wide age range ensures broad applicability of study results.<br><br>Arms and Interventions:<br>Two arms allow for direct comparison between the drug and placebo, essential for assessing drug efficacy.<br>The choice of dosage is based on previous phases of clinical trials that likely identified this dose as effective with manageable side effects.	Placebo Comparator: Matching Placebo<br>Placebo Daily<br>Interventions:<br>Drug: Placebo<br>Active Comparator: 80 mg MGL-3196<br>80 mg daily<br>Interventions:<br>Drug: MGL-3196<br>Active Comparator: 100 mg MGL-3196<br>100 mg daily<br>Interventions:<br>Drug: MGL-3196<br><br>Primary Outcome Measures:<br>Week 52 Dual Primary Objectives: To determine the effect of 80 or 100 mg MGL-3196 vs matching placebo on liver biopsy (NASH CRN score) at Week 52 compared with Baseline [Time Frame: 52 weeks]<br>Proportion with resolution of NASH (ballooning 0, inflammation 0,1) associated with at least 2-point reduction in NAFLD Activity Score (NAS) without worsening of fibrosis stage OR<br>Proportion with at least a 1-point improvement in fibrosis stage with no worsening of NAS<br>Month 54 Primary Objective: Time to experiencing an adjudicated Composite Clinical Outcome event (Final Primary Endpoint, at 54 months) [Time Frame: up to 54 months]<br>The Composite Clinical Outcome is composed of all-cause mortality, liver transplant, and significant hepatic events (including hepatic decompensation events [ascites, encephalopathy, or gastroesophageal variceal hemorrhage], histological progression to cirrhosis, and a confirmed increase of MELD score from <12 to ≥15).<br><br>Secondary Outcome Measures:<br>Week 52 Key Secondary Objective: To determine the effect of once-daily, oral administration of MGL-3196 80 or 100 mg versus matching placebo on the percent change from Baseline at 24 weeks in directly measured low-

		Primary and Secondary Outcome Measures:<br>The primary outcome directly addresses the study's goal of resolving NASH and reducing fibrosis, which are clinically meaningful endpoints.<br>Secondary outcomes provide additional information on the drug's impact on liver health and function, offering a comprehensive view of its potential benefits.<br><br>This trial design aims to rigorously test the efficacy and safety of MGL-3196 in a well-defined NASH patient population, with clear, relevant outcomes that align with the goals of treatment in this condition.	density lipoprotein cholesterol (LDL-C) [Time Frame: 24 weeks]<br>Assess the effect of MGL-3196 80 mg or 100 mg compared to placebo on LDL-C measured by percent change from Baseline at 24 weeks.
2	Initial Trial Description<br>Official Title: Testosterone Replacement Therapy for Assessment of Long-term Vascular Events and Efficacy ResponSE in Hypogonadal Men (TRAVERSE) Study<br><br>Brief Summary: This is a blinded and placebo-controlled study of topical testosterone replacement therapy (TRT) in symptomatic hypogonadal men with increased risk for cardiovascular (CV) disease.<br><br>Study Type: Interventional	Inclusion Criteria:<br>Male participants aged 45-80 years.<br>Clinically diagnosed with symptomatic hypogonadism (total testosterone levels below 300 ng/dL on two separate mornings).<br>Documented increased risk for cardiovascular disease (e.g., history of coronary artery disease, diabetes mellitus, hypertension, or hyperlipidemia).<br>Willing and able to provide informed consent.<br><br>Exclusion Criteria:<br>History of prostate or breast cancer.<br>Severe heart failure (NYHA Class III or IV).	Inclusion Criteria:<br>Men between 45 and 80 years age<br>Participants with low serum testosterone concentrations (< 300 ng/dL) who exhibit at least one sign or symptom of hypogonadism and have evidence of cardiovascular (CV) disease or are at an increased risk for CV disease.<br><br>Exclusion Criteria:<br>Participants with congenital or acquired hypogonadism for whom long-term therapy with placebo would not be medically appropriate<br>Participants with prostate specific antigen (PSA) > 3.0 ng/mL (or 1.5 if on 5-alpha reductase inhibitors)<br>Participants who have been treated with testosterone in the past 6 months and for whom testosterone therapy is contraindicated<br>Confirmed testosterone < 100 ng/dL<br>Body Mass Index (BMI) > 50<br>Hemoglobin A1c (HbA1C) > 11%<br>Hematocrit (Hct) > 50%

	Study Phase: Phase 4<br><br>Study Design:<br>Allocation: Randomized<br>Interventional Model: Parallel Assignment<br>Masking: Quadruple (Participant Care Provider Investigator Outcomes Assessor)<br>Primary Purpose: Treatment<br><br>Conditions:<br>Hypogonadism<br>Cardiovascular Diseases<br><br>Intervention / Treatment:<br>Drug: Testosterone. It will be administered topically.<br>Other Names: Androgel®<br>Drug: Placebo. A matching placebo to testosterone will be administered topically.	History of deep vein thrombosis or pulmonary embolism.<br>Use of any other form of testosterone replacement therapy or anabolic steroids within the last 6 months.<br>Uncontrolled hypertension (BP > 160/100 mmHg).<br><br>Sex/Gender: Male<br><br>Ages: 45-80 years<br><br>Enrollment size (Estimated): 600<br><br>Arms and Interventions:<br>Participant Group/Arm 1: Testosterone Group<br>Intervention/Treatment: Drug: Testosterone (Topical application, daily for 24 months)<br>Participant Group/Arm 2: Placebo Group<br>Intervention/Treatment: Drug: Placebo (Topical application, matching placebo, daily for 24 months)<br><br>Primary Outcome Measures:<br>Outcome Measure: Major adverse cardiovascular events (MACE)<br>Measure Description: Composite outcome of non-fatal myocardial infarction, non-fatal stroke, and cardiovascular death.<br>Time Frame: From baseline to 24 months.<br><br>Secondary Outcome Measures:<br>Outcome Measure: Change in total testosterone levels	Estimated Glomerular Filtration Rate (eGFR) < 30 ml/min<br>History of deep vein thrombosis or pulmonary embolism or prostate cancer or heart failure (Class III and IV).<br><br>Sex/Gender: Male<br><br>Ages: 45 Years to 80 Years (Adult, Older Adult)<br><br>Enrollment size (Estimated): 6000<br><br>Arms and Interventions:<br>Active Comparator: Arm A<br>Participants receiving topical testosterone<br>Interventions:<br>Drug: Testosterone<br>Placebo Comparator: Arm B<br>Participants receiving placebo<br>Interventions:<br>Drug: Placebo<br><br>Primary Outcome Measures:<br>Time to Major Adverse Cardiac Event (MACE) [Time Frame: Up to study completion (approximately month 60)]<br>It is defined as time from randomization to first component event occurrence of the composite MACE endpoint. MACE is defined as a composite endpoint consisting of any of the following: nonfatal myocardial infarction (MI), nonfatal stroke or death due to cardiovascular (CV) causes.<br><br>Secondary Outcome Measures:<br>Assessing cardiovascular safety [Time Frame: Up to study completion (approximately month 60)]<br>Cardiovascular safety is time from randomization to first component event occurrence of the composite endpoint consisting of the following: nonfatal MI, nonfatal stroke, death due to CV causes or cardiac revascularization procedures/ cardiac percutaneous coronary intervention (PCI) and coronary artery bypass graft (CABG).

		Measure Description: Change from baseline in serum total testosterone levels.<br>Time Frame: Baseline, 6, 12, 18, and 24 months.<br>Outcome Measure: Quality of life assessment<br>Measure Description: Measured by the Aging Males' Symptoms (AMS) scale.<br>Time Frame: Baseline, 12, and 24 months.<br><br>Clinical Reasoning:<br><br>Inclusion Criteria:<br>The age range is selected to include a population at significant risk for both hypogonadism and cardiovascular disease.<br>The requirement for symptomatic hypogonadism ensures that participants are likely to benefit from TRT.<br>Including men with increased CV risk allows for the assessment of TRT's safety and efficacy in a high-risk group.<br><br>Exclusion Criteria:<br>Excluding those with a history of hormone-sensitive cancers minimizes the risk of cancer progression.<br>Excluding severe heart failure patients ensures the safety of participants, as TRT might exacerbate heart failure.<br>The exclusion of recent anabolic steroid use ensures that baseline testosterone levels are	Assessing prostate safety [Time Frame: Up to study completion (approximately month 60)]<br>It is assessed by the incidence of high grade prostate cancer.

		not influenced by external factors.<br><br>Sex/Gender:<br>The study focuses on male hypogonadism, hence only males are included.<br><br>Ages:<br>The chosen age range targets older adults who are more likely to have both hypogonadism and increased cardiovascular risk.<br><br>Enrollment size:<br>A sample size of 600 is estimated to provide adequate power to detect a difference in the primary outcome, considering potential dropouts and the event rate for MACE.<br><br>Arms and Interventions:<br>A parallel design with a testosterone and placebo group allows for a clear comparison of the effects of TRT.<br>The use of a topical application is consistent with common clinical practice for TRT.<br><br>Primary and Secondary Outcome Measures:<br>MACE is chosen as the primary outcome to directly assess the cardiovascular safety of TRT.<br>Monitoring testosterone levels and quality of life provides insight into the efficacy and impact of TRT on well-being.<br><br>This design aims to rigorously evaluate the long-term cardiovascular safety and efficacy of	

		TRT in symptomatic hypogonadal men with increased cardiovascular risk, addressing a significant gap in current knowledge.	
3	Initial Trial Description<br>Official Title: Community Health Azithromycin Trial in Burkina Faso<br><br>Brief Summary: An estimated 7.7 million pre-school aged children die each year, the majority from infectious diseases. Mass azithromycin distributions for trachoma may have the unintended benefit of reducing childhood mortality. We recently demonstrated the biannual mass azithromycin distribution significantly reduces all-cause child mortality in a cluster randomized trial (MORDOR I) conducted in three diverse regions of Sub-Saharan Africa. Our long-term goal is to more precisely define the role of mass azithromycin treatments as an intervention for reducing childhood morbidity and mortality. We propose a cluster randomized trial designed to repeat the original study to confirm the original results in a different geographic study with similarly	Inclusion Criteria:<br>Children aged 1 to 59 months for the community-wide intervention.<br>Infants aged approximately 5 to 12 weeks (targeted intervention during early infant healthcare visit).<br>Resident of the Nouna District, Burkina Faso.<br><br>Exclusion Criteria:<br>Known allergy or contraindication to azithromycin.<br>Participation in another clinical trial that could interfere with the outcomes of this study.<br>Severe illness requiring hospitalization at the time of enrollment.<br><br>Sex/Gender: ALL<br><br>Ages:<br>1 to 59 months for the community-wide intervention.<br>Approximately 5 to 12 weeks for the targeted intervention.<br><br>Enrollment size (Estimated): 10,000<br><br>Arms and Interventions:<br>Participant Group/Arm 1: Biannual azithromycin distribution to children 1 to 59 months old in eligible communities.<br>Intervention/Treatment for Arm 1: Drug: Azithromycin.<br>Participant Group/Arm 2: Biannual placebo	Inclusion Criteria:<br>Communities:<br>The community location in target district.<br>The community leader consents to participation in the trial (this does not obviate the need for individual consent, but without overall leadership consent, the community as a whole cannot be part of the trial).<br>Eligible communities estimated population of between 200-2,000 people<br>The community is not in an urban area<br><br>Individuals:<br>All children in the study communities aged 5 to 12 weeks old at the time of the vaccination visit are eligible to participate<br>Ability to feed orally<br>Appropriate consent from at least one caregiver<br>Family intends to stay within the study area<br><br><br>Exclusion criteria:<br>Communities:<br>Refusal of village chief<br><br>Individuals:<br>Individuals allergic to macrolides or azalides will not be given the study antibiotic azithromycin, but will be included in the outcome<br>Refusal of parent or guardian<br>Child unable to orally feed<br>Family planning to move<br>Children younger than 28 days old or older than 12 weeks<br>Children in the bi annual drug administration group who weight less than 3.8kg.<br><br>Sex/Gender: All

	high child mortality, and to better understand the mechanism behind any effect of azithromycin on child mortality. We hypothesize that biannual mass azithromycin distribution will reduce child mortality compared to placebo, and that this effect will be primarily driven by a reduction in infectious burden. Objectives:Determine the efficacy of biannual mass azithromycin distribution versus placebo in children aged 1-59 months for reduction in all-cause mortality.<br>Determine the efficacy of targeted azithromycin distribution to infants during an early infant healthcare visit (approximately 5th through 12th week of life) on infant mortality. Determine the mechanism behind the effect of biannual mass azithromycin distribution for reduction in child mortality. The study will be conducted in the Nouna District in northwestern Burkina Faso.<br><br>Study Type: Interventional<br><br>Study Phase: Phase 4<br><br>Study Design:	distribution to children 1 to 59 months old in eligible communities.<br>Intervention/Treatment for Arm 2: Drug: Placebos.<br>Participant Group/Arm 3: Targeted azithromycin distribution to infants aged approximately 5 to 12 weeks old at the vaccine visit.<br>Intervention/Treatment for Arm 3: Drug: Azithromycin.<br>Participant Group/Arm 4: Targeted placebo distribution to infants aged approximately 5 to 12 weeks old at the vaccine visit.<br>Intervention/Treatment for Arm 4: Drug: Placebos.<br><br>Primary Outcome Measures:<br>Outcome Measure: All-cause mortality rate.<br>Measure Description: The number of deaths from any cause among the enrolled children during the study period.<br>Time Frame: 24 months.<br><br>Secondary Outcome Measures:<br>Outcome Measure: Incidence of infectious diseases.<br>Measure Description: The number of new cases of specified infectious diseases (e.g., respiratory infections, diarrheal diseases) among the enrolled children during the study period.<br>Time Frame: 24 months.<br>Outcome Measure: Growth and nutritional status.	Ages: 1 Month to 59 Months (Child)<br><br>Enrollment size (Estimated): 447780<br><br>Arms and Interventions:<br>Active Comparator: Biannual mass oral azithromycin<br>Bi-annual Mass Azithromycin distribution to all children 1-60 months old in participating communities<br>Interventions:<br>Drug: Azithromycin<br>Placebo Comparator: Biannual mass oral placebo<br>Bi-annual Mass Placebo distribution to all children 1-60 months old in participating communities<br>Interventions:<br>Drug: Azithromycin<br>Drug: Placebos<br>Placebo Comparator: Targeted oral placebo<br>Targeted placebo to children 5 to 12 weeks old at vaccine visit or other healthy child visit<br>Interventions:<br>Drug: Placebos<br>Active Comparator: Targeted oral azithromycin<br>Targeted azithromycin to children 5 to 12 weeks old at vaccine visit or other healthy child visit<br>Interventions:<br>Drug: Azithromycin<br><br>Primary Outcome Measures:<br>All-cause Mortality Rate in children aged 1-59 months [Time Frame: 36 months]<br>All-cause mortality as determined by biannual census among children aged 1-59 months<br>All-cause Mortality Rate in individually randomized children at 4-12 weeks of age [Time Frame: 6 months]<br>All-cause mortality as determined by a follow-up visit for individually randomized children at healthy child visits<br><br>Secondary Outcome Measures:

	Allocation: Randomized<br>Interventional Model: Factorial Assignment<br>Interventional Model Description: All eligible communities in Nouna District will be randomized in a 1:1 fashion to biannual azithromycin or placebo. Targeted treatment (vaccine visit) will be randomized 1:1 individually to azithromycin or placebo. Randomization will be conducted by T. Porco. Procedural and algorithmic details are provided in an appendix to the Statistical Analysis Plan.<br>Masking: Quadruple (Participant Care Provider Investigator Outcomes Assessor)<br>Masking Description: The trial sites will be masked to outcomes, so the responsibility for monitoring interim analysis will fall on the DSMC<br>Primary Purpose: Prevention<br><br>Conditions: Childhood Mortality<br><br>Intervention / Treatment:<br>Drug: Azithromycin. Biannual azithromycin in eligible communities to children 1 to 59 months old Targeted azithromycin to children aged 5 to 8 weeks old at the vaccine visit	Measure Description: Changes in weight-for-age and height-for-age Z-scores among the enrolled children during the study period.<br>Time Frame: 24 months.<br><br>Clinical Reasoning:<br><br>Inclusion and Exclusion Criteria:<br>The age criteria are based on the target population most likely to benefit from azithromycin interventions, as indicated by previous studies. Excluding children with severe illness ensures the safety of participants and the integrity of the study results.<br><br>Sex/Gender and Ages:<br>Including all genders and specifying age groups ensures that the study results are generalizable to the entire population of children at risk of high mortality due to infectious diseases in the study area.<br><br>Enrollment Size:<br>The estimated size is designed to ensure the study has adequate power to detect a significant difference in mortality, considering the baseline mortality rates and the effect size observed in previous studies.<br><br>Arms and Interventions:<br>The factorial design allows for the assessment of both	Malaria parasitemia in children 1-59 months at 36 months [Time Frame: 36 months]<br>Malaria parasitemia as measured by thin and thick smears in a random sample of children at 36 months<br>Weight-for-height Z-score in individually randomized children at healthy child visits [Time Frame: 6 months]<br>Height-for-age Z-score in individually randomized children at healthy child visits [Time Frame: 6 months]<br>Mid-upper arm circumference in individually randomized children at healthy child visits [Time Frame: 6 months]<br>Linear growth in individually randomized children [Time Frame: 6 months]<br>Change in length from baseline to 6 months<br>Weight gain in individually randomized children [Time Frame: 6 months]<br>Change in weight from baseline to 6 months

	Drug: Placebos. Biannual placebo in eligible communities to children 1 to 59 months old Targeted placebo to children aged 5 to 8 weeks old at the vaccine visit	community-wide and targeted interventions, maximizing the study's potential to provide actionable insights.<br><br>Outcome Measures:<br>The primary outcome directly addresses the study's main objective. Secondary outcomes are chosen to provide insights into potential mechanisms behind the effect of azithromycin on mortality, such as reductions in specific infectious diseases and improvements in nutritional status.	
4	Initial Trial Description<br>Official Title: A Phase 3, Randomized, Double-Blind, Placebo-Controlled Study of the Efficacy and Safety of AG10 in Subjects With Symptomatic Transthyretin Amyloid Cardiomyopathy (ATTRibute-CM Trial)<br><br>Brief Summary:<br>Phase 3 efficacy and safety study to evaluate acoramidis (AG10) HCl 800 mg administered orally twice a day compared to placebo in subjects with symptomatic Transthyretin Amyloid Cardiomyopathy (ATTR-CM).<br><br>Study Type: Interventional<br><br>Study Phase: Phase 3<br><br>Study Design:<br>Allocation:	Inclusion Criteria:<br>Adults aged 18 years or older.<br>Clinically diagnosed with Transthyretin Amyloid Cardiomyopathy (ATTR-CM) confirmed by biopsy or non-biopsy based methods (e.g., scintigraphy).<br>Symptomatic heart failure (NYHA Class II-III).<br>Stable heart failure medication regimen for at least 30 days prior to enrollment.<br>Ejection fraction ≥40%.<br><br>Exclusion Criteria:<br>Known hypersensitivity to acoramidis (AG10) or any of its excipients.<br>Participation in another interventional study within 30 days prior to enrollment.<br>Severe renal impairment (eGFR <30 mL/min/1.73 m^2) or dialysis.<br>Liver disease with ALT or AST >3 times the upper limit of normal.<br>Pregnant or breastfeeding women.	Inclusion Criteria:<br>Have an established diagnosis of ATTR-CM with either wild-type TTR or variant TTR genotype<br>Have a history of heart failure evidenced by at least one prior hospitalization for heart failure or clinical evidence of heart failure without prior heart failure hospitalization manifested by signs or symptoms of volume overload or elevated intracardiac pressures or heart failure symptoms that required or require ongoing treatment with a diuretic.<br>New York Heart Association (NYHA) Class I-III symptoms due to ATTR cardiomyopathy.<br>On stable doses of cardiovascular medical therapy<br>Completed ≥150 m on the 6MWT on 2 tests that are within 15% of total distance walked prior to randomization<br>Biomarkers of myocardial wall stress, NT-proBNP level ≥300 pg/mL at screening<br>Have left ventricular wall (interventricular septum or left ventricular posterior wall) thickness ≥12 mm<br><br>Exclusion Criteria:<br>Had acute myocardial infarction, acute coronary syndrome or coronary

	Randomized<br>Interventional Model: Parallel Assignment<br>Masking: Quadruple (Participant Care Provider Investigator Outcomes Assessor)<br>Primary Purpose: Treatment<br><br>Conditions:<br>Amyloidosis<br>Amyloid Cardiomyopathy<br>Transthyretin Amyloidosis<br>Cardiomyopathies<br>Heart Diseases<br><br>Intervention / Treatment:<br>Drug: acoramidis.<br>TTR stabilizer administered orally twice daily (BID)<br>Other Names:<br>AG10<br>ALXN2060<br>Drug: Placebo Oral Tablet<br>Non-active control administered orally twice daily (BID)	Sex/Gender: ALL<br><br>Ages: 18 years and older<br><br>Enrollment size (Estimated): 400<br><br>Arms and Interventions:<br>Participant Group/Arm 1: Acoramidis (AG10) Group<br>Intervention/Treatment: Drug: acoramidis (AG10) 800 mg orally twice daily.<br>Participant Group/Arm 2: Placebo Group<br>Intervention/Treatment: Drug: Placebo Oral Tablet administered orally twice daily.<br><br>Primary Outcome Measures:<br>Outcome Measure: Change in 6-minute walk distance (6MWD)<br>Measure Description: The difference in the distance walked in 6 minutes from baseline to 12 months.<br>Time Frame: Baseline and 12 months.<br><br>Secondary Outcome Measures:<br>Outcome Measure: Change in NYHA class<br>Measure Description: Improvement or worsening of NYHA class from baseline to 12 months.<br>Time Frame: Baseline and 12 months.<br>Outcome Measure: Hospitalization due to heart failure<br>Measure Description: Number of hospitalizations due to heart failure.	revascularization, or experienced stroke or transient ischemic attack within 90 days prior to screening<br>Has hemodynamic instability<br>Likely to undergo heart transplantation within a year of screening<br>Confirmed diagnosis of primary (light chain) amyloidosis<br>Biomarkers of myocardial wall stress, NT-proBNP level ≥8500 pg/mL at screening<br>Measure of kidney function, eGFR by MDRD formula <15 mL/min/1.73 m2<br>Current treatment with marketed drug products and other investigational agents for the treatment of ATTR-CM<br>Current treatment with calcium channel blockers with conduction system effects (e.g. verapamil, diltiazem). The use of dihydropyridine calcium channel blockers is allowed.<br>The use of digitalis will only be allowed if required for management of atrial fibrillation with rapid ventricular response<br><br>Sex/Gender: All<br><br>Ages: 18 Years to 90 Years (Adult, Older Adult)<br><br>Enrollment size (Estimated): 510<br><br>Arms and Interventions:<br>Experimental: acoramidis HCl 800 mg<br>Subjects will receive acoramidis HCl 800 mg twice daily. 6MWT primary outcome will be assessed at the end of 12 months. The hierarchical combination of All-Cause mortality, cumulative frequency of cardiovascular-related hospitalizations, change from baseline in NT-proBNP levels, and change from baseline in distance walked on the 6MWT will be assessed after 30 months of treatment.<br>Interventions:<br>Drug: acoramidis<br>Placebo Comparator: Placebo<br>Subjects will receive placebo to match twice daily. 6MWT primary

		Time Frame: From baseline to 12 months.<br><br>Clinical Reasoning:<br><br>Inclusion Criteria:<br>Focused on adults with symptomatic ATTR-CM to ensure the study population is directly affected by the condition being studied.<br>Requirement for stable heart failure medication ensures that changes in outcomes can be more confidently attributed to the study intervention.<br><br>Exclusion Criteria:<br>Excluding individuals with severe renal impairment or liver disease minimizes the risk of adverse events that could confound the study results.<br>Exclusion of pregnant or breastfeeding women is standard to protect these populations from potential unknown risks.<br><br>Sex/Gender:<br>Including all genders ensures the findings are applicable to a broader population.<br><br>Ages:<br>Limiting the study to adults ensures the participants can provide informed consent and that the study focuses on the population most commonly affected by ATTR-CM.<br><br>Enrollment size:<br>A sample size of 400 is chosen to ensure adequate power to detect a significant difference between the	outcome will be assessed at the end of 12 months. The hierarchical combination of All-Cause mortality, cumulative frequency of cardiovascular-related hospitalizations, change from baseline in NT-proBNP levels, and change from baseline in distance walked on the 6MWT will be assessed after 30 months of treatment.<br>Interventions:<br>Drug: Placebo Oral Tablet<br><br>Primary Outcome Measures:<br>6-Minute Walk Test (6MWT) through Month 12 [Time Frame: 12 months]<br>Change from baseline to Month 12 of treatment in the total distance walked in 6 minutes<br>A hierarchical combination of all-cause mortality, cumulative frequency of cardiovascular-related hospitalization, change from baseline in NT-proBNP, and change from baseline in 6MWT over a 30-month fixed treatment duration [Time Frame: 30 months]<br>Each subject will be compared to every other subject within a stratum over outcomes of all-cause mortality (death due to any cause), cumulative frequency of cardiovascular-related hospitalizations (number of times a subject is hospitalized for cardiovascular-related causes), change from baseline in NT-proBNP, and change from baseline in the total distance walked in 6 minutes (distance in meters).<br>The hierarchical approach with the Finkelstein-Schoenfeld test will be applied and the test recognizes the greater importance of the mortality endpoint. Scores are transformed to -1, 0, +1. The alternative hypothesis is a subject in the acoramidis treatment group will have a greater score than a subject in the placebo group.<br><br>Secondary Outcome Measures:<br>Evaluate effects of acoramidis on quality of life (QoL) through Month 12 [Time Frame: 12 months]

		treatment and placebo groups, considering potential dropouts.<br><br>Arms and Interventions:<br>The parallel assignment allows for a direct comparison between the treatment and placebo groups.<br>The choice of intervention reflects the aim to test the efficacy and safety of acoramidis (AG10) in this specific patient population.<br><br>Primary and Secondary Outcome Measures:<br>The primary outcome measure of 6MWD is a well-recognized, objective measure of functional status in heart failure patients.<br>Secondary outcomes focus on clinical status and healthcare utilization, providing a comprehensive view of the treatment's impact.<br><br>This trial design aims to rigorously test the efficacy and safety of acoramidis (AG10) in improving outcomes for patients with ATTR-CM, using a well-justified participant selection and outcome measurement strategy.	Change from Baseline to Month 12 as measured in the Kansas City Cardiomyopathy Questionnaire Overall Summary score (KCCQ-OS). The KCCQ is a 23-item questionnaire developed to measure health status and health-related quality of life in subjects with heart failure. Items include heart failure symptoms, impact on physical and social functions, and how their heart failure impacts their quality of life (QoL). An Overall Summary score can be derived from the physical function, symptom (frequency and severity), social function and quality of life domains. For each domain, scores are transformed to a range of 0-100 using the formula, 100*[(mean of questions actually answered) - 1]/4, in which higher scores reflect better health status. The Overall Summary score is the mean of the domains scores, range from 0 to 100, in which higher scores reflect better health status.<br>Evaluate 6-Minute Walk Test (6MWT) through Month 30 [Time Frame: 30 months]<br>Change from baseline to Month 30 of treatment in the total distance walked in 6 minutes<br>Evaluate effects of acoramidis on quality of life (QoL) through Month 30 [Time Frame: 30 months]<br>Change from Baseline to Month 30 as measured in the Kansas City Cardiomyopathy Questionnaire Overall Summary score (KCCQ-OS). The KCCQ is a 23-item questionnaire developed to measure health status and health-related quality of life in subjects with heart failure. Items include heart failure symptoms, impact on physical and social functions, and how their heart failure impacts their quality of life (QoL). An Overall Summary score can be derived from the physical function, symptom (frequency and severity), social function and quality of life domains. For each domain, scores are transformed to a range of 0-100 using the formula, 100*[(mean of questions actually answered) - 1]/4,

			in which higher scores reflect better health status. The Overall Summary score is the mean of the domains scores, range from 0 to 100, in which higher scores reflect better health status.<br>Assess PD effects of circulating prealbumin by in vivo biomarker stabilization through Month 30 [Time Frame: 30 months]<br>Change from baseline to Month 30 in serum TTR (prealbumin) level (an in vivo measure of TTR stabilization)<br>Assess all-cause mortality [Time Frame: 30 months]<br>All-Cause Mortality by Month 30 including death due to any cause, heart transplant, or CMAD<br>Assess safety and tolerability through Month 12 [Time Frame: 12 months]<br>Safety parameters to be assessed: treatment- emergent serious adverse events (SAEs) and adverse events (AEs), AEs leading to treatment discontinuation, abnormal physical exam findings of clinical relevance, abnormal vital signs of clinical relevance, abnormal ECG parameters of clinical relevance, and changes in clinical safety laboratory parameters of potential clinical concern<br>PD assessments of TTR stabilization through Month 12 [Time Frame: 12 months]<br>Change from baseline in TTR (prealbumin) level (an in vivo measure of TTR stabilization) at Month 12<br>TTR stabilization as measured in established ex-vivo assays (fluorescent probe exclusion [FPE] and Western blot) at Month 12 in the PK-PD substudy<br>Efficacy by individual components and hierarchical combinations through Month 30 [Time Frame: 30 months]<br>A hierarchical combination of All-Cause mortality and cumulative frequency of CV-related hospitalization over a 30-month fixed treatment duration

			A hierarchical combination of All-Cause mortality, cumulative frequency of CV-related hospitalization, and change from baseline in 6MWT over a 30-month fixed treatment duration<br>Change in NT-proBNP from baseline to Month 30 of treatment<br>Cumulative frequency of CV-related hospitalization by Month 30<br>Efficacy of acoramidis in reducing CV mortality [Time Frame: 30 months]<br>Total number of deaths adjudicated as being related to cardiovascular causes<br>Incidence of treatment-emergent events [Time Frame: 30 months]<br>Assessment of incidence of treatment- emergent serious adverse events (SAEs) and adverse events (AEs)
5	Initial Trial Description<br>Official Title: Efficacy and Safety of Tirzepatide Once Weekly Versus Placebo for Maintenance of Weight Loss in Participants Without Type 2 Diabetes Who Have Obesity or Are Overweight With Weight-Related Comorbidities: A Randomized, Double-Blind, Placebo-Controlled Trial (SURMOUNT-4)<br><br>Brief Summary: This is a study of tirzepatide in participants with obesity or overweight. The main purpose is to learn more about how tirzepatide maintains body weight loss. The study has two phases: a lead-in phase in which all participants take	Inclusion Criteria:<br>Adults aged 18 years or older.<br>Body Mass Index (BMI) ≥30 kg/m^2 (obesity) or BMI ≥27 kg/m^2 (overweight) with at least one weight-related comorbidity (e.g., hypertension, dyslipidemia, obstructive sleep apnea).<br>Participants who have achieved a predefined percentage of weight loss during the lead-in phase with tirzepatide.<br>Ability to provide informed consent.<br><br>Exclusion Criteria:<br>Type 2 diabetes.<br>History of bariatric surgery or plans to undergo bariatric surgery during the study period.<br>Use of weight loss medications within 3 months prior to the start of the lead-in phase.<br>Serious psychiatric illness, including major depression or eating	Inclusion Criteria:<br>Body Mass Index (BMI) ≥30 kilograms per square meter (kg/m²), or ≥27 kg/m² and previous diagnosis with at least one of the following comorbidities: hypertension, dyslipidemia, obstructive sleep apnea, cardiovascular disease<br>History of at least one unsuccessful dietary effort to lose body weight<br><br>Exclusion Criteria:<br>Diabetes mellitus<br>Change in body weight greater than 5 kg within 3 months prior to starting study<br>Obesity induced by other endocrinologic disorders or monogenetic or syndromic forms of obesity<br>History of pancreatitis<br>Family or personal history of medullary thyroid carcinoma (MTC) or multiple endocrine neoplasia syndrome type 2 (MEN-2)<br>History of significant active or unstable major depressive disorder (MDD) or other severe psychiatric disorder within the last 2 years<br>Any lifetime history of a suicide attempt<br><br>Sex/Gender: All

	tirzepatide and a treatment phase in which participants will either continue tirzepatide or switch to placebo. The study will last about 2 years (25 visits).<br><br>Study Type: Interventional<br><br>Study Phase: Phase 3<br><br>Study Design:<br>Allocation: Randomized<br>Interventional Model: Parallel Assignment<br>Masking:Double (Participant Investigator)<br>Primary Purpose: Treatment<br><br>Conditions:<br>Obesity<br>Overweight<br><br>Intervention / Treatment:<br>Drug: Tirzepatide Administered SC<br>Other Names: LY3298176<br>Other: Placebo. Administered SC	disorders, that could interfere with participation.<br>Pregnant or breastfeeding women.<br><br>Sex/Gender: ALL<br><br>Ages: 18 years and older<br><br>Enrollment size (Estimated): 600<br><br>Arms and Interventions:<br>Participant Group/Arm 1: Continued Tirzepatide Treatment<br>Intervention/Treatment: Drug: Tirzepatide, Administered SC once weekly.<br>Participant Group/Arm 2: Placebo Group<br>Intervention/Treatment: Other: Placebo, Administered SC once weekly.<br><br>Primary Outcome Measures:<br>Outcome Measure: Percentage of participants maintaining ≥50% of the weight loss achieved during the lead-in phase.<br>Measure Description: This will assess the efficacy of tirzepatide in maintaining weight loss compared to placebo.<br>Time Frame: At 76 weeks (end of the treatment phase).<br><br>Secondary Outcome Measures:<br>Outcome Measure: Change in systolic and diastolic blood pressure from baseline to the end of the treatment phase.<br>Measure Description: This will evaluate the	Ages: 18 Years and older (Adult, Older Adult)<br><br>Enrollment size (Estimated): 750<br><br>Arms and Interventions:<br>Experimental: Tirzepatide<br>Tirzepatide administered subcutaneously (SC)<br>Interventions:<br>Drug: Tirzepatide<br>Placebo Comparator: Placebo<br>Placebo administered SC<br>Interventions:<br>Other: Placebo<br><br>Primary Outcome Measures:<br>Percent Change from Randomization (Week 36) in Body Weight [Time Frame: Randomization, Week 88]<br>Percent change from randomization in body weight<br><br>Secondary Outcome Measures:<br>Change from Randomization in Body Weight [Time Frame: Randomization, Week 88]<br>Change from randomization in body weight<br>Change from Randomization in Waist Circumference [Time Frame: Randomization, Week 88]<br>Change from randomization in waist circumference<br>Percentage of Participants Who Maintain ≥80% of the Body Weight Lost During the Open-Label Period [Time Frame: Week 88]<br>Percentage of participants who maintain ≥80% of the body weight lost during the open-label period<br>Percentage of Participants Who Achieve ≥5% Body Weight Reduction [Time Frame: Week 88]<br>Percentage of participants who achieve ≥5% body weight reduction<br>Percentage of Participants Who Achieve ≥10% Body Weight Reduction [Time Frame: Week 88]<br>Percentage of participants who achieve ≥10% body weight reduction<br>Time to First Occurrence of Participants Returning to >95% Baseline Weight for Those Who Lost ≥5% during the Open-Label Period

		impact of weight maintenance on blood pressure.<br>Time Frame: Baseline and 76 weeks.<br>Outcome Measure: Change in lipid profile (HDL, LDL, total cholesterol, triglycerides) from baseline to the end of the treatment phase.<br>Measure Description: This will assess the effect of weight maintenance on lipid metabolism.<br>Time Frame: Baseline and 76 weeks.<br><br>Clinical Reasoning:<br><br>Inclusion Criteria: Focused on adults with obesity or overweight with comorbidities to target the population most likely to benefit from weight maintenance interventions. The requirement for a predefined weight loss during the lead-in phase ensures participants respond to tirzepatide.<br>Exclusion Criteria: Excluding individuals with type 2 diabetes focuses the study on weight management without the confounding effects of diabetes treatment. Excluding recent use of weight loss medications and those with psychiatric illnesses ensures the effects observed are attributable to tirzepatide.<br>Sex/Gender: Including all genders ensures the findings are generalizable across the population.	[Time Frame: Randomization, Week 88]<br>Time to first occurrence of participants returning to >95% baseline weight for those who lost ≥5% during the open-label period<br>Percent Change from Randomization in Body Weight [Time Frame: Randomization, Week 64]<br>Percent change from randomization in body weight<br>Change from Randomization in Body Mass Index (BMI) [Time Frame: Randomization, Week 88]<br>Change from randomization in BMI<br>Change from Randomization in Fasting Glucose [Time Frame: Randomization, Week 88]<br>Change from randomization in fasting glucose<br>Change from Randomization in Hemoglobin A1c (HbA1c) [Time Frame: Randomization, Week 88]<br>Change from randomization in HbA1c<br>Change from Randomization in Fasting Insulin [Time Frame: Randomization, Week 88]<br>Change from randomization in fasting insulin<br>Change from Randomization in Total Cholesterol [Time Frame: Randomization, Week 88]<br>Change from randomization in total cholesterol<br>Change from Randomization in Low Density Lipoprotein (LDL) Cholesterol [Time Frame: Randomization, Week 88]<br>Change from randomization in LDL cholesterol<br>Change from Randomization in High Density Lipoprotein (HDL) Cholesterol [Time Frame: Randomization, Week 88]<br>Change from randomization in HDL cholesterol<br>Change from Randomization in Very Low Density Lipoprotein (VLDL) Cholesterol [Time Frame: Randomization, Week 88]<br>Change from randomization in VLDL cholesterol<br>Change from Randomization in Triglycerides [Time Frame: Randomization, Week 88]

		Ages: Limiting the study to adults ensures the safety and efficacy data are relevant to the population most affected by obesity and overweight.<br>Enrollment size: Chosen to ensure statistical power to detect meaningful differences while considering practicality and resource constraints.<br>Arms and Interventions: A direct comparison between continued tirzepatide use and placebo after a lead-in phase allows for a clear assessment of tirzepatide's efficacy in weight maintenance.<br>Primary and Secondary Outcome Measures: Selected to directly measure the primary goal of weight maintenance and its impact on cardiovascular risk factors, providing a comprehensive assessment of tirzepatide's benefits and safety.	Change from randomization in triglycerides<br>Change from Randomization in Free Fatty Acids [Time Frame: Randomization, Week 88]<br>Change from randomization in free fatty acids<br>Change from Randomization in Systolic Blood Pressure (SBP) [Time Frame: Randomization, Week 88]<br>Change from randomization in SBP<br>Change from Randomization in Diastolic Blood Pressure (DBP) [Time Frame: Randomization, Week 88]<br>Change from randomization in DBP<br>Change from Randomization in Short Form 36 Version 2 Health Survey (SF 36v2) Acute Form Physical Functioning Domain Score [Time Frame: Randomization, Week 88]<br>The SF-36v2 acute form, 1-week recall assesses participants' health-related quality of life (HRQoL) on 8 domains; 1) limitations in physical functioning due to health problems; 2) limitations in usual role because of physical health problems; 3) bodily pain; 4) general health perceptions; 5) vitality; 6) limitations in social functioning 7) limitations in usual role due to emotional problems; and 8) general mental health. Domain scores are norm-based and presented in the form of T-scores, with a mean of 50 and standard deviation of 10, with higher scores indicating better levels of function and/or better health.<br>Change from Randomization in Impact of Weight on Quality of Life Lite Clinical Trials Version (IWQOL-Lite-CT) Physical Function Composite Score [Time Frame: Randomization, Week 88]<br>The IWQOL Lite-CT consists of 20 items, assessing 2 primary domains of obesity related HRQoL: Physical (7 items) and Psychosocial (13 items). A 5 item subset of the Physical domain - the Physical Function composite - is also supported. Items in the Physical Function composite describe physical impacts related to general and specific physical activities. Individual composite scale

			scores and a total score can be computed and are presented on a scale of 0 to 100, with higher scores indicating better function.<br>Change from Baseline in Body Weight [Time Frame: Baseline, Week 88]<br>Change from baseline in body weight<br>Percent Change from Baseline in Body Weight [Time Frame: Baseline, Week 88]<br>Percent change from baseline in body weight<br>Change from Baseline in BMI [Time Frame: Baseline, Week 88]<br>Change from baseline in BMI<br>Percentage of Participants Who Achieve ≥15% Body Weight Reduction [Time Frame: Week 88]<br>Percentage of participants who achieve ≥15% body weight reduction<br>Change from Baseline in Waist Circumference [Time Frame: Baseline, Week 88]<br>Change from baseline in waist circumference<br>Change from Baseline in Fasting Glucose [Time Frame: Baseline, Week 88]<br>Change from baseline in fasting glucose<br>Change from Baseline in HbA1c [Time Frame: Baseline, Week 88]<br>Change from baseline in HbA1c<br>Change from Baseline in Fasting Insulin [Time Frame: Baseline, Week 88]<br>Change from baseline in fasting insulin<br>Change from Baseline in Total Cholesterol [Time Frame: Baseline, Week 88]<br>Change from baseline in total cholesterol<br>Change from Baseline in HDL Cholesterol [Time Frame: Baseline, Week 88]<br>Change from baseline in HDL cholesterol<br>Change from Baseline in LDL Cholesterol [Time Frame: Baseline, Week 88]<br>Change from baseline in LDL cholesterol

			Change from Baseline in VLDL Cholesterol [Time Frame: Baseline, Week 88]<br>Change from baseline in VLDL cholesterol<br>Change from Baseline in Triglycerides [Time Frame: Baseline, Week 88]<br>Change from baseline in triglycerides<br>Change from Baseline in Free Fatty Acids [Time Frame: Baseline, Week 88]<br>Change from baseline in free fatty acids<br>Change from Baseline in SBP [Time Frame: Baseline, Week 88]<br>Change from baseline in SBP<br>Change from Baseline in DBP [Time Frame: Baseline, Week 88]<br>Change from baseline in DBP<br>Change from Randomization in SF 36v2 Acute Form Physical Functioning Domain Score [Time Frame: Baseline, Week 88]<br>The SF-36v2 acute form, 1-week recall assesses participants' HRQoL on 8 domains; 1) limitations in physical functioning due to health problems; 2) limitations in usual role because of physical health problems; 3) bodily pain; 4) general health perceptions; 5) vitality; 6) limitations in social functioning 7) limitations in usual role due to emotional problems; and 8) general mental health. Domain scores are norm-based and presented in the form of T-scores, with a mean of 50 and standard deviation of 10, with higher scores indicating better levels of function and/or better health.<br>Change from Baseline in IWQOL-Lite-CT Physical Function Composite Score [Time Frame: Baseline, Week 88]<br>The IWQOL Lite-CT consists of 20 items, assessing 2 primary domains of obesity related HRQoL: Physical (7 items) and Psychosocial (13 items). A 5 item subset of the Physical domain - the Physical Function composite - is also supported. Items in the Physical Function composite describe physical impacts related to general and specific physical

			activities. Individual composite scale scores and a total score can be computed and are presented on a scale of 0 to 100, with higher scores indicating better function.
6	Initial Trial Description<br>Official Title: A Phase III Randomized, Double-Blind, Controlled Trial of the Clinical Efficacy of Typhoid Conjugate Vaccine (Vi-TCV) Among Children Age 9 Months Through 12 Years in Blantyre, Malawi<br><br>Brief Summary:<br>This study will evaluate the efficacy of a Typhoid conjugate vaccine (Vi-TCV) in Malawi, Africa among children age 9 months through 12 years. Participants will be randomized in a 1:1 ration to receive the study vaccine or the control vaccine (meningococcal group A conjugate vaccine - MCV-A).<br><br>Study Type: Interventional<br><br>Study Phase: Phase 3<br><br>Study Design:<br>Allocation: Randomized<br>Interventional Model: Parallel Assignment<br>Masking: Triple (Participant Investigator Outcomes Assessor)<br>Primary Purpose: Prevention<br><br>Conditions:	Inclusion Criteria:<br>Children aged 9 months to 12 years.<br>Resident of the study area in Blantyre, Malawi.<br>Parent or guardian willing to provide informed consent.<br>Available for follow-up for the duration of the study period.<br><br>Exclusion Criteria:<br>History of allergic reaction to any component of the vaccines.<br>Receipt of any vaccine within the 4 weeks prior to the study enrollment.<br>Any confirmed or suspected immunosuppressive or immunodeficient condition.<br>Acute disease or fever (>38.5°C) at the time of enrollment.<br>Previous vaccination with any typhoid or meningococcal vaccine.<br><br>Sex/Gender: ALL<br><br>Ages: 9 months to 12 years<br><br>Enrollment size (Estimated): 10,000<br><br>Arms and Interventions:<br>Participant Group/Arm 1: Typhoid Conjugate Vaccine (Vi-TCV) group<br>Intervention/Treatment: Biological: Vi-Typhoid Conjugate Vaccine (Vi-TCV), Single 0.5-ml intramuscular injection.	Inclusion Criteria:<br>Healthy male or female child between the ages of 9 months and 12 years/364 days at the time of study vaccination.<br>A child whose parent or guardian resides primarily within the Ndirande or Zingwangwa study areas at the time of study vaccinations and who intends to be present in the area for the duration of the trial.<br>A child whose parent or guardian has voluntarily given informed consent.<br><br>Exclusion Criteria:<br>History of documented hypersensitivity to any component of the vaccine<br>Prior receipt of any typhoid vaccine in the past 3 years<br>History of severe allergic reaction with generalized urticarial, angioedema, or anaphylaxis<br>Any condition determined by the investigator to be likely to interfere with evaluation of the vaccine or to be a significant potential health risk to the child or make it unlikely that the child would complete the study.<br>Temporary Exclusion Criteria:<br>The following will be considered temporary contraindications to enrollment and vaccination. If these apply, the participant will be temporarily excluded for vaccination until 48 hours has passed. A re-assessment will be needed to ensure these temporary exclusion criteria no longer exist.<br>Reported fever within 24 hours prior to vaccination<br>Use of anti-pyretics within 4 hours prior to vaccination<br>An additional temporary exclusion criteria will be:<br>Receipt of measles vaccine in the one month prior to enrollment, as determined by parental history or vaccination card.

	Typhoid<br><br>Intervention / Treatment: Biological: Vi-Typhoid Conjugate Vaccine (Vi-TCV)<br>Single 0.5-ml intramuscular injection<br>Biological: Meningococcal A Conjugate Vaccine (MCV-A)<br>Single intramuscular injection. Children 9-11 months will receive a 5µg/0.5ml dose. Children 12 months and older will receive a 10µg/0.5 ml dose.	Participant Group/Arm 2: Control group (Meningococcal A Conjugate Vaccine - MCV-A)<br>Intervention/Treatment: Biological: Meningococcal A Conjugate Vaccine (MCV-A), Single intramuscular injection. Dose adjusted by age as described.<br><br>Primary Outcome Measures:<br>Outcome Measure: Incidence of clinically confirmed typhoid fever.<br>Measure Description: Number of participants with blood culture-confirmed typhoid fever.<br>Time Frame: From the date of vaccination up to 24 months post-vaccination.<br><br>Secondary Outcome Measures:<br>Outcome Measure: Incidence of adverse events following immunization (AEFI).<br>Measure Description: Number of participants experiencing adverse events categorized by severity within 28 days post-vaccination.<br>Time Frame: 28 days post-vaccination.<br>Outcome Measure: Seroprotection rate.<br>Measure Description: Proportion of participants achieving a predefined antibody titer considered protective against typhoid fever.<br>Time Frame: 6 months post-vaccination.<br><br>Clinical Reasoning:	Additional Exclusion Criteria for Safety and Immunogenicity Substudy:<br>In addition to the exclusion criteria of the efficacy study, participants enrolled in the immunogenicity and reactogenicity substudy may not have, or have had, any:<br>Known history of diabetes, tuberculosis, cancer, chronic kidney, heart, or liver disease, progressive neurological disorders, poorly controlled seizures, or terminal illness<br>Severe malnutrition as determined by MUAC< 12.5 cm for children younger than 5 years;<br>Receipt of any other investigational intervention in the last 6 months or anticipated during the course of the study.<br>Receipt of blood products in the last 6 months.<br>Known HIV-infection or exposure or other immunosuppressive conditions.<br>Receipt of systemic immunosuppressant or systemic corticosteroids.<br>Receipt of any measles-containing vaccine for children younger than 1 year of age<br><br>Sex/Gender: All<br><br>Ages: 9 Months to 12 Years (Child)<br><br>Enrollment size (Estimated): 24000<br><br>Arms and Interventions:<br>Experimental: Vi-Typhoid Conjugate Vaccine (Vi-TCV)<br>Children will receive a single 0.5-ml dose of Vi-TCV administered by the intramuscular route.<br>Interventions:<br>Biological: Vi-Typhoid Conjugate Vaccine (Vi-TCV)<br>Active Comparator: Meningococcal A Conjugate Vaccine (MCV-A)<br>Children will receive a single dose of MCV-A administered by the intramuscular route. Children 9-11 months will receive a 5µg/0.5ml dose. Children 12 months and older will receive a 10µg/0.5 ml dose.<br>Interventions:

		Inclusion Criteria: Targeting children aged 9 months to 12 years ensures the vaccine's efficacy is tested across a broad age range of children who are at risk of typhoid in Malawi. Including only residents ensures the results are relevant to the local population.<br>Exclusion Criteria: Excluding children with a history of allergic reactions, recent vaccinations, or immunocompromising conditions ensures the safety of the participants and the integrity of the results. Excluding those with acute diseases reduces confounding variables.<br>Sex/Gender: Including all genders ensures the results are generalizable across the population.<br>Ages: The chosen age range is based on the high incidence of typhoid in this demographic in Malawi.<br>Enrollment size: A large sample size ensures the study has adequate power to detect differences in vaccine efficacy and safety.<br>Arms and Interventions: The choice of a control vaccine (MCV-A) rather than a placebo is ethical, providing participants in the control group with protection against another relevant disease. It also helps maintain blinding.<br>Primary Outcome Measures: Focusing on clinically confirmed typhoid fever ensures the study measures the	Biological: Meningococcal A Conjugate Vaccine (MCV-A)<br><br>Primary Outcome Measures:<br>Efficacy of Vi-TCV [Time Frame: Up to 36 months]<br>The primary outcome of interest is the incidence of blood culture-positive typhoid fever among Vi-TCV and MCV-A vaccine recipients in the study population during the entire post-vaccination surveillance period. Vaccine efficacy will be calculated as one minus the relative rate of symptomatic typhoid fever in the Vi-TCV group compared to that in the MCV-A group.<br><br>Secondary Outcome Measures:<br>Safety of Vi-TCV [Time Frame: 6 months]<br>The safety profile of Vi-TCV will be measured by comparing the proportions of participants experiencing solicited and unsolicited local and systemic reactions between children receiving Vi-TCV as compared to children receiving MCV-A according to the following three categories:<br>The proportion of participants who develop adverse events detected in the first 30 minutes after vaccination and for 7 days after vaccination.<br>The proportion of participants who experience serious adverse events within 6 months of vaccination in a subset of participants.<br>The proportion of participants who experience other non-serious adverse events up to 28 days following vaccination, in a subset of participants.<br>Immunogenicity of Vi-TCV [Time Frame: 28 days]<br>The immunogenicity of Vi-TCV in a subset of 600 participants measured by ELISA for anti-Vi percent seroconversion and GMTs before and at day 28 after vaccination.<br>Number of typhoid fever cases prevented Vi-TCV [Time Frame: Up to 36 months]<br>The number of blood culture-confirmed cases of typhoid fever

		vaccine's impact on preventing disease rather than just immunogenicity.<br>Secondary Outcome Measures: Monitoring adverse events and seroprotection rates provides comprehensive safety data and additional efficacy data, respectively.<br><br>This design aims to robustly test the efficacy and safety of the Vi-TCV in a high-risk population, providing critical data for public health decision-making in Malawi and similar settings.	prevented by Vi-TCV during the study period measured by comparing the incidence of blood culture-confirmed typhoid fever in participants receiving Vi-TCV compared to those receiving MCV-A.
7	Initial Trial Description<br>Official Title: Chemoprevention With Monthly IPTp With Dihydroartemisinin-piperaquine for Malaria in HIV-infected Pregnant Participants on Daily Cotrimoxazole in Kenya and Malawi: a Multi-centre Placebo-controlled Trial<br><br>Brief Summary: Short technical protocol summary<br>Background: Pregnant women represent a vulnerable population for malaria. HIV-infected women are particularly at risk. In HIV-infected pregnant women, WHO recommends daily cotrimoxazole (CTX), an antifolate drug, for malaria chemoprevention and prophylaxis against	Inclusion Criteria:<br>HIV-infected pregnant women.<br>Age 18 years or older.<br>In the 2nd or 3rd trimester of pregnancy at the time of enrollment.<br>Currently on DTG-based combination antiretroviral therapy (cARTs).<br>Willing to give informed consent.<br><br>Exclusion Criteria:<br>Known allergy to dihydroartemisinin-piperaquine (DP) or cotrimoxazole (CTX).<br>Severe or complicated malaria at the time of enrollment.<br>Any condition that, in the opinion of the investigators, might interfere with the trial results or participant's safety.<br>Participation in another clinical trial that could interfere with the outcomes of this trial.<br>Known cardiac conditions that could be	Inclusion Criteria:<br>HIV-infected pregnant women between 16-28 weeks' gestation<br>Viable singleton pregnancy<br>On or eligible for cARTs and CTX<br>A resident of the study area<br>Willing to adhere to scheduled and unscheduled study visit procedures<br>Willing to deliver in a study clinic or hospital<br>Provide written informed consent<br><br>Exclusion Criteria:<br>Multiple pregnancies (i.e. twin/triplets)<br>HIV-negative or HIV status unknown<br>Known heart ailment<br>Severe malformations or non-viable pregnancy if observed by ultrasound<br>Participants with advanced HIV-disease at WHO clinical stage 3 and 4<br>Confirmed or suspected TB infection,<br>Unable to give consent<br>Known allergy or contraindication to any of the study drugs<br><br>Sex/Gender: Pregnant women<br><br>Ages: (Child, Adult, Older Adult)<br><br>Enrollment size (Estimated): 898<br><br>Arms and Interventions:

	opportunistic infection. However, there is cross-resistance with sulphadoxine-pyrimethamine (SP), and high levels of antifolate resistance threatens the antimalarial effect of CTX. Recent trials with intermittent preventive therapy (IPT) with mefloquine in HIV-infected women on daily CTX, suggested that chemoprevention with an effective antimalarial markedly improves the protection against malaria compared to daily CTX alone. However, mefloquine was not well tolerated. The long-acting combination of dihydroartemisinin-piperaquine (DP) is well tolerated and has shown great promise as IPTp in HIV-negative women in East-Africa. Chemoprevention with monthly DP has also been explored in HIV-infected pregnant women on daily CTX in Uganda. Unfortunately, the study was inconclusive because malaria transmission was too low and a clinically relevant drug interaction with efavirenz (EFV) was found reducing the exposure to DP. WHO now recommends dolutegravir (DTG) based combination	exacerbated by either the study drug or the disease under study.<br><br>Sex/Gender: Pregnant women<br><br>Ages: 18 years and older<br><br>Enrollment size (Estimated): 898<br><br>Arms and Interventions: Intervention Arm: HIV-infected pregnant women on daily CTX and DTG-based cARTs receiving monthly intermittent preventive therapy with dihydroartemisinin-piperaquine (DP). Control Arm: HIV-infected pregnant women on daily CTX and DTG-based cARTs receiving a monthly placebo.<br><br>Primary Outcome Measures:<br>Cumulative incidence of malaria infection; The proportion of participants who develop malaria during the pregnancy period; Time Frame: From enrollment until delivery (up to 6 months).<br><br>Secondary Outcome Measures:<br>Adverse effects of malaria; Incidence of adverse effects including anemia and low birth weight; Time Frame: From enrollment until 6-8 weeks post-delivery. Pharmacokinetic assessment of DP; Measurement of DP levels in blood to assess	Placebo Comparator: CTX-alone<br>Daily, one double-strength tablet of 160mg of sulfamethoxazole and 800mg of trimethoprim plus monthly placebo-DP, given as a fixed dose of 3 placebo-DP tablets daily for three days until delivery.<br>Interventions:<br>Drug: Intermittent Preventive Therapy with Dihydroartemisinin-Piperaquine<br>Experimental: CTX-DP<br>Daily, one double-strength tablet of 160mg of sulfamethoxazole and 800mg of trimethoprim plus monthly DP, given as a fixed dose of 3 tablets (40 mg of dihydroartemisinin and 320 mg of piperaquine) daily for three days until delivery.<br>Interventions:<br>Drug: Intermittent Preventive Therapy with Dihydroartemisinin-Piperaquine<br><br>Primary Outcome Measures:<br>Cumulative incidence of malaria infection [Time Frame: Detected from 2 weeks after the first day of the first dose of the first course to delivery inclusive. The total duration of the trial is 24 months. Actual participant recruitment and follow up is expected to take up to 19 months.]<br>The primary outcome will be the cumulative incidence of malaria infection detected from 2 weeks after the first day of the first dose of the first course to delivery inclusive, defined as the presence of peripheral (maternal) or placental (maternal) Plasmodium infection detected by either molecular diagnostics (henceforth referred to as PCR), microscopy, RDT or placental histology (active infection).<br><br>Secondary Outcome Measures:<br>Efficacy of the intervention on the following listed secondary outcomes [Time Frame: The total duration of the trial is 24 months. Actual participant recruitment and follow up is expected to take up to 19 months (12 months recruitment plus 7 months of mother-infant follow-up until the child is 6 weeks old]<br>Incidence of malaria infection

	antiretroviral therapy (ARTs) as the preferred firstline regimen including for pregnant women in the 2nd and 3rd trimester of pregnancy for the prevention of mother-to-child transmission of HIV. As a result, many countries in Africa are now transitioning to DTG-based combination antiretroviral therapy (cARTs). No such drug-drug interaction is expected between DTG and DP. We will, therefore, assess the safety and efficacy of malaria chemoprevention with monthly DP in HIV-infected women on daily CTX and DTG-based cARTs.<br>Objectives and methods: This is a 2-arm, individually-randomized, multi-centre, placebo-controlled superiority trial comparing the safety and efficacy of daily CTX plus monthly DP ('CTX-DP') versus daily CTX plus monthly placebo-DP (i.e. 'CTX-alone', control arm) to reduce malaria and the adverse effects of malaria in 898 (449 per arm) HIV-infected pregnant women on DTG-based cARTs. The study will be conducted in 8 hospitals in Kenya and Malawi in high SP-resistance areas with a high prevalence of	drug exposure; Time Frame: At delivery.<br>Cardiac monitoring for safety; Assessment of QT interval to monitor cardiac safety; Time Frame: At enrollment, mid-pregnancy, and delivery.<br>Impact on immune responses to malaria; Measurement of malaria-specific immune responses; Time Frame: At delivery and 6-8 weeks post-delivery.<br><br>Clinical Reasoning:<br><br>Inclusion Criteria: Focused on HIV-infected pregnant women to address the specific risk this population faces regarding malaria, while ensuring they are on DTG-based cARTs due to the lack of expected drug-drug interaction with DP.<br><br>Exclusion Criteria: Aimed at ensuring participant safety by excluding those with conditions that could be exacerbated by the trial interventions or outcomes.<br><br>Sex/Gender: Limited to females due to the nature of pregnancy.<br><br>Ages: Set at 18 years and older to ensure participants can legally consent to participation.<br><br>Enrollment size: Calculated to ensure the trial is powered to detect a significant difference in the primary outcome,	The individual components of the composite malaria infection endpoints<br>Incidence of clinical malaria.<br>Malaria infection at delivery<br>Placental malaria by histology (active, past, and active and past infections pooled)<br>Placental malaria by any measure<br>Maternal peripheral malaria infection at delivery by any measure<br>Placental inflammation or chorioamnionitis<br>Adverse pregnancy outcome: the composite of foetal loss (spontaneous abortion or stillbirth), or singleton live births born small-for-gestational-age (SGA), or with low birthweight (LBW), or preterm (PT) (SGA-LBW-PT), or subsequent neonatal death by day 28.<br>Composite of foetal loss and neonatal mortality.<br>SGA-LBW-PT composite.<br>The individual components of the above composites<br>Neonatal length and stunting.<br>Evidence of arboviral infections<br>Safety: Cardiac safety, serious adverse events and MTCT of HIV [Time Frame: The total duration of the trial is 24 months. Actual participant recruitment and follow up is expected to take up to 19 months (12 months recruitment plus 7 months of mother-infant follow-up until the child is 6 weeks old]<br>QTc-prolongation.<br>Congenital malformations.<br>Maternal mortality<br>Other SAEs and AEs.<br>Mother to child transmission of HIV<br>Tolerance [Time Frame: The total duration of the trial is 24 months. Actual participant recruitment and follow up is expected to take up to 19 months (12 months recruitment plus 7 months of mother-infant follow-up until the child is 6 weeks old]<br>History of vomiting study drug (<30 min).<br>Dizziness.<br>Gastrointestinal complaints.<br>Antimicrobial activity and resistance [Time Frame: The total duration of

	malaria. These are the same sites where the sister trial in HIV-uninfected women is being conducted in Kenya and Malawi (IMPROVE trial). Both the mother and baby will be followed for 6-8 weeks after delivery. The study is powered at 80% (alpha=0.05) to detect ≥50% relative risk reduction (RR=0.50) in the primary outcome (cumulative incidence of malaria infection) from 12% in the CTX-alone arm (control arm) to 6% in in the interventions arm allowing for 20% non-contributors. The trial includes a pharmacokinetic assessment, cardiac monitoring for safety, assessment of antimalarial drug and the impact on immune responses to malaria<br><br>Study Type: Interventional<br><br>Study Phase: Phase 3<br><br>Study Design: Allocation: Randomized Interventional Model: Parallel Assignment Interventional Model Description: Allocation: randomized; intervention model: parallel assignment; arms: 2; allocation ratio: 1:1; stratified by site (hospital) and HIV-status (known-	allowing for a 20% dropout rate.<br><br>Arms and Interventions: Designed to compare the efficacy and safety of DP as IPTp against a control group receiving placebo, while all participants continue their standard care including CTX and cARTs.<br><br>Primary and Secondary Outcome Measures: Selected to comprehensively assess the impact of DP on malaria prevention, safety, drug interactions, and immune response, with appropriate time frames for each measure to capture relevant data.<br><br>This trial design aims to address the critical need for effective malaria chemoprevention in HIV-infected pregnant women, considering the limitations of current recommendations and the potential of DP in this context.	the trial is 24 months. Actual participant recruitment and follow up is expected to take up to 19 months (12 months recruitment plus 7 months of mother-infant follow-up until the child is 6 weeks old]<br>Frequency of molecular markers of drug resistance in Plasmodium falciparum infections during pregnancy and delivery.<br>Pharmacokinetic parameters [Time Frame: The total duration of the trial is 24 months. Actual participant recruitment and follow up is expected to take up to 19 months (12 months recruitment plus 7 months of mother-infant follow-up until the child is 6 weeks old]<br>Standard pharmacokinetic parameters for dolutegravir, piperaquine and CTX.

	positive and newly-diagnosed). Masking: Placebo controlled Masking: Quadruple (Participant Care Provider Investigator Outcomes Assessor) Primary Purpose: Prevention<br><br>Conditions: Pregnancy; HIV; Malaria<br><br>Intervention / Treatment:<br>Drug: Intermittent Preventive Therapy with Dihydroartemisinin-Piperaquine. Monthly DP fixed dose of 3 tablets (40 mg of dihydroartemisinin and 320 mg of piperaquine) daily for three days until delivery. All participants will (continue to) receive daily cotrimoxazole (CTX) (one double-strength tablet of 160mg of sulfamethoxazole and 800mg of trimethoprim) and anti-retroviral drugs. Other Names: Monthly DP D-artepp		
8	Initial Trial Description<br>Official Title: A Multi-center, Randomized, Double-blind, Active and Placebo-controlled Study to Investigate the Safety and Efficacy of Ligelizumab (QGE031) in the Treatment of Chronic Spontaneous Urticaria (CSU) in Adolescents and	Inclusion Criteria:<br>Male and female subjects aged ≥ 12 years.<br>Diagnosed with Chronic Spontaneous Urticaria (CSU) for at least 6 months prior to screening.<br>Subjects who remain symptomatic despite the use of H1-antihistamines at approved doses for at least 2 months.	Inclusion Criteria:<br>Signed informed consent must be obtained prior to participation in the study. The subject's, parent's or legal guardian's signed written informed consent and child's assent, if appropriate, must be obtained before any assessment is performed. Of note, if the subject reaches age of consent (age as per local law) during the study, they will also need to sign the corresponding study Informed Consent Form (ICF) at the next study visit.

	Adults Inadequately Controlled With H1-antihistamines<br><br>Brief Summary: The purpose of this study was to establish safety and efficacy of ligelizumab in adolescent and adult subjects with Chronic Spontaneous Urticaria (CSU) who remain symptomatic despite standard of care treatment by demonstrating better efficacy over omalizumab and over placebo. The study population consisted of 1,072 male and female subjects aged ≥ 12 years who were diagnosed with CSU and who remained symptomatic despite the use of H1-antihistamines.This was a multi-center, randomized, double-blind, active- and placebo-controlled, parallel-group study. There was a screening period of up to 28 days, a 52 week double-blind treatment period, and a 12 week post-treatment follow-up period.<br><br>Study Type: Interventional<br><br>Study Phase: Phase 3<br><br>Study Design: Allocation: Randomized Interventional Model: Parallel Assignment	Ability to provide informed consent (subjects under the age of consent must have a guardian provide consent and assent from the subject).<br><br>Exclusion Criteria:<br>Subjects with urticarial vasculitis or other forms of urticaria (e.g., physical urticaria).<br>Previous treatment with ligelizumab or omalizumab.<br>Use of systemic corticosteroids or immunosuppressive drugs within 4 weeks prior to screening.<br>Presence of any significant co-morbid condition that, in the investigator's opinion, would interfere with study participation or evaluation of results.<br>Pregnant or breastfeeding women.<br><br>Sex/Gender: ALL<br><br>Ages: ≥ 12 years<br><br>Enrollment size (Estimated): 1,072<br><br>Arms and Interventions:<br>Participant Group/Arm 1: Ligelizumab<br>Intervention/Treatment: Biological: Ligelizumab, liquid in vial, administered subcutaneously.<br>Participant Group/Arm 2: Omalizumab<br>Intervention/Treatment: Biological: Omalizumab, lyophilized powder for solution in vial, administered subcutaneously.	Male and female subjects ≥ 12 years of age at the time of screening.<br>CSU diagnosis for ≥ 6 months.<br>Diagnosis of CSU refractory to H1-AH at approved doses at the time of randomization, as defined by all of the following:<br>The presence of itch and hives for ≥ 6 consecutive weeks at any time prior to Visit 1 (Day - 28 to Day -14) despite current use of non-sedating H1-antihistamine<br>UAS7 score (range 0-42) ≥ 16 and HSS7 (range 0-21) ≥ 8 during the 7 days prior to randomization (Visit 110, Day 1)<br>Subjects must be on H1-antihistamine at only locally label approved doses for treatment of CSU starting at Visit 1 (Day -28 to Day -14)<br>Willing and able to complete a daily symptom eDiary for the duration of the study and adhere to the study visit schedules.<br><br>Exclusion Criteria:<br>History of hypersensitivity to any of the study drugs or their excipients or to drugs of similar chemical classes (i.e. to murine, chimeric or human antibodies).<br>Subjects having a clearly defined cause of their chronic urticaria, other than CSU. This includes, but is not limited to, the following: symptomatic dermographism (urticaria factitia), cold-, heat-, solar-, pressure-, delayed pressure-, aquagenic-, cholinergic- or contact-urticaria.<br>Diseases, other than chronic urticaria, with urticarial or angioedema symptoms such as urticarial vasculitis, erythema multiforme, cutaneous mastocytosis (urticaria pigmentosa) and hereditary or acquired angioedema (eg, due to C1 inhibitor deficiency).<br>Subjects with evidence of helminthic parasitic infection as evidenced by stools being positive for a pathogenic organism according to local guidelines. All subjects will be screened at Visit 1. If stool testing is positive for pathogenic organism, the

	Interventional Model Description: This was a Phase III multi-center, randomized, double-blind, active- and placebo-controlled, parallel-group study. There was a screening period of up to 28 days, a 52 week double-blind treatment period, and a 12 week post-treatment follow-up period.<br>Masking: Triple (Participant Care Provider Investigator)<br>Masking Description: Patients, investigator staff and personnel performing the study assessments remained blinded to the identity of the treatment from the time of randomization until final database lock. The study drug was prepared by an independent unblinded pharmacist (or authorized delegate) and administered by an independent unblinded study drug administrator. Neither the unblinded pharmacist nor the unblinded study drug administrator was involved in any assessments.<br>Primary Purpose: Treatment<br><br>Conditions: Chronic Spontaneous Urticaria<br><br>Intervention / Treatment:	Participant Group/Arm 3: Placebo<br>Intervention/Treatment: Other: Placebo, liquid in vial, administered subcutaneously.<br><br>Primary Outcome Measures:<br>Outcome Measure: Change in the Urticaria Activity Score over 7 days (UAS7) from baseline to Week 12.<br>Measure Description: The UAS7 is a composite score assessing the number of hives and itch severity over 7 days.<br>Time Frame: Baseline to Week 12.<br><br>Secondary Outcome Measures:<br>Outcome Measure: Proportion of subjects with complete response (UAS7=0) at Week 24.<br>Measure Description: Assessment of subjects achieving no hives and no itch.<br>Time Frame: At Week 24.<br>Outcome Measure: Change in quality of life from baseline to Week 24 as measured by the Dermatology Life Quality Index (DLQI) or Children's Dermatology Life Quality Index (CDLQI).<br>Measure Description: Assessment of the impact of CSU on subjects' life quality.<br>Time Frame: Baseline to Week 24.<br><br>Clinical Reasoning:<br><br>Inclusion Criteria: Selected to ensure	subject will not be randomized and will not be allowed to rescreen.<br>Any other skin disease associated with chronic itching that might influence in the investigators opinion the study evaluations and results (e.g. atopic dermatitis, bullous pemphigoid, dermatitis herpetiformis, senile pruritus, etc.).<br>Prior exposure to ligelizumab or omalizumab.<br>H1-AH used as background medication at greater than locally label-approved doses after visit 1<br><br>Sex/Gender: ALL<br><br>Ages: 12 Years and older (Child, Adult, Older Adult)<br><br>Enrollment size (Estimated): 1050<br><br>Arms and Interventions:<br>Experimental: Ligelizumab 120 mg<br>Ligelizumab 120 mg arm: 1 injection of 1.0 mL ligelizumab + 1 injection of 1.0 mL ligelizumab placebo q4w<br>Interventions:<br>Biological: Ligelizumab<br>Experimental: Ligelizumab 72 mg<br>Ligelizumab 72 mg arm: 1 injection of 0.6 mL ligelizumab + 1 injection of 1.0 mL ligelizumab placebo q4w<br>Interventions:<br>Biological: Ligelizumab<br>Active Comparator: Omalizumab 300 mg<br>Omalizumab 300 mg arm: 2 injections of 1.2 mL omalizumab q4w<br>Interventions:<br>Biological: Omalizumab<br>Placebo Comparator: Placebo<br>Placebo-ligelizumab arm: 2 injections of 1.0mL of ligelizumab placebo from Week 0 through Week 20; 1 injection of 1.0mL of ligelizumab 120 mg + 1 injection of 1.0 mL ligelizumab placebo from Week 24 through Week 48<br>Interventions:<br>Other: Placebo<br><br>Primary Outcome Measures:<br>Mean Change From Baseline in UAS7 at Week 12 (Multiple

	Biological: Ligelizumab. Liquid in vial<br>Biological: Omalizumab. Lyophilized powder for solution in vial<br>Other: Placebo. Liquid in vial	participants are those most likely to benefit from the intervention, specifically targeting those with CSU inadequately controlled by standard treatments.<br>Exclusion Criteria: Aimed at minimizing confounding factors and ensuring participant safety, particularly excluding those with conditions that could mimic CSU or affect the study outcome.<br>Sex/Gender: Including all genders ensures the findings are applicable across the gender spectrum.<br>Ages: Starting from 12 years allows for adolescent inclusion, increasing the applicability of findings across a wider age range.<br>Enrollment size: Based on the need to ensure statistical power to detect differences between treatment arms.<br>Arms and Interventions: Designed to compare the efficacy of ligelizumab against both an active comparator (omalizumab) and placebo, providing a robust assessment of its efficacy and safety.<br>Primary and Secondary Outcome Measures: Chosen to comprehensively assess the impact of the intervention on disease activity and quality of life, important aspects of treatment efficacy and patient-centered outcomes.	Imputation) of Adult Subjects [Time Frame: Baseline, Week 12]<br>The Urticaria Activity Score (UAS) is sum of the Hive Severity Score (HSS) and the Itch Severity Score (ISS). UAS7 is sum of the HSS7 and the ISS7 scores. Possible range of weekly UAS7 score is 0 to 42. Complete UAS7 response is UAS7 = 0.<br>Hives Severity Score (HSS) scale is 0 to 3. A weekly score (HSS7) is derived by adding up the average daily scores of the 7 days preceding the visit. Possible range of the weekly score is therefore 0 to 21. Hives Severity Score scale: 0 - None 1 - Mild (1-6 hives/12 hours) 2 - Moderate (7-12 hives/12 hours) 3 - Severe (>12 hives/12 hours).<br>Itch Severity Score (ISS) scale is 0 to 3. Score (ISS7) is derived by adding up average daily scores of 7 days preceding visit. Possible range of weekly score is therefore 0 to 21. Itch Severity Score scale: 0 - None 1 - Mild (minimal awareness, easily tolerated) 2 - Moderate (definite awareness, bothersome but tolerable) 3 - Severe (difficult to tolerate).<br>Negative change from baseline indicates improvement<br>Mean Change From Baseline in UAS7 at Week 12 (Observed Data) of Adolescent Subjects (FAS) [Time Frame: Baseline, Week 12]<br>The Urticaria Activity Score (UAS) is sum of the Hive Severity Score (HSS) and the Itch Severity Score (ISS). UAS7 is sum of the HSS7 and the ISS7 scores. Possible range of weekly UAS7 score is 0 to 42. Complete UAS7 response is UAS7 = 0.<br>Hives Severity Score (HSS) scale is 0 to 3. A weekly score (HSS7) is derived by adding up the average daily scores of the 7 days preceding the visit. Possible range of the weekly score is therefore 0 to 21. Hives Severity Score scale: 0 - None 1 - Mild (1-6 hives/12 hours) 2 - Moderate (7-12 hives/12 hours) 3 - Severe (>12 hives/12 hours).

			Itch Severity Score (ISS) scale is 0 to 3. Score (ISS7) is derived by adding up average daily scores of 7 days preceding visit. Possible range of weekly score is therefore 0 to 21. Itch Severity Score scale: 0 - None 1 - Mild (minimal awareness, easily tolerated) 2 - Moderate (definite awareness, bothersome but tolerable) 3 - Severe (difficult to tolerate).<br>Negative change from baseline indicates improvement<br><br>Secondary Outcome Measures:<br>Number and Proportion of Subjects With UAS7=0 Response at Week 12 (Multiple Imputation - Adults, Observed Data for Adolescents) [Time Frame: Week 12]<br>The Urticaria Activity Score (UAS) is the sum of the Hive Severity Score (HSS) and the Itch Severity Score (ISS). UAS7 is the sum of the HSS7 and the ISS7 scores. The possible range of the weekly UAS7 score is 0 to 42. Complete UAS7 response is defined as UAS7 = 0.<br>No Statistical analysis was planned for adolescent group.<br>Mean Change From Baseline in ISS7 at Week 12 (Multiple Imputation) of Adult Subjects (FAS) [Time Frame: Baseline, Week 12]<br>Improvement of severity of itch assessed as absolute change from baseline in ISS7 score at Week 12 Itch Severity Score (ISS) is on a scale of 0 to 3. A weekly score (ISS7) is derived by adding up the average daily scores of the 7 days preceding the visit. The possible range of the weekly score is therefore 0 to 21. Itch Severity Score scale: 0 - None 1 - Mild (minimal awareness, easily tolerated) 2 - Moderate (definite awareness, bothersome but tolerable) 3 - Severe (difficult to tolerate)<br>Negative change from baseline indicates improvement.<br>Mean Change From Baseline in ISS7 at Week 12 (Observed Data) of Adolescent Subjects, (FAS) [Time Frame: Baseline, Week 12]

			Improvement of severity of itch assessed as absolute change from baseline in ISS7 score at Week 12 Itch Severity Score (ISS) is on a scale of 0 to 3. A weekly score (ISS7) is derived by adding up the average daily scores of the 7 days preceding the visit. The possible range of the weekly score is therefore 0 to 21. Itch Severity Score scale: 0 - None 1 - Mild (minimal awareness, easily tolerated) 2 - Moderate (definite awareness, bothersome but tolerable) 3 - Severe (difficult to tolerate)<br>Negative change from baseline indicates improvement.. No Statistical Analysis was planned for adolescent population.<br>Number and Proportion of Participants With DLQI Score of 0 - 1 at Week 12 (Multiple Imputation - Adults, Observed Data for Adolescents) [Time Frame: Baseline, Week 12]<br>Assessed as percentage of subjects achieving DLQI = 0-1, meaning, no impact on subjects quality of life at Week 12<br>The Dermatology life Quality Index (DLQI) score range is 0 to 30, with 0 (meaning no impact of skin disease on quality of life) to 30 (meaning maximum impact on quality of life). No statistical anaylsis was planned for adolescent group.<br>Cumulative Number of Weeks of AAS7=0 up to Week 12 (Multiple Imputation) of Adult Subjects (FAS) [Time Frame: Baseline, Week 12]<br>Cumulative number of weeks that subjects achieve AAS7 = 0 responses between baseline and Week 12<br>Angioedema Activity Score (AAS7) is a measure of the frequency and intensity of angioedema episodes. The total possible range of scores over 7 days is 0-15 (mean day sum score) where higher scores indicate increased angioedema activity.<br>Cumulative Number of Weeks of AAS7=0 up to Week 12 (Observed Data) of Adolescent Subjects (FAS) [Time Frame: Baseline, Week 12]

			Cumulative number of weeks that subjects achieve AAS7 = 0 responses between baseline and Week 12<br>Angioedema Activity Score (AAS7) is a measure of the frequency and intensity of angioedema episodes. The total possible range of scores over 7 days is 0-15 (mean day sum score) where higher scores indicate increased angioedema activity.<br>No Statistical Analysis was planned.
9	Initial Trial Description<br>Official Title: A Phase 3, Randomized, Double-blind, Parallel-group, Placebo-controlled, Multicenter Study to Evaluate the Efficacy and Safety of KarXT in Acutely Psychotic Hospitalized Adults With DSM-5 Schizophrenia<br><br>Brief Summary: This is a Phase 3, randomized, double-blind, parallel-group, placebo-controlled, multicenter inpatient study to examine the efficacy and safety of KarXT in adult subjects who are acutely psychotic with a Diagnostic and Statistical Manual Fifth Edition (DSM-5) diagnosis of schizophrenia. The primary objective of the study is to assess the efficacy of KarXT (a fixed combination of xanomeline 125 mg and trospium chloride 30 mg twice daily [BID]) versus placebo in reducing Positive and Negative Syndrome Scale (PANSS) total scores	Inclusion Criteria:<br>Adults aged 18-65 years.<br>Diagnosed with schizophrenia according to DSM-5 criteria.<br>Currently experiencing an acute psychotic episode.<br>PANSS total score of ≥70 at screening.<br>Able to provide informed consent.<br><br>Exclusion Criteria:<br>Diagnosis of schizoaffective disorder, bipolar disorder, or any psychotic disorder not due to schizophrenia.<br>History of treatment resistance (defined as lack of significant clinical improvement despite two trials of antipsychotic medication at adequate doses and duration).<br>Serious risk of suicidal or homicidal behavior.<br>Known allergy or hypersensitivity to xanomeline, trospium chloride, or related compounds.<br>Pregnant or breastfeeding women.<br>Severe and unstable medical conditions.<br><br>Sex/Gender: ALL<br><br>Ages: 18-65 years	Inclusion Criteria:<br>Subject is aged 18 to 65 years, inclusive, at screening.<br>Subject is capable of providing informed consent.<br>A signed informed consent form must be provided before any study assessments are performed.<br>Subject must be fluent (oral and written) in English to consent<br>Subject has a primary diagnosis of schizophrenia established by a comprehensive psychiatric evaluation based on the DSM-5 criteria and confirmed by Mini International Neuropsychiatric Interview for Schizophrenia and Psychotic Disorder Studies (MINI) version 7.0.2.<br>Subject is experiencing an acute exacerbation or relapse of psychotic symptoms, with onset less than 2 months before screening.<br>The subject requires hospitalization for this acute exacerbation or relapse of psychotic symptoms.<br>If already an inpatient at screening, has been hospitalized for less than 2 weeks for the current exacerbation at the time of screening.<br>Positive and Negative Syndrome Scale total score between 80 and 120, inclusive. Score of ≥4 (moderate or greater) for ≥2 of the following Positive Scale (P) items:<br>Item 1 (P1; delusions)<br>Item 2 (P2; conceptual disorganization)<br>Item 3 (P3; hallucinatory behavior)<br>Item 6 (P6; suspiciousness/persecution)<br>Subjects with no change (improvement) in PANSS total score

	in adult inpatients with a DSM-5 diagnosis of schizophrenia. The secondary objectives of the study are to evaluate improvement in disease severity and symptoms, safety and tolerability, and pharmacokinetics in adult inpatients with a DSM-5 diagnosis of schizophrenia.<br><br>Study Type: Interventional<br><br>Study Phase: Phase 3<br><br>Study Design:<br>Allocation: Randomized<br>Interventional Model: Parallel Assignment<br>Masking: Triple (Participant Investigator Outcomes Assessor)<br>Primary Purpose: Treatment<br><br>Conditions:<br>Schizophrenia<br>Schizophrenia; Psychosis<br><br>Intervention / Treatment:<br>Drug: Xanomeline and Trospium Chloride Capsules<br>Oral xanomeline 50 mg/trospium chloride 20 mg BID (twice a day) for the first 2 days (Days 1 and 2) followed by xanomeline 100 mg/trospium chloride 20 mg BID for the remainder of Week 1 (Days 3 to 7). At Visit	Enrollment size (Estimated): 300<br><br>Arms and Interventions:<br>Participant Group/Arm 1: KarXT (Xanomeline and Trospium Chloride Capsules)<br>Intervention/Treatment: Oral xanomeline 50 mg/trospium chloride 20 mg BID for the first 2 days, followed by xanomeline 100 mg/trospium chloride 20 mg BID for the remainder of Week 1. At Day 8, dose titrated to xanomeline 125 mg/trospium chloride 30 mg BID, with the option to return to 100/20 BID based on tolerability.<br>Participant Group/Arm 2: Placebo<br>Intervention/Treatment: Placebo Capsules BID<br><br>Primary Outcome Measures:<br>Outcome Measure: Change in PANSS total score from baseline to endpoint (6 weeks)<br>Measure Description: The difference in PANSS total scores from the start of the study to the end of the 6-week treatment period.<br>Time Frame: 6 weeks<br><br>Secondary Outcome Measures:<br>Outcome Measure 1: Improvement in Clinical Global Impressions - Severity scale (CGI-S)<br>Measure Description: Change in CGI-S score from baseline to study endpoint.<br>Time Frame: 6 weeks	between screening and baseline (Day -1) of more than 20%.<br>Subject has a CGI-S score of ≥4 at screening and baseline (Day -1) visits.<br>Subject will have been off lithium therapy for at least 2 weeks before baseline and free of all oral antipsychotic medications for at least 5 half-lives or 1 week, whichever is longer, before baseline (Day -1).<br>Subjects taking a long-acting injectable antipsychotic could not have received a dose of medication for at least 12 weeks (24 weeks for INVEGA TRINZA) before baseline visit (Day -1).<br>Subject is willing and able to be confined to an inpatient setting for the study duration, follow instructions, and comply with the protocol requirements.<br>BMI must be ≥18 and ≤40 kg/m2.<br>Subject resides in a stable living situation and is anticipated to return to that same stable living situation after discharge, in the opinion of the investigator.<br>Subject has an identified reliable informant.<br>Women of childbearing potential, or men with sexual partners of childbearing potential, must be able and willing to use at least 1 highly effective method of contraception during the study and for 30 days after the last dose of study drug. Sperm donation is not allowed for 30 days after the final dose of study drug.<br><br>Exclusion Criteria:<br>Any primary DSM-5 disorder other than schizophrenia within 12 months before screening (confirmed using MINI version 7.0.2 at screening).<br>Symptoms of mild mood dysphoria or anxiety are allowed as long as these symptoms are not the primary focus of treatment. A screening subject with mild substance abuse disorder within the 12 months before screening must be discussed and agreed upon with the medical monitor before they can be allowed into the study.

	5 (Day 8), dosing was to be titrated upwards to xanomeline 125 mg/trospium chloride 30 mg BID unless the subject was continuing to experience adverse events (AEs) from the previous dose of KarXT 100/20 BID. All subjects who were increased to KarXT 125/30 BID, depending on clinical response and tolerability, had the option to return to KarXT 100/20 BID for the remainder of the treatment period.<br>Other Names: KarXT<br>Drug: Placebo<br>Placebo Capsules twice a day (BID)<br>Other Names: PBO	Outcome Measure 2: Safety and Tolerability<br>Measure Description: Incidence of adverse events, changes in vital signs, laboratory findings, and ECG parameters.<br>Time Frame: 6 weeks<br><br>Clinical Reasoning:<br><br>Inclusion Criteria:<br>Focused on adults within the age range most commonly affected by schizophrenia and experiencing acute psychosis to ensure the study population is representative of those who might benefit most from the intervention.<br>PANSS score requirement ensures participants have a significant level of symptoms that can be quantitatively measured for changes.<br><br>Exclusion Criteria:<br>Excluding those with other psychotic disorders or treatment-resistant schizophrenia ensures a more homogenous study population, reducing variability in response due to different underlying pathologies or previous treatment failures.<br>Excluding those with severe medical conditions or risks ensures participant safety and reduces confounding factors related to adverse events.<br><br>Sex/Gender and Ages:	Subjects who are newly diagnosed or are experiencing their first treated episode of schizophrenia.<br>History or presence of clinically significant cardiovascular, pulmonary, hepatic, renal, hematologic, gastrointestinal, endocrine, immunologic, dermatologic, neurologic, or oncologic disease or any other condition that, in the opinion of the investigator, would jeopardize the safety of the subject or the validity of the study results.<br>Subjects with HIV, cirrhosis, biliary duct abnormalities, hepatobiliary carcinoma, and/or active hepatic viral infections based on either medical history or liver function test results.<br>History or high risk of urinary retention, gastric retention, or narrow-angle glaucoma.<br>History of irritable bowel syndrome (with or without constipation) or serious constipation requiring treatment within the last 6 months.<br>Risk for suicidal behavior during the study as determined by the investigator's clinical assessment and Columbia-Suicide Severity Rating Scale (C-SSRS).<br>Clinically significant abnormal finding on the physical examination, medical history, ECG, or clinical laboratory results at screening.<br>Subjects cannot currently (within 5 half-lives or 1 week, whichever is longer, before baseline [Day -1]) be receiving oral antipsychotic medications; monoamine oxidase inhibitors; anticonvulsants (eg, lamotrigine, Depakote); tricyclic antidepressants (eg, imipramine, desipramine); selective serotonin reuptake inhibitors; or any other psychoactive medications except for as needed anxiolytics (eg, lorazepam, chloral hydrate).<br>Pregnant, lactating, or less than 3 months postpartum.<br>If, in the opinion of the investigator (and/or Sponsor), subject is unsuitable for enrollment in the study or subject has any finding that, in the view of the investigator (and/or Sponsor), may compromise the

		Including all genders and a wide age range increases the generalizability of the study findings to the broader population of adults with schizophrenia.<br><br>Enrollment Size:<br>A sample size of 300 is estimated to provide sufficient power to detect a clinically significant difference between the treatment and placebo groups, accounting for potential dropouts.<br><br>Arms and Interventions:<br>The design includes a titration period to minimize adverse effects, improving tolerability and adherence to the study medication.<br><br>Outcome Measures:<br>Primary and secondary outcome measures are chosen to comprehensively assess the efficacy (via PANSS and CGI-S) and safety/tolerability of the intervention, which are critical for evaluating the potential benefits and risks of KarXT in this population.	safety of the subject or affect his/her ability to adhere to the protocol visit schedule or fulfill visit requirements.<br>Positive test for coronavirus (COVID-19) within 2 weeks before screening and at screening.<br>Subjects with extreme concerns relating to global pandemics, such as COVID-19, that preclude study participation.<br>Subject has had psychiatric hospitalization(s) for more than 30 days (cumulative) during the 90 days before screening.<br>Subject has a history of treatment resistance to schizophrenia medications defined as failure to respond to 2 adequate courses of pharmacotherapy (a minimum of 4 weeks at an adequate dose per the label) or required clozapine within the last 12 months.<br>Subjects with prior exposure to KarXT.<br>Subjects who experienced any adverse effects due to xanomeline or trospium.<br>Participation in another clinical study in which the subject received an experimental or investigational drug agent within 3 months before screening.<br>Risk of violent or destructive behavior.<br>Current involuntary hospitalization or incarceration.<br><br>Sex/Gender: All<br><br>Ages: 18 Years to 65 Years (Adult, Older Adult)<br><br>Enrollment size (Estimated): 246<br><br>Arms and Interventions:<br>Experimental: KarXT<br>Interventions:<br>Drug: Xanomeline and Trospium Chloride Capsules<br>Placebo Comparator: Placebo<br>Interventions:<br>Drug: Placebo<br><br>Primary Outcome Measures:

			Change From Baseline in Positive and Negative Syndrome Scale (PANSS) Total Score at Week 5 [Time Frame: Baseline and Week 5] The PANSS is a medical scale used for measuring symptom severity of participants with schizophrenia. The PANSS rating form contains 7 positive symptom scales, 7 negative system scales, and 16 general psychopathology symptom scales. Participants are rated from 1 to 7 on each symptom scale. It takes approximately 45 to 50 minutes to administer. The total score is the sum of all scales with a minimum score of 30 and a maximum score of 210. A decrease in PANSS total score correlates with an improvement in schizophrenia symptoms.<br><br>Secondary Outcome Measures:<br>Change From Baseline in Positive and Negative Syndrome Scale (PANSS) Positive Score at Week 5 [Time Frame: Baseline and Week 5] The PANSS is a medical scale used for measuring symptom severity of participants with schizophrenia. The PANSS rating form contains 7 positive symptom scales, 7 negative system scales, and 16 general psychopathology symptom scales. Participants are rated from 1 to 7 on each symptom scale. For positive symptoms in schizophrenia, participants are rated from 1 to 7 on each symptom scale, with a minimum score of 7 and a maximum score of 49. A decrease in PANSS total score correlates with an improvement in schizophrenia symptoms.<br>Change From Baseline in Positive and Negative Syndrome Scale (PANSS) Negative Score at Week 5 [Time Frame: Baseline and Week 5] The PANSS rating form contains 7 positive symptom scales, 7 negative system scales, and 16 general psychopathology symptom scales. For negative symptoms in schizophrenia, participants are rated from 1 to 7 on each symptom scale, with a minimum score of 7 and a maximum score of 49. A decrease in

			PANSS total score correlates with an improvement in schizophrenia symptoms.<br>Change From Baseline in Positive and Negative Syndrome Scale (PANSS) Marder Factor Negative Score [Time Frame: Baseline and Week 5]<br>The Marder Factor Negative Score is derived from the Positive and Negative Syndrome Scale (PANSS) and consists of the sum of 5 negative scales (N) and 2 general scales (G) (N1. Blunted affect; N2. Emotional withdrawal; N3. Poor rapport; N4. Passive/apathetic social withdrawal; N6. Lack of spontaneity; G7. Motor retardation; and G16. Active social avoidance), with a minimum score of 7 and a maximum score of 49. A decrease in PANSS total score correlates with an improvement in schizophrenia symptoms.<br>Change From Baseline Clinical Global Impression - Severity (CGI-S) Score at Week 5 [Time Frame: Baseline and Week 5]<br>The CGI-S modified asked the clinician 1 question: "Considering your total clinical experience, how mentally ill is the participant at this time?" The clinician's answer rated on the following 7-point scale: 1 = normal, not at all ill; 2 = borderline mentally ill; 3 = mildly ill; 4 = moderately ill; 5 = markedly ill; 6 = severely ill; 7 = among the most extremely ill participants.<br>Percentage of Positive and Negative Syndrome Scale (PANSS) Responders (>=30% Change in PANSS Total Score) at Week 5 [Time Frame: Baseline and Week 5]<br>The PANSS is a medical scale used for measuring symptom severity of participants with schizophrenia. The PANSS rating form contains 7 positive symptom scales, 7 negative system scales, and 16 general psychopathology symptom scales. Participants are rated from 1 to 7 on each symptom scale. The total score is the sum of all scales with a minimum score of 30 and a maximum score of 210. A PANSS responder is

			defined as a participant with at least a 30% change in PANSS total score compared to baseline at Week 5.
10	Initial Trial Description<br>Official Title: A PHASE 3, RANDOMIZED, DOUBLE-BLIND, PLACEBO-CONTROLLED STUDY OF TALAZOPARIB WITH ENZALUTAMIDE IN METASTATIC CASTRATION-RESISTANT PROSTATE CANCER<br><br>Brief Summary: This study compares rPFS in men with mCRPC treated with talazoparib plus enzalutamide vs. enzalutamide after confirmation of the starting dose of talazoparib in combination with enzalutamide.<br><br>Study Type: Interventional<br><br>Study Phase: Phase 3<br><br>Study Design: Allocation: Randomized<br>Interventional Model: Parallel Assignment<br>Interventional Model Description: To assess radiographic PFS in men with mCRPC (with no systemic treatments initiated after documentation of mCRCP) treated with talazoparib and	Inclusion Criteria:<br>Histologically or cytologically confirmed metastatic castration-resistant prostate cancer (mCRPC).<br>Age ≥ 18 years.<br>ECOG performance status of 0 or 1.<br>Adequate organ and marrow function.<br>Patients must have measurable disease as per RECIST 1.1 criteria or bone lesions on bone scan.<br>Prior treatment with at least one next-generation anti-androgen therapy (e.g., abiraterone, apalutamide).<br><br>Exclusion Criteria:<br>Prior treatment with PARP inhibitors or chemotherapy for mCRPC.<br>Active brain metastases or leptomeningeal disease.<br>Known hypersensitivity to talazoparib, enzalutamide, or any excipient contained in the drug formulation.<br>Concurrent use of other anti-cancer therapies.<br>Significant cardiovascular disease within 6 months prior to enrollment.<br><br>Sex/Gender: Male<br><br>Ages: 18 years and older<br><br>Enrollment size (Estimated): 300	Inclusion Criteria:<br>Histologically or cytologically confirmed adenocarcinoma of the prostate without small cell or signet cell features<br>Asymptomatic or mildly symptomatic metastatic castration resistant prostate cancer (mCRPC) (score on BPI-SF Question #3 must be < 4).<br>For enrollment into Part 2 only (optional in Part 1): assessment of DDR mutation status<br>Consent to a saliva sample collection for a germline comparator unless prohibited by local regulations or ethics committee decision (optional for patients in Part 1).<br>Surgically or medically castrated, with serum testosterone ≤ 50 ng/dL (≤ 1.73 nmol/L) at screening.<br>Metastatic disease in bone documented on bone scan or in soft tissue documented on CT/MRI scan.<br>Progressive disease at study entry in the setting of medical or surgical castration as defined by 1 or more of the following 3 criteria:<br>Prostate specific antigen (PSA) progression defined by a minimum of 2 rising PSA values from 3 consecutive assessments with an interval of at least 7 days between assessments..<br>Soft tissue disease progression as defined by RECIST 1.1.<br>Bone disease progression defined by Prostate Cancer Working Group 3 (PCWG3) with 2 or more new metastatic bone lesions on a whole body radionuclide bone scan.<br>Ongoing bisphosphonate or denosumab use prior to Day 1 (Part 1) or randomization (Part 2) is allowed but not mandatory.<br>Eastern Cooperative Oncology Group (ECOG) performance status ≤ 1.<br>Life expectancy ≥ 12 months as assessed by the investigator.<br>Able to swallow the study drug and have no known intolerance to study drugs or excipients.

	enzalutamide vs. placebo plus enzalutamide<br>Masking: Quadruple (Participant Care Provider Investigator Outcomes Assessor)<br>Masking Description: Double-blind<br>Primary Purpose: Treatment<br><br>Conditions: mCRPC<br><br>Intervention / Treatment:<br>Drug: Talazoparib with enzalutamide Talazoparib 0.5 mg/day plus enzalutamide 160mg/day<br>Drug: Placebo with enzalutamide Placebo plus enzalutamide 160 mg/day	Arms and Interventions:<br>Arm A (Experimental Group): Participants will receive Talazoparib 0.5 mg/day plus Enzalutamide 160mg/day.<br>Arm B (Control Group): Participants will receive Placebo plus Enzalutamide 160 mg/day.<br><br>Primary Outcome Measures:<br>Radiographic Progression-Free Survival (rPFS): Time from randomization to radiographic progression or death from any cause, whichever comes first, assessed up to 24 months.<br><br>Secondary Outcome Measures:<br>Overall Survival (OS): Time from randomization to death from any cause, assessed up to 36 months.<br>Prostate-Specific Antigen (PSA) Response: Proportion of participants with a ≥ 50% reduction in PSA from baseline, assessed at 3, 6, and 12 months.<br>Quality of Life (QoL) Assessments: Changes in health-related quality of life scores using validated instruments, assessed at baseline, 3, 6, 12, and 24 months.<br><br>Clinical Reasoning:<br><br>Inclusion Criteria: The inclusion criteria are designed to select a homogeneous group of patients with confirmed mCRPC, who are	Must agree to use a condom when having sex with a partner from the time of the first dose of study drug through 4 months after last dose of study treatment. Must also agree for female partner of childbearing potential to use an additional highly effective form of contraception from the time of the first dose of study treatment through 4 months after last dose of study treatment when having sex with a non pregnant female partner of childbearing potential.<br>Must agree not to donate sperm from the first dose of study drug to 4 months after the last dose of study drug.<br>Evidence of a personally signed and dated informed consent document (and molecular prescreening consent if appropriate) indicating that the patient [or a legally acceptable representative/legal guardian] has been informed of all pertinent aspects of the study.<br>Willing and able to comply with scheduled visits, treatment plan, laboratory tests, and other study procedures<br><br>Exclusion Criteria:<br>Any prior systemic cancer treatment initiated in in the non metastatic CRPC and mCRPC disease state.<br>Patients whose only evidence of metastasis is adenopathy below the aortic bifurcation.<br>Prior treatment with second-generation androgen receptor inhibitors (enzalutamide, apalutamide, and darolutamide), a PARP inhibitor, cyclophosphamide, or mitoxantrone for prostate cancer.<br>Prior treatment with platinum-based chemotherapy within 6 months (from the last dose) prior to Day 1 (Part 1) or randomization (Part 2), or any history of disease progression on platinum-based therapy within 6 months (from the last dose).<br>Treatment with cytotoxic chemotherapy, biologic therapy including sipuleucel T, or radionuclide therapy received in the castration-sensitive prostate cancer is NOT

		relatively healthy and have a potential to benefit from the treatment. Including patients with measurable disease ensures that changes in tumor burden can be accurately assessed.<br><br>Exclusion Criteria: Excluding patients with prior treatment with PARP inhibitors or chemotherapy for mCRPC ensures that the study evaluates the efficacy of talazoparib in a naïve population. Patients with brain metastases are excluded due to the poor prognosis and different disease biology, which could confound study results.<br><br>Sex/Gender: The trial is limited to males, as prostate cancer does not occur in females.<br><br>Ages: Setting the age limit to 18 years and older allows for the inclusion of all adult males with mCRPC, ensuring a broad applicability of the trial results.<br><br>Enrollment size: An estimated enrollment size of 300 participants is chosen to ensure adequate power to detect a significant difference in rPFS between the two arms, taking into account potential dropouts.<br><br>Arms and Interventions: The experimental design includes a comparison	exclusionary if discontinued in the 28 days prior to Day 1 (Part 1) or randomization (Part 2).<br>Treatment with any investigational agent within 4 weeks before Day 1 (Part 1) or randomization (Part 2).<br>Prior treatment with opioids for pain related to either primary prostate cancer or metastasis within 28 days prior to Day 1 (Part 1) or randomization (Part 2).<br>Current use of potent P-gp inhibitors within 7 days prior to Day 1 (Part 1) or randomization (Part 2).<br>Major surgery (as defined by the investigator) within 2 weeks before Day 1 (Part 1) or randomization (Part 2), or palliative localized radiation therapy within 3 weeks before randomization (Part 2).<br>Clinically significant cardiovascular disease<br>Significant renal dysfunction as defined by any of the following laboratory abnormalities:<br>• Renal: eGFR < 30 mL/min/1.73 m2 by the MDRD equation (available via www.mdrd.com).<br>Patients enrolled in Part 1 only: Moderate renal impairment (eGFR 30-59 mL/min/1.73 m2) at screening.<br>Significant hepatic dysfunction as defined by any of the following laboratory abnormalities on screening labs:<br>Total serum bilirubin >1.5 times the upper limit of normal (ULN) (>3 × ULN for patients with documented Gilbert syndrome or for whom indirect bilirubin concentrations suggest an extrahepatic source of elevation).<br>Aspartate aminotransferase (AST) or alanine aminotransferase (ALT) >2.5 times ULN (>5 × ULN if liver function abnormalities are due to hepatic metastasis).<br>Albumin <2.8 g/dL<br>Absolute neutrophil count < 1500/μL, platelets < 100,000/μL, or hemoglobin < 9 g/dL (may not have received growth factors or blood transfusions within 14 days before obtaining the hematology values at screening).

		between the combination of talazoparib with enzalutamide and enzalutamide with placebo, which allows for the evaluation of the added benefit of talazoparib.<br><br>Primary and Secondary Outcome Measures: The primary outcome measure focuses on rPFS, a clinically meaningful endpoint in mCRPC trials. Secondary outcomes include overall survival, PSA response, and quality of life assessments, which are important for understanding the broader impact of the treatment on patients' health and well-being.	Known or suspected brain metastasis or active leptomeningeal disease.<br>Symptomatic or impending spinal cord compression or cauda equina syndrome.<br>Any history of myelodysplastic syndrome, acute myeloid leukemia, or prior malignancy except any of the following:<br>Carcinoma in situ or non melanoma skin cancer<br>Any prior malignancies ≥3 years before randomization with no subsequent evidence of recurrence or progression regardless of the stage.<br>Stage 0 or Stage 1 cancer <3 years before randomization that has a remote probability of recurrence or progression in the opinion of the investigator<br>Gastrointestinal disorder affecting absorption.<br>Fertile male subjects who are unwilling or unable to use highly effective methods of contraception for the duration of the study and for 4 months after the last dose of investigational product.<br>Investigator site staff members directly involved in the conduct of the study and their family members, site staff members otherwise supervised by the investigator, or patients who are Pfizer employees, including their family members, directly involved in the conduct of the study.<br>Other acute or chronic medical (concurrent disease, infection, or comorbidity) or psychiatric condition including recent (within the past year) or active suicidal ideation or behavior or laboratory abnormality that interferes with ability to participate in the study, may increase the risk associated with study participation or investigational product administration, or may interfere with the interpretation of study results and, in the judgment of the investigator, would make the patient inappropriate for entry into this study.<br>History of seizure or any condition that may predispose to seizure (eg, prior cortical stroke, significant brain

			trauma). Also, history of loss of consciousness or transient ischemic attack within 12 months of randomization (Part 2).<br><br>Sex/Gender: Male<br><br>Ages: 18 Years and older (Adult, Older Adult)<br><br>Enrollment size (Estimated): 444<br><br>Arms and Interventions:<br>Experimental: Combination arm<br>Talazoparib plus enzalutamide<br>Interventions:<br>Drug: Talazoparib with enzalutamide<br>Active Comparator: Monotherapy arm<br>Ezalutamide plus placebo<br>Interventions:<br>Drug: Placebo with enzalutamide<br><br>Primary Outcome Measures:<br>Number of Participants With Treatment-Emergent Adverse Events (TEAEs) Occuring Within the First 66 Days of Dosing - Part 1 [Time Frame: Post dose on Day 1 up to Day 66 in Part 1]<br>An adverse event (AE) was any untoward medical occurrence in a participant who received study intervention without regard to possibility of causal relationship. TEAEs are defined as newly occurring AEs or those worsening after first dose. As per Common Terminology Criteria for Adverse Events (CTCAE) version 4, Grade 1= mild AE; Grade 2= moderate AE; Grade 3= severe AE; Grade 4= life-threatening or disabling AE; Grade 5= death related to an AE. Serious TEAE (SAE) was an AE resulting in any of the following outcomes or deemed significant for any other reason: death; initial or prolonged inpatient hospitalization; life-threatening experience (immediate risk of dying); persistent or significant disability/incapacity; congenital anomaly. SAEs were determined according to the investigator's assessment. Results as of 16 Aug 2022 are reported.

			Number of Participants With All-Causality Clustered Treatment-Emergent Cytopenias by Preferred Term (PT) and Max CTCAE Grade Occuring Within the First 66 Days of Dosing - Part 1 [Time Frame: Post dose on Day 1 up to Day 66 in Part 1] An AE was any untoward medical occurrence in a participant who received study intervention without regard to possibility of causal relationship. TEAEs are defined as newly occurring AEs or those worsening after first dose. As per CTCAE version 4, Grade 1=mild AE; Grade 2=moderate AE; Grade 3=severe AE; Grade 4=life-threatening or disabling AE; Grade 5=death related to an AE. Medical Dictionary for Regulatory Activities (MedDRA) v25.0 coding dictionary applied. PTs for the cluster terms are: ANEMIA, including Anemia, Hematocrit decreased, Hemoglobin decreased, and Red blood cell count decreased; THROMBOCYTOPENIA, including, Thrombocytopenia and Platelet count decreased; NEUTROPENIA, including Febrile neutropenia, Neutropenia and Neutrophil count decreased; LEUKOPENIA, including Leukopenia, White blood cell count decreased. Events in any grade with at least 1 occurrence in participants are reported for this outcome measure. Results as of 16 Aug 2022 are reported.<br>Number of Participants With All-Causality TEAEs During the Overall Period of Part 1 [Time Frame: Post dose on Day 1 up to 28 days after the last dose of study intervention, or before new systemic antineoplastic therapy, whichever occurred first (maximum of 235.14 weeks)]<br>An adverse event (AE) was any untoward medical occurrence in a participant who received study intervention without regard to possibility of causal relationship. TEAEs are defined as newly occurring AEs or those worsening after first dose. As per CTCAE version 4, Grade 1= mild AE; Grade

			2= moderate AE; Grade 3= severe AE; Grade 4= life-threatening or disabling AE; Grade 5= death related to an AE. Serious TEAE (SAE) was an AE resulting in any of the following outcomes or deemed significant for any other reason: death; initial or prolonged inpatient hospitalization; life-threatening experience (immediate risk of dying); persistent or significant disability/incapacity; congenital anomaly. SAEs were determined according to the investigator's assessment. Results as of 16 Aug 2022 are reported.<br>Number of Participants With Treatment-Related TEAEs During the Overall Period of Part 1 [Time Frame: Post dose on Day 1 up to 28 days after the last dose of study intervention, or before new systemic antineoplastic therapy, whichever occurred first (maximum of 235.14 weeks)]<br>An AE was any untoward medical occurrence in a participant who received study intervention without regard to possibility of causal relationship. TEAEs are defined as newly occurring AEs or those worsening after first dose. Treatment-related AE was any untoward medical occurrence attributed to study intervention in a participant who received study intervention. As per CTCAE version 4, Grade 1= mild AE; Grade 2= moderate AE; Grade 3= severe AE; Grade 4= life-threatening or disabling AE; Grade 5= death related to an AE. An SAE was an AE resulting in any of the following outcomes or deemed significant for any other reason: death; initial or prolonged inpatient hospitalization; life-threatening experience (immediate risk of dying); persistent or significant disability/incapacity; congenital anomaly. SAEs were determined according to the investigator's assessment. Results as of 16 Aug 2022 are reported.<br>Number of Participants With All-Causality Clustered Treatment-Emergent Cytopenias by PT and Max

			CTCAE Grade Occuring Anytime After Dosing - Part 1 [Time Frame: Post dose on Day 1 up to 28 days after the last dose of study intervention, or before new systemic antineoplastic therapy, whichever occurred first (maximum of 235.14 weeks)]<br>An AE was any untoward medical occurrence in a participant who received study intervention without regard to possibility of causal relationship. TEAEs are defined as newly occurring AEs or those worsening after first dose. As per CTCAE version 4, Grade 1=mild AE; Grade 2=moderate AE; Grade 3=severe AE; Grade 4=life-threatening or disabling AE; Grade 5=death related to an AE. MedDRA v25.0 coding dictionary applied. PTs for the cluster terms are: ANEMIA, including Anemia, Hematocrit decreased, Hemoglobin decreased, and Red blood cell count decreased; THROMBOCYTOPENIA, including, Thrombocytopenia and Platelet count decreased; NEUTROPENIA, including Febrile neutropenia, Neutropenia and Neutrophil count decreased; LEUKOPENIA, including Leukopenia, White blood cell count decreased. Events in any grade with at least 1 occurrence in participants are reported for this outcome measure. Results as of 16 Aug 2022 are reported.<br>Number of Participants With Treatment-Related Clustered Treatment-Emergent Cytopenias by PT and Max CTCAE Grade in >=10% of Participants Occuring Anytime After Dosing - Part 1 [Time Frame: Post dose on Day 1 up to 28 days after the last dose of study intervention, or before new systemic antineoplastic therapy, whichever occurred first (maximum of 235.14 weeks)]<br>An AE was any untoward medical occurrence in a participant who received study intervention without regard to possibility of causal relationship. TEAEs are newly occurring AEs or those worsening

			after first dose. Treatment-related AE was any AE attributed to study intervention in a participant who received study intervention. As per CTCAE version 4, Grade 1=mild; Grade 2=moderate; Grade 3=severe; Grade 4=life-threatening or disabling; Grade 5=death related to an AE. MedDRA v25.0 coding dictionary applied. PTs for the cluster terms are: ANEMIA, including Anemia, Hematocrit decreased, Hemoglobin decreased, and Red blood cell count decreased; THROMBOCYTOPENIA, including, Thrombocytopenia and Platelet count decreased; NEUTROPENIA, including Febrile neutropenia, Neutropenia and Neutrophil count decreased; LEUKOPENIA, including Leukopenia, White blood cell count decreased. Events in any grade with incidence in >=10% of participants are reported. Results as of 16 Aug 2022 are reported.<br>Blinded Independent Central Review (BICR) Assessed Radiographic Progression-Free Survival (rPFS) Per Response Evaluation Criteria in Solid Tumors (RECIST) 1.1 for All-Comers - Part 2 Cohort 1 [Time Frame: From the start of treatment to the time of first documented progression, or death (maximum up to 42 months)]<br>rPFS is defined as the time from the date of randomization to first objective evidence of radiographic progression as assessed in soft tissue per RECIST 1.1, or death, whichever occurs first. Soft tissue disease status was assessed at regular intervals during the course of the study by computed tomography (CT) of chest and CT or magnetic resonance imaging (MRI) of abdomen and pelvis. Progression is defined using RECIST 1.1 as a >=20% increase in the sum of the longest diameter of target lesions, or a measurable increase in a non-target lesion, or the appearance of new lesions. Results as of 16 Aug 2022 are reported for this outcome measure.

			BICR Assessed rPFS Per RECIST 1.1 in Patients With DDR Deficiencies - Part 2 [Time Frame: From the start of treatment to the time of first documented progression, or death (maximum up to 38 months)] rPFS is defined as the time from the date of randomization to first objective evidence of radiographic progression as assessed in soft tissue per RECIST 1.1, or death, whichever occurs first. Soft tissue disease status was assessed at regular intervals during the course of the study by CT of chest and CT or MRI of abdomen and pelvis. Results as of 03 Oct 2022 are reported for this outcome measure.<br><br>Secondary Outcome Measures:<br>Nil
11	Initial Trial Description<br>Official Title: Colchicine in Acutely Decompensated Heart Failure With Reduced Ejection Fraction: a Pilot Study<br><br>Brief Summary:We propose a double blind, placebo-controlled pilot trial randomizing patients admitted to the hospital with acutely decompensated heart failure (ADHF) and inflammation to receive either colchicine or matching placebo. Upon enrollment, patients will be randomized 1:1 to receive either the experimental drug (Colchicine) or matching placebo. The regimen in the active arm will consist of 14 days of Colchicine 0.6 mg bid followed by 76±14	Inclusion Criteria:<br>Patients admitted to the hospital with a diagnosis of acutely decompensated heart failure (ADHF) confirmed by echocardiography showing reduced ejection fraction (EF ≤40%).<br>Evidence of systemic inflammation indicated by elevated levels of hsCRP (>2 mg/L) at admission.<br>Age 18 years or older.<br>Ability to provide informed consent.<br><br>Exclusion Criteria:<br>Known hypersensitivity or contraindication to colchicine.<br>Pregnancy or lactation.<br>Severe chronic kidney disease (Stage IV or V) not on dialysis.<br>Current use of colchicine for any other indication.<br>Enrollment in another clinical trial that could interfere with the outcomes of this study.	Inclusion Criteria:<br><br>Primary admission diagnosis of acute decompensated heart failure as evidenced by:<br><br>Heart failure symptoms and at least one of the following:<br>Pulmonary congestion/edema at physical exam (or chest radiography)<br>E/e' > 13 on transthoracic echocardiography<br>Left heart catheterization showing elevated left ventricular (LV) end-diastolic pressure >18 mmHg or right heart catheterization showing pulmonary artery occluding pressure (wedge) >16 mmHg<br>Elevated plasma B-type natriuretic peptide (>100 pg/ml) or N-terminal B-type natriuretic peptide (>300 pg/ml)<br>LV systolic dysfunction (left ventricular ejection fraction [LVEF] <40%) during the index hospitalization or prior 12 months;<br>Expected duration of heart failure at least three months<br>Age 18 years or older<br>Willing and able to provide written informed consent<br>Screening plasma CRP >0.3 mg/dL (3 mg/L) or high-sensitivity CRP >2 mg/L

	days of Colchicine 0.6 mg once per day. Placebo regimen will be analogous, with one pill bid for 14 days followed by one pill once per day for 76 days. Dose reduction for patients with Stage III chronic kidney disease is allowed as detailed in the protocol. At the same time, dose reduction can also be elected in case of GI symptoms. We will transiently stop the experimental medication in case of acute kidney injury (AKI), defined per Kidney Disease Improving Global Outcomes (KDIGO) Stage I, as specified in the protocol. These patients will continue with their standard of care for the management of heart failure which consists of a combination of medications that relieve congestion, normalize blood pressure and heart rate, and block the effects of hormones on the heart. The proposed treatment will be in addition to standard of care. No standard of care medications will be withheld. While inflammation is a known risk factor in heart failure, there are no standard anti-inflammatory drugs used in patients with heart failure, as the benefit is not established. We will	Life expectancy less than 3 months due to other comorbid conditions.<br>Severe liver disease (Child-Pugh Class C).<br><br>Sex/Gender: ALL<br><br>Ages: 18 years and older<br><br>Arms and Interventions:<br>Participant Group/Arm 1: Colchicine Group<br>Intervention/Treatment: Drug: Colchicine 0.6 mg as per the described regimen.<br>Participant Group/Arm 2: Placebo Group<br>Intervention/Treatment: Drug: Control/Placebo group as per the described regimen.<br><br>Primary Outcome Measures:<br>Outcome Measure: Change in hsCRP levels from baseline.<br>Measure Description: The primary outcome will assess the anti-inflammatory effect of colchicine by measuring the change in hsCRP levels from baseline to the end of the treatment period (90±14 days).<br>Time Frame: Baseline, 14±7 days, and 90±14 days.<br><br>Secondary Outcome Measures:<br>Outcome Measure: Change in IL-6 levels from baseline.<br>Measure Description: To evaluate the effect of colchicine on IL-6 levels as another marker of inflammation.	Exclusion Criteria:<br><br>Concomitant clinically significant comorbidities that would interfere with the execution or interpretation of the study, including but not limited to acute coronary syndromes, uncontrolled hypertension or orthostatic hypotension, tachy- or brady-arrhythmias, acute or chronic pulmonary disease or neuromuscular disorders affecting respiration<br>Cardiac resynchronization therapy (CRT), coronary artery revascularization procedures, or heart valve surgeries performed within 3 months or planned during the admission<br>Previous or planned implantation of left ventricular assist devices or heart transplantation<br>Chronic use of intravenous inotropes<br>Current or recent (i.e. within 4 half-lives) use of immunosuppressive or anti-inflammatory drugs (not including NSAIDs).<br>Current treatment with colchicine or planned initiation of colchicine therapy in the next three months for gout<br>Chronic inflammatory disorder, including but not limited to rheumatoid arthritis and systemic lupus erythematosus<br>Active infection (of any type)<br>Chronic or recurrent infectious disease, including hepatitis B virus, hepatitis C virus, and HIV/AIDS<br>Prior (within the past 5 years) or current malignancy, with the exclusion of in situ lesion with low potential for progression<br>Any comorbidity leading to expected survival less than three months or inability to complete the study<br>End-stage kidney disease requiring renal replacement therapy<br>Neutropenia (<2,000/mm3) or Thrombocytopenia (<50,000/mm3)<br>Pregnancy<br><br>For all biological females with child bearing potential a pregnancy test will be performed as part of standard of care.

	study colchicine, an anti-inflammatory drug, as compares with placebo. We are planning to obtain blood from the patients in order to measure hsCRP and IL-6. Blood samples will be collected at baseline, 24±6h, 48±6h and 72±6h after treatment initiation, and subsequently at 14±7 days and at study closure. The first four blood samples will be obtained while the subject is still admitted to the hospital. The blood sample at 14±7 days will be obtained during an outpatient encounter. A study closure visit with clinical assessment and experimental drug collection for capsule counting to assess compliance will be conducted at 90±14; the final blood sample will be collected at that time.<br><br>Study Type: Interventional<br><br>Study Phase: Phase 4<br><br>Study Design:<br>Allocation: Randomized<br>Interventional Model: Parallel Assignment<br>Interventional Model Description: We are planning to use controls in the present study. Controls will be patients from the	Time Frame: Baseline, 14±7 days, and 90±14 days.<br>Outcome Measure: Hospital readmission rates for heart failure.<br>Measure Description: To assess the impact of colchicine on the clinical course of ADHF by comparing the rate of hospital readmissions between the two groups.<br>Time Frame: Within 90 days of treatment initiation.<br>Outcome Measure: Change in NYHA functional class.<br>Measure Description: To evaluate the effect of colchicine on patients' functional status.<br>Time Frame: Baseline and 90±14 days.<br><br>Clinical Reasoning:<br><br>Inclusion and Exclusion Criteria: These criteria are designed to select a homogeneous group of patients with ADHF and evidence of inflammation, who could potentially benefit from anti-inflammatory treatment with colchicine. Excluding patients with severe kidney or liver disease minimizes the risk of adverse events related to colchicine metabolism and excretion.<br>Sex/Gender and Ages: Including all genders and adults 18 years and older ensures the study results are generalizable to the broader ADHF population.<br>Enrollment Size: Given the pilot nature of the study, a sample size of	Presence of specific contraindications to colchicine treatment, which may include<br><br>Previous adverse reaction to colchicine<br>Biliary obstruction<br>Renal impairment with estimated glomerular filtration rate (eGFR) <30 ml/min<br>Liver cirrhosis from stage Child-Pugh A to more advanced<br>Prisoners<br>Treatment with medication contraindicated for concomitant use with colchicine per<br>Food and Drugs Administration labeling, including:<br><br>Protease inhibitors<br>Macrolides antibiotic<br>Ketoconazole, Fluconazole and Itraconazole<br>Nefazodone<br>Non-dihydropiridine calcium channel blockers<br>Aprepitant<br>Ranolazine<br>Cyclosporine<br><br>Sex/Gender: All<br><br>Ages: 18 Years and older (Adult, Older Adult)<br><br>Arms and Interventions:<br>Experimental: Colchicine 0.6 mg treatment group<br>Treatment group will be given active drug (0.6mg Colchicine) 2x/day (once if subject has kidney disease) for 14 days. Subsequently treatment group subjects will be given active drug (0.6mg Colchicine) 1x/day for 76 +/- days (or once every other day if subject has kidney disease).<br>Interventions:<br>Drug: Colchicine 0.6 mg<br>Placebo Comparator: Control/Placebo group<br>Control/Placebo group will be given placebo that looks identical to study drug with no active ingredients and will take 2x/day (once if subject has kidney disease) for 14 days.

	target population, i.e. acutely decompensated heart failure with reduced ejection fraction.<br>Masking: Triple (Participant Care Provider Investigator)<br>Primary Purpose: Treatment<br><br>Conditions: Heart Failure<br>Decompensated Heart Failure<br>Heart Failure With Reduced Ejection Fraction<br><br>Intervention / Treatment:<br>Drug: Colchicine 0.6 mg<br>Colchicine treated subjects will take 0.6mg of drug 2x per day (1 time if kidney disease is present) for 14 days, then will take 0.6mg of drug 1x per day (or every other day if kidney disease is present) for 76 +/1 days.<br>Other Names: Colcrys, Gloperba, Lodoco, Mitigare<br>Drug: Control/Placebo group<br>Placebo treated subjects will take 0.6mg of placebo 2x per day (1 time if kidney disease is present) for 14 days, then will take 0.6mg of placebo1x per day (or every other day if kidney disease is present) for 76 +/1 days.	100 is chosen to provide preliminary data on efficacy and safety, which can inform larger, definitive trials.<br>Arms and Interventions: The parallel design with a placebo control is essential for assessing the efficacy of colchicine in this setting, considering the variability in the natural course of ADHF and the placebo effect.<br>Outcome Measures: The primary and secondary outcomes are chosen to evaluate both the biological effect of colchicine on inflammation (hsCRP and IL-6 levels) and its clinical impact (hospital readmission rates and NYHA functional class). This comprehensive approach will help to understand the potential benefits of colchicine in ADHF management.	Subsequently Control/Placebo group will be given placebo 1x/day for 76 +/- days (or once every other day if subject has kidney disease).<br>Interventions:<br>Drug: Control/Placebo group<br><br>Primary Outcome Measures:<br>Difference in the change in high sensitivity C-reactive protein (hsCRP) between colchicine arm and placebo arm in the first 72 hours of treatment [Time Frame: Baseline to 72 hours]<br>Change in plasma concentration of hsCRP between baseline and after 72 hours after treatment initiation, comparing colchicine arm vs placebo<br><br>Secondary Outcome Measures:<br>Difference in hsCRP area under curve between colchicine and placebo arm at 14 days [Time Frame: Baseline to 14 days]<br>Area under curve of hsCRP measurements obtained at baseline, 24h, 48h, 72h and 14 days, comparing colchicine vs placebo<br>Difference in change in plasma IL-6 concentration between colchicine arm and placebo arm in the first 72 hours of treatment [Time Frame: Baseline to 72 hours]<br>Change in plasma concentration of IL-6 between baseline and after 72 hours after treatment initiation, comparing colchicine arm vs placebo
12	Initial Trial Description	Inclusion Criteria:<br>Diagnosis of Chronic Bacterial Cystitis:	Inclusion Criteria:

	Official Title: Multicenter, Double-blind, Placebo-controlled, Randomized, Parallel-group Clinical Trial of the Efficacy and Safety of Raphamin in Prevention of the Recurrences of Chronic Bacterial Cystitis<br><br>Brief Summary: Clinical Trial of the Efficacy and Safety of Raphamin in Prevention of Recurrences of Chronic Bacterial Cystitis<br><br>Study Type: Interventional<br><br>Study Phase: Phase 3<br><br>Study Design: Allocation: Randomized<br>Interventional Model: Parallel Assignment<br>Masking: Quadruple (Participant Care Provider Investigator Outcomes Assessor)<br>Primary Purpose: Prevention<br><br>Conditions: Cystitis Chronic Cystitis Bacterial Recurrence of Chronic Bacterial Cystitis<br><br>Intervention / Treatment:<br>Drug: Raphamin. Tablet for oral use.<br>Other Names: MMH-407	Confirmed by urine culture with recurrent infections defined as ≥3 episodes in the past 12 months or ≥2 episodes in the last 6 months.<br>Age: 18 years and older. This ensures participants can provide informed consent and are likely to have a stable disease state.<br>Ability to Provide Informed Consent: Participants must be able to understand and willing to sign a written informed consent form.<br><br>Exclusion Criteria:<br>Pregnancy or Lactation: Due to unknown effects of Raphamin on fetal and infant health.<br>Severe Renal or Hepatic Dysfunction: As these conditions could affect the pharmacokinetics and safety profile of Raphamin.<br>Known Allergy to Raphamin or Similar Compounds: To prevent adverse allergic reactions.<br>Use of Antibiotics within 2 Weeks Prior to Enrollment: To ensure a stable baseline of bacterial flora.<br>Participation in Another Clinical Trial within the Last 30 Days: To avoid confounding effects from other investigational drugs or interventions.<br><br>Sex/Gender:<br>ALL: Chronic bacterial cystitis affects both males and females, though it is more common in females. Including all genders	Female patients aged 18 years and older.<br>Patients with a confirmed diagnosis of chronic bacterial cystitis.<br>Patients with exacerbation of chronic cystitis based on typical symptoms of exacerbation of the disease with a severity of 7 points or more in accordance wuth ACSS.<br>The first 48 hours from the onset of exacerbation) of chronic bacterial cystitis.<br>Patients who agreed to use an acceptable method of contraception during the study (for women of reproductive potential).<br>Availability of a signed patient information sheet and informed consent form for participation in the clinical trial.<br><br>Exclusion Criteria:<br><br>Presence of urolithiasis, urinary tract obstruction, urothelial cancer, pelvic organ prolapse, neurogenic bladder disorders at the time of examination.<br>Presence of malignant neoplasms of the urinary tract, ureter stone, more than 50 ml of residual urine in the bladder, confirmed by ultrasound examination.<br>Suspicion of bladder tuberculosis.<br>Presence of indications for hospitalization due to a serious condition, macrohematuria, complicated cystitis.<br>Decompensation of diabetes mellitus, immunodeficiency of any etiology, malignant cancer of any localization, severe circulatory insufficiency (cardiovascular disease with functional class IV according to the classification of the New York Heart Association, 1964).<br>Unstable angina pectoris or myocardial infarction within the previous 6 months.<br>Chronic kidney disease (classes C3-5 A3).<br>Hepatic impairment (Child-Pugh class C).<br>Patients who require medicinal products prohibited for use in this study.

	Drug: Placebo. Tablet for oral use.	ensures generalizability of trial results.<br><br>Ages:<br>18 Years and Older: This age range is chosen to include the adult population most commonly affected by chronic bacterial cystitis.<br><br>Arms and Interventions:<br>Raphamin Group: 150 participants will receive Raphamin tablets orally once daily.<br>Placebo Group: 150 participants will receive placebo tablets identical in appearance to Raphamin tablets, taken orally once daily.<br><br>Primary Outcome Measures:<br>Recurrence Rate of Chronic Bacterial Cystitis: Defined as the number of participants experiencing at least one recurrence of cystitis confirmed by urine culture over a 12-month period. This directly measures the efficacy of Raphamin in preventing recurrences.<br><br>Secondary Outcome Measures:<br>Time to First Recurrence: Time from the start of the study to the first recurrence of cystitis. This will provide information on how quickly Raphamin can impact recurrence rates.<br>Severity of Recurrences: Assessed by symptom scores (e.g., pain, urgency) during recurrences. This evaluates whether Raphamin affects the	Exacerbation or decompensation of chronic conditions affecting the patient's ability to participate in the clinical trial.<br>Malabsorption syndrome, including congenital or acquired lactase deficiency or other disaccharidase insufficiency, galactosemia.<br>Hypersensitivity to any component of the medicinal products used in the treatment.<br>Pregnancy, breastfeeding; childbirth less than 3 months prior to study enrollment, unwillingness to comply with contraceptive methods during the study.<br>The medications listed in the Prohibited Concomitant Medication section were administered within 4 weeks prior to enrollment.<br>Patients who, from the investigator's point of view, will not comply with the observation requirements of the study or comply with the administration of the study drug.<br>History of mental diseases, alcoholism, or drug abuse that, in the investigator's opinion, may interfere with the successful completion of trial procedures.<br>Participation in other clinical trials within 3 months prior to enrollment.<br>The patient belongs to the investigational site personnel directly involved in the study, closest relatives of the investigator. The closest relatives are defined as spouse, parents, children or siblings, regardless of whether they are natural or adopted.<br><br>The patient works for the company OOO "NPF "MATERIA MEDICA HOLDING", being an employee of the company, a temporary contract worker or an appointed official responsible for performing the trial, or their close relative.<br><br>Sex/Gender: Female<br><br>Ages: 18 Years and older (Adult, Older Adult)<br><br>Arms and Interventions:

		severity of symptoms during recurrences.<br>Quality of Life: Measured by a validated questionnaire (e.g., IC-Quality of Life Questionnaire) at baseline and every 3 months. This assesses the impact of Raphamin on overall well-being and quality of life.<br>Safety and Tolerability: Assessed by monitoring adverse events, vital signs, and laboratory tests throughout the study. Ensures that Raphamin is safe for long-term use.<br><br>Clinical Reasoning:<br>Inclusion and Exclusion Criteria: These criteria ensure that the study population is homogeneous in terms of disease state and not at undue risk from the intervention. They also help in minimizing confounding factors that could affect the study outcomes.<br>Sex/Gender and Age: Including all genders and focusing on adults ensures that the findings are applicable to the general population suffering from chronic bacterial cystitis.<br>Arms and Interventions: A placebo-controlled design is essential for assessing the true efficacy of Raphamin, and the parallel-group model allows for direct comparison between the intervention and control.<br>Outcome Measures: The primary and secondary outcomes are chosen to comprehensively	Experimental: Raphamin<br>Take orally, do not take with meals. Keep the tablets in the mouth until completely dissolved.<br><br>On the first day of treatment, 8 tablets are taken according to the following scheme: 1 tablet every 30 minutes in the first 2 hours (a total of 5 tablets in 2 hours), then during the same day another 1 tablet is taken 3 times at regular intervals. On the 2nd day and then take 1 tablet 3 times a day. The duration of treatment is 10 days.<br><br>Interventions:<br>Drug: Raphamin<br>Placebo Comparator: Placebo<br>Take orally, do not take with meals. Keep the tablets in the mouth until completely dissolved. Placebo is administered according to the Raphamin regimen for 10 days.<br>Interventions:<br>Drug: Placebo<br><br>Primary Outcome Measures:<br>Time to the first recurrence of chronic bacterial cystitis [Time Frame: 6 months]<br>Criteria for the diagnosis of chronic bacterial cystitis recurrence are typical symptoms of exacerbation (acute cystitis): frequent urination with small volumes of urine; urgent urination (a sudden and uncontrollable urge to pass urine; feeling pain or burning when passing urine; feeling of incomplete bladder emptying after urination; pain or uncomfortable pressure in the lower abdomen (above the pubis); possible presence of blood in the urine (especially towards the end of urination).<br><br>Criteria for the diagnosis of chronic bacterial cystitis are 2 and more recurrences during previous 6 months or 3 and more recurrences during previous 12 months.<br><br>Secondary Outcome Measures:

		evaluate the efficacy, impact on quality of life, and safety of Raphamin, providing a holistic view of its potential as a preventive treatment for chronic bacterial cystitis.	Average duration of episodes of recurrences of chronic bacterial cystitis [Time Frame: 6 months]<br>Average duration of episodes of recurrences of chronic bacterial cystitis after the start of study therapy at inclusion in the study, score 6 or less of typical symptoms of cystitis by the Acute Cystitis Symptom Score (according to the patient's diary).<br>Average number of recurrences of chronic bacterial cystitis [Time Frame: 6 months]<br>Average number of recurrences of chronic bacterial cystitis per patient during 6 months of follow-up.<br>Severity of chronic bacterial cystitis recurrences [Time Frame: 6 months]<br>Severity of chronic bacterial cystitis recurrences within 6 months of follow-up (as measured by the area under the curve for the total score of typical symptoms of cystitis on the Acute Cystitis Symptom Score (ACSS) during 10 days of treatment for each episode of recurrence).<br><br>ACSS is an 18-item self-reporting questionnaire for clinical diagnosis and follow-up of acute uncomplicated cystitis in women (min 0, max 18 points; 6 typical symptoms are included: urinary frequency, urgent urination, burning feeling when urinating, incomplete bladder empting after urination, pain or uncomfortable pressure in the lower abdomen, visible blood in urine; each symptom is estimated from 0 to 3 points).<br><br>Change in the severity of chronic bacterial cystitis recurrences [Time Frame: 6 months]<br>Change in the severity of chronic bacterial cystitis recurrences during 6 months of follow-up compared to baseline.<br>VAS score at the end of the follow-up period [Time Frame: 6 months]<br>Visual Analogue Scale (VAS) is used to assess the degree of satisfaction with the treatment. Treatment satisfaction scores are ranged from 0 (no satisfaction with treatment) to 10 points (very significant treatment

			effect), such that a higher score indicates greater treatment efficacy.<br>The Presence of Adverse Events (AEs). [Time Frame: 6 months]<br>The presence and nature of adverse events, their intensity (severity), causal relationship to the study drug, outcome. Based on medical records.<br>Changes in Vital Signs (Blood Pressure measured in mm Hg) [Time Frame: 6 months]<br>Changes in Blood Pressure at the end of treatment of the initial recurrence of chronic cystitis.<br>Changes in Vital Signs (Pulse Rate (Heart Rate) measured in beats per minute) [Time Frame: 6 months]<br>Changes in Heart Rate at the end of treatment of the initial recurrence of chronic cystitis.<br>Changes in Vital Signs (Respiration Rate (Breathing Rate) measured in breaths per minute) [Time Frame: 6 months]<br>Changes in Respiratory Rate at the end of treatment of the initial recurrence of chronic cystitis.<br><br>Based on medical records.<br><br>Proportion of patients with clinically significant laboratory abnormalities [Time Frame: 6 months]<br>Proportion of patients with clinically significant laboratory findings abnormalities at the end of treatment for initial recurrence of chronic cystitis.
13	Initial Trial Description<br>Official Title: A Randomized, Double-blind Study to Compare LNK01001 to Placebo in Adults With Rheumatoid Arthritis (RA) on a Stable Dose of csDMARDs Who Have an Inadequate Response or Intolerance to bDMARDs	Inclusion Criteria:<br>Adults aged 18 years or older.<br>Diagnosis of rheumatoid arthritis (RA) as defined by the 2010 American College of Rheumatology/European League Against Rheumatism classification criteria for RA.<br>Moderately to severely active RA despite treatment with a stable dose of one or more conventional synthetic	Inclusion Criteria:<br><br>Participants aged 18 and above.<br>Diagnosis of rheumatoid arthritis (RA) for ≥ 3 months.<br>≥ 6 swollen joints (based on 66 joint counts) and ≥ 6 tender joints (based on 68 joint counts) at Screening and baseline visit.<br>Erythrocyte sedimentation rate (ESR) ≥ 28mm/h or high-sensitivity C-Reactive Protein (hsCRP) ≥ ULN at Screening.<br>Participants have been receiving csDMARD therapy ≥ 3 months and on a stable dose for ≥ 4 weeks prior to the first dose of study drug.

	Brief Summary: This is a randomized, double-blind study comparing LNK01001 to placebo in Chinese participants with moderately to severely active rheumatoid arthritis who are on a stable dose of conventional synthetic disease-modifying anti-rheumatic drugs (csDMARDs) and have an inadequate response or Intolerance to biologic DMARDs(bDMARDs). The study objective of Period 1 (Day 1 to Week 24) is to compare the safety and efficacy of LNK01001 12 mg twice daily (BID) versus placebo for the treatment of signs and symptoms of participants with moderately to severely active rheumatoid arthritis (RA) who are on a stable dose of csDMARDs and had an inadequate response to or intolerance to at least 1 bDMARD. The study objective of Period 2 (Week 24 to Week 76) is to evaluate the long-term safety, tolerability, and efficacy of LNK01001 12 mg BID in participants with RA who completed Period 1.<br><br>Study Type: Interventional	disease-modifying anti-rheumatic drugs (csDMARDs) for at least 12 weeks prior to screening.<br>Inadequate response or intolerance to at least one biologic DMARD (bDMARD).<br>Stable doses of csDMARDs for at least 4 weeks prior to randomization.<br>Ability to provide informed consent.<br><br>Exclusion Criteria:<br>Previous treatment with LNK01001.<br>History of hypersensitivity to any component of the study drug or placebo.<br>Pregnant or breastfeeding women.<br>Active infections or history of recurrent infections.<br>History of malignancy within the last 5 years (except for treated and cured basal or squamous cell carcinoma of the skin or cervical carcinoma in situ).<br>Severe comorbidities, such as uncontrolled cardiovascular, pulmonary, hepatic, renal, or neurological diseases.<br>Receipt of live vaccines within 6 weeks prior to randomization.<br><br>Sex/Gender: ALL<br>Ages: Adults aged 18 years or older.<br><br>Arms and Interventions:<br>Participant Group/Arm 1: LNK01001 Treatment<br>Intervention/Treatment: Drug: LNK01001,	Have an inadequate response to ≥ 1 bDMARD.<br><br>Exclusion Criteria:<br><br>Subjects who were prior exposure to Janus Kinase (JAK) inhibitor (including but not limited to tofacitinib, baricitinib, and filgotinib) and have evidence showing an inadequate response or intolerance.<br>Subjects who received intra-articular, intramuscular, intravenous, trigger point or tender point, intracapsular, or intra-tendon injections of glucocorticoids within 4 weeks before randomization.<br>Current diagnosis of systemic inflammatory disease other than RA.<br>History of malignancy or current diagnosis of malignancy within 5 years before screening visit.<br>Uncontrolled diabetes, hypertension, kidney disease, liver disease, severe heart disease.Exclusion Criteria:<br><br>Subjects who were prior exposure to Janus Kinase (JAK) inhibitor (including but not limited to tofacitinib, baricitinib, and filgotinib) and have evidence showing an inadequate response or intolerance.<br>Subjects who received intra-articular, intramuscular, intravenous, trigger point or tender point, intracapsular, or intra-tendon injections of glucocorticoids within 4 weeks before randomization.<br>Current diagnosis of systemic inflammatory disease other than RA.<br>History of malignancy or current diagnosis of malignancy within 5 years before screening visit.<br>Uncontrolled diabetes, hypertension, kidney disease, liver disease, severe heart disease.<br><br>Sex/Gender: All<br><br>Ages: 18 Years and older (Adult, Older Adult)<br><br>Arms and Interventions:<br>Experimental: LNK01001 12 mg

	Study Phase: Phase 3<br><br>Study Design:<br>Allocation: Randomized<br>Interventional Model: Parallel Assignment<br>Masking: Quadruple (Participant Care Provider Investigator Outcomes Assessor)<br>Primary Purpose: Treatment<br><br>Conditions:<br>Rheumatoid Arthritis<br><br>Intervention / Treatment:<br>Drug: LNK01001 Capsule; Oral<br>Drug: Placebo Capsule; Oral	Capsule; Oral, 12 mg twice daily (BID).<br>Participant Group/Arm 2: Placebo Comparator Intervention/Treatment: Drug: Placebo, Capsule; Oral, matching placebo BID.<br><br>Primary Outcome Measures:<br>Outcome Measure: Change in Disease Activity Score 28 (DAS28) using C-reactive protein (CRP).<br>Measure Description: The DAS28-CRP is a measure of RA disease activity that includes swollen and tender joint counts, CRP levels, and the patient's global assessment of health. A decrease in DAS28-CRP indicates improvement.<br>Time Frame: From baseline to Week 24.<br><br>Secondary Outcome Measures:<br>Outcome Measure: Proportion of participants achieving American College of Rheumatology 20 (ACR20) response.<br>Measure Description: ACR20 is a 20% improvement in tender or swollen joint counts and a 20% improvement in three of the following five criteria: patient's assessment of pain, patient's global assessment of disease activity, physician's global assessment of disease activity, participant's assessment of physical function, and levels of an acute-phase reactant.<br>Time Frame: Week 24.	Period 1: Participants receive LNK01001 12 mg twice daily for 24 weeks. Period 2: Participants will continue on LNK01001 12 mg twice daily from Week 24 to Week 76.<br>Interventions:<br>Drug: LNK01001<br>Placebo Comparator: Placebo / LNK01001 12 mg<br>Period 1: Participants receive a placebo twice daily for 24 weeks.<br>Period 2: Participants will switch to receive LNK01001 12 mg twice daily from Week 24 to Week 76.<br>Interventions:<br>Drug: Placebo<br><br>Primary Outcome Measures:<br>Change from Baseline in Disease Activity Score 28 (DAS28) (CRP) at week 24 [Time Frame: Baseline and Week 24]<br>The DAS28 is a composite index used to assess rheumatoid arthritis disease activity, calculated based on the tender joint count (out of 28 evaluated joints), swollen joint count (out of 28 evaluated joints), Patient's Global Assessment of Disease Activity (0-100 mm), and hsCRP (in mg/L). Scores on the DAS28 range from 0 to approximately 10, where higher scores indicate more disease activity.<br>Percentage of Participants with an American College of Rheumatology 50% (ACR50) Response at week 24 [Time Frame: Baseline and Week 24]<br>Participants who met the following 3 conditions for improvement from baseline were classified as meeting the ACR50 response criteria:<br><br>1.50% improvement in 68-tender joint count; 2.50% improvement in 66-swollen joint count; and 3.50% improvement in at least 3 of the 5 following parameters:<br><br>Physician global assessment of disease activity<br>Patient global assessment of disease activity<br>Patient assessment of pain

		Outcome Measure: Change in Health Assessment Questionnaire Disability Index (HAQ-DI) score.<br>Measure Description: The HAQ-DI assesses a participant's level of functional ability. A decrease in HAQ-DI score indicates improvement.<br>Time Frame: From baseline to Week 24.<br><br>Clinical Reasoning:<br>Inclusion Criteria: The inclusion criteria are designed to select adults with moderately to severely active RA who have not responded adequately to csDMARDs and bDMARDs, ensuring the study population is representative of the target patient population for LNK01001.<br>Exclusion Criteria: The exclusion criteria aim to ensure participant safety by excluding individuals with conditions that could increase the risk of adverse events or interfere with the interpretation of study results.<br>Sex/Gender and Ages: Including all adults regardless of sex or gender ensures the study results are generalizable to the broader RA patient population.<br>Arms and Interventions: A placebo comparator is essential for assessing the efficacy and safety of LNK01001 in a double-blind manner, ensuring the reliability of study results.	Health Assessment Questionnaire - Disability Index (HAQ-DI)<br>High-sensitivity C-reactive protein (hsCRP).<br>Change from Baseline in Health Assessment Questionnaire - Disability Index (HAQ-DI) at all visits. [Time Frame: Baseline to Week 76.]<br>The Health Assessment Questionnaire - Disability Index is a patient-reported questionnaire that measures the degree of difficulty a person has in accomplishing tasks in 8 functional areas (dressing, arising, eating, walking, hygiene, reaching, gripping, and errands and chores) over the past week. Participants assessed their ability to do each task on a scale from 0 (without any difficulty) to 3 (unable to do). Scores were averaged to provide an overall score ranging from 0 to 3, where 0 represents no disability and 3 represents very severe, high-dependency disability. A negative change from Baseline in the overall score indicates improvement.<br>Percentage of Participants with an American College of Rheumatology 70% (ACR70) Response at all visits. [Time Frame: Baseline to Week 76.]<br>Participants who met the following 3 conditions for improvement from baseline were classified as meeting the ACR70 response criteria:<br><br>70% improvement in 68-tender joint count;<br>70% improvement in 66-swollen joint count; and 3.70% improvement in at least 3 of the 5 following parameters:<br><br>Physician global assessment of disease activity<br>Patient global assessment of disease activity<br>Patient assessment of pain<br>Health Assessment Questionnaire - Disability Index (HAQ-DI)<br>High-sensitivity C-reactive protein (hsCRP).<br>Percentage of Participants Achieving Low Disease Activity (LDA) Based on DAS28(CRP) at all visits. [Time Frame: Baseline to Week 76.]

		Primary and Secondary Outcome Measures: The chosen outcome measures are standard in RA clinical trials and will provide comprehensive data on the efficacy and safety of LNK01001, as well as its impact on disease activity and patient quality of life.	Low disease activity. was defined as a DAS28 score less than or equal to 3.2.The DAS28 is a composite index used to assess rheumatoid arthritis disease activity, calculated based on the tender joint count (out of 28 evaluated joints), swollen joint count (out of 28 evaluated joints), Patient's Global Assessment of Disease Activity (0-100 mm), and hsCRP (in mg/L). Scores on the DAS28 range from 0 to approximately 10, where higher scores indicate more disease activity.<br>Percentage of Participants Achieving Clinical Remission (CR) Based on DAS28(CRP) at all visits [Time Frame: Baseline to Week 76.]<br>Clinical remission was defined as a DAS28 (CRP) score less than 2.6.The DAS28 is a composite index used to assess rheumatoid arthritis disease activity, calculated based on the tender joint count (out of 28 evaluated joints), swollen joint count (out of 28 evaluated joints), Patient's Global Assessment of Disease Activity (0-100 mm), and hsCRP (in mg/L). Scores on the DAS28 range from 0 to approximately 10, where higher scores indicate more disease activity.<br>Percentage of Participants with an American College of Rheumatology 20% (ACR20) Response at all visits (except week 24). [Time Frame: Baseline to Week 76 (except week 24).]<br>Participants who met the following 3 conditions for improvement from baseline were classified as meeting the ACR20 response criteria:<br><br>1.20% improvement in 68-tender joint count; 2. 20% improvement in 66-swollen joint count; and 3. 20% improvement in at least 3 of the 5 following parameters:<br><br>Physician global assessment of disease activity<br>Patient global assessment of disease activity<br>Patient assessment of pain

			Health Assessment Questionnaire - Disability Index (HAQ-DI)<br>High-sensitivity C-reactive protein (hsCRP).<br>Change from Baseline in the Severity of Morning Stiffness at all visits. [Time Frame: Baseline to Week 76.]<br>Morning stiffness severity was determined by the Patient's Assessment of Severity and Duration of Morning Stiffness questionnaire. Participants rated the severity of morning stiffness on awakening over the past 7 days on a scale from 0 (No morning stiffness) to 10 (Worst possible morning stiffness).<br><br>Secondary Outcome Measures:<br>Nil
14	Initial Trial Description<br>Official Title: Comparison of Postoperative Pain Score Between Perioperative Intravenous Ketamine and Placebo in Patients Undergoing Unilateral Total Knee Arthroplasty Under General Anesthesia, A Prospective Randomized Controlled Trial Study<br><br>Brief Summary: The goal of this clinical trial is to compare postoperative pain score between perioperative intravenous ketamine and placebo in patients undergoing unilateral total knee arthroplasty under general anesthesia. The main question[s] it aims to answer are: Can perioperative intravenous ketamine reduce postoperative pain score during rest and movement at 0,	Inclusion Criteria:<br>Patients aged 18 years and older.<br>Patients scheduled for unilateral total knee arthroplasty (TKA) due to osteoarthritis.<br>Patients able to provide informed consent.<br>Patients with ASA (American Society of Anesthesiologists) physical status I-III.<br><br>Exclusion Criteria:<br>Patients with a known allergy or contraindication to ketamine.<br>Patients with a history of chronic opioid use or substance abuse.<br>Patients with severe psychiatric disorders, including schizophrenia or bipolar disorder.<br>Patients with severe cardiovascular disease, including uncontrolled hypertension or recent myocardial infarction.<br>Pregnant or breastfeeding women.<br>Patients with renal or hepatic impairment.	Inclusion Criteria:<br><br>A patient undergo unilateral total knee arthroplasty under General anesthesia at Chomthong Hospital Chiang Mai During 2024 to December 2025<br>Age is more than 18 years old<br>ASA (American society of anesthesiologist) status are I,I,III<br>A patient can cooperate in research<br><br>Exclusion Criteria:<br><br>Patients allergic to local anesthetic agent<br>Patient who has contraindication to use ketamine<br>Patients has coagulopathy.<br>Injection site is infected.<br>Patients with chronic pain received painkillers for more than 3 months.<br>The patient has a history of opioid use.<br>The patient has a history of alcohol dependence.<br>Patients allergic to fentanyl or morphine.<br>Patient who has unstable cardiovascular disease<br>Patient who has increase intracranial pressure and high ocular pressure<br>Pregnancy<br>The patient who has communication problems cannot describe the level of pain

	2, 4, 8, 12, 18, 24 hours better than no administration of Ketamine in patients undergoing Unilateral total knee arthroplasty under general anesthesia? Can perioperative intravenous ketamine reduce morphine consumption in postoperative 24 hours, length of hospital stay, first time to receive opioid and side effect or complication from ketamine and opioid better than no administration of Ketamine in patients undergoing Unilateral total knee arthroplasty under general anesthesia? Participants will receive intravenous ketamine intraoperative TKA and comparison group will receive placebo that is normal saline. Researchers will compare perioperative intravenous ketamine and placebo to see postoperative pain score, morphine consumption in postoperative 24 hours, length of hospital stay, first time to receive opioid and side effect or complication from ketamine and opioid.<br><br>Study Type: Interventional<br><br>Study Phase: Phase 4	Patients who have received any investigational drug within the last 30 days.<br><br>Sex/Gender: ALL<br>The study will include both male and female participants to ensure the findings are applicable across genders.<br><br>Ages:<br>Participants aged 18 years and older. This age range is selected to focus on adults who are most likely to undergo TKA due to osteoarthritis.<br><br>Arms and Interventions:<br>Ketamine Group (Intervention): Participants in this group will receive intravenous ketamine at a dose of 0.5 mg/kg at induction of anesthesia, followed by a continuous infusion of 0.25 mg/kg/hr until the end of surgery.<br>Placebo Group (Control): Participants in this group will receive an equivalent volume of normal saline at the same time points as the ketamine group.<br><br>Primary Outcome Measures:<br>Postoperative Pain Score: Measured using the Visual Analog Scale (VAS) at rest and during movement at 0, 2, 4, 8, 12, 18, and 24 hours postoperatively. This measure will help determine the efficacy of ketamine in reducing acute postoperative pain.	The patient refused to participate in the study.<br><br>Sex/Gender: All<br><br>Ages: 18 Years to 100 Years (Adult, Older Adult)<br><br>Arms and Interventions:<br>Experimental: ketamine group<br>Participant will receive intraoperative ketamine during total knee arthroplasty by Ketamine 0.5mg /kg loading then 0.25 mg/kg/hours until end of surgery<br>Interventions:<br>Drug: Ketamine group<br>Placebo Comparator: Placebo group<br>Participant will receive intraoperative normal saline during total knee arthroplasty<br>Interventions:<br>Drug: Placebo group<br><br>Primary Outcome Measures:<br>Postoperative pain score at rest and movement [Time Frame: post operative time at 0 ,2 ,4 ,8 ,12 ,18 ,24 hours]<br>pain score at rest and movement by numeric rating scale 0-10<br><br>Secondary Outcome Measures:<br>First time to receive intravenous analgesic drug [Time Frame: First time to receive intravenous analgesic drug (From finished anesthetic time until First time to receive intravenous analgesic drug(minute)]<br>First time to receive intravenous analgesic drug after operation was finished<br>Morphine consumption [Time Frame: post operative time at 0 ,2 ,4 ,8 ,12 ,18 ,24 hours]<br>Post operative morphine consumption or morphine equivalent (milligram)<br>Length of hospital stay [Time Frame: Time that patient was admited to hospital until patient was discharge from hospital.]<br>Length of hospital stay : When participant admit to discharge

	Study Design:<br>Allocation:<br>Randomized<br>Interventional Model:<br>Parallel Assignment<br>Masking: Quadruple<br>(Participant Care<br>Provider Investigator<br>Outcomes Assessor)<br>Primary Purpose:<br>Treatment<br><br>Conditions: Total<br>Knee Arthropathy<br>Knee Osteoarthritis<br><br>Intervention /<br>Treatment:<br>Drug: Ketamine<br>group. Participants<br>will receive<br>intraoperative<br>ketamine during total<br>knee arthroplasty<br>Drug: Placebo group.<br>Participants will<br>receive intraoperative<br>normal saline during<br>total knee<br>arthroplasty	Secondary Outcome<br>Measures:<br>Morphine Consumption:<br>Total morphine<br>consumption in the first<br>24 hours<br>postoperatively. This will<br>help assess if ketamine<br>reduces the need for<br>opioid analgesia.<br>Length of Hospital Stay:<br>Measured in days from<br>the day of surgery until<br>discharge. This will help<br>evaluate if ketamine<br>impacts the recovery<br>process.<br>Time to First Opioid<br>Request: Time from the<br>end of surgery to the first<br>request for opioid<br>analgesia. This will help<br>assess the analgesic<br>efficacy of ketamine in<br>the immediate<br>postoperative period.<br>Side Effects and<br>Complications:<br>Incidence of side effects<br>or complications related<br>to ketamine and opioids,<br>including nausea,<br>vomiting, hallucinations,<br>and respiratory<br>depression. This will<br>help evaluate the safety<br>profile of ketamine in this<br>setting.<br><br>Clinical Reasoning:<br>Inclusion and Exclusion<br>Criteria: These criteria<br>are designed to select a<br>homogeneous group of<br>patients undergoing TKA<br>due to osteoarthritis,<br>who are otherwise<br>healthy enough to<br>participate in a clinical<br>trial. Excluding patients<br>with conditions that<br>could interfere with the<br>study outcomes or pose<br>a risk ensures the safety	side effect and complication from<br>ketamine and opioid [Time Frame:<br>post operative time within 24 hours]<br>Number of Participants with side<br>effect form Ketamine and opioid:<br>Hallucination, Nightmare, Arrythmia ,<br>Nausea vomiting respiratory distress<br>,Pruritus within 24 hours after<br>operation. Do you have hallucination<br>or nightmare? : yes no<br><br>Nausea vomiting : - Severity of<br>nausea 4 scale :<br><br>0 = No<br><br>=minimal nausea and vomiting don't<br>need medication<br>= nausea and vomiting participant<br>was improved when received<br>medication 3= nausea and vomiting<br>participant wasn't improved when<br>received medication Pruritus score<br>0= no<br>1= minimal 2= severe and need<br>medication respiratory distress :<br>sedation scale 0 = awake<br><br>minimal sedation : response to verbal<br>conversation<br>moderated sedation : sleeping but<br>easy to response to verbal<br>conversation<br>deep sedation : difficult to response<br>to

		of participants and the integrity of the data.<br>Sex/Gender and Ages: Including all genders and adults 18 years and older ensures the study results are generalizable to the population most likely to undergo TKA.<br>Arms and Interventions: The use of a placebo control and ketamine intervention allows for a clear comparison of the effects of ketamine on postoperative pain and opioid consumption. The dosing regimen for ketamine is based on previous studies showing efficacy and safety in perioperative pain management.<br>Primary and Secondary Outcome Measures: These measures are selected to comprehensively assess the efficacy and safety of ketamine in reducing postoperative pain, opioid consumption, and related outcomes. The time frames are chosen to capture immediate and short-term postoperative periods, which are critical for patient recovery and hospital discharge planning.	
15	Initial Trial Description<br>Official Title: Multicenter, Double-blind, Placebo-controlled, Randomized, Parallel-group Clinical Trial to Evaluate the Efficacy and Safety of Raphamin in Combined Treatment of Community-acquired Pneumonia	Inclusion Criteria:<br>Diagnosis of Community-acquired Pneumonia (CAP): Confirmed by clinical assessment and radiological evidence (chest X-ray or CT scan) within 48 hours prior to enrollment.<br>Age: 18 years and older. This age range is chosen to include the adult population most	Inclusion Criteria:<br><br>Male and female patients aged 18-65 years.<br>The diagnosis of community-acquired pneumonia presupposes that the patient has focal infiltration of the lung tissue, confirmed by imaging (X-ray/CT), and the presence of at least two clinical symptoms:<br><br>a) acute fever at the onset of the disease (axillary temperature ≥ 38.0°C); b) wet cough; c) physical

	Brief Summary: Multicenter double-blind placebo-controlled randomized in parallel groups clinical trial.<br><br>Study Type: Interventional<br><br>Study Phase: Phase 3<br><br>Study Design: Allocation: Randomized Interventional Model: Parallel Assignment Masking: Quadruple (Participant Care Provider Investigator Outcomes Assessor) Primary Purpose: Treatment<br><br>Conditions: Community-acquired Pneumonia Pneumonia<br><br>Intervention / Treatment:<br>Drug: Raphamin. Tablet for oral use.<br>Other Names: MMH-407<br>Drug: Placebo. Tablet for oral use.	likely to be affected by CAP and capable of providing informed consent.<br>Ability to provide informed consent: Participants must be able to understand and willing to sign a written informed consent form.<br>Symptom Duration: Patients presenting with symptoms of pneumonia (e.g., cough, fever, dyspnea) for no more than 7 days before enrollment. This ensures that the study targets acute cases of CAP.<br>Severity of Illness: Patients with a Pneumonia Severity Index (PSI) score or CURB-65 score indicating moderate risk. This criterion aims to include patients who are most likely to benefit from the intervention without being at an extreme risk of mortality.<br><br>Exclusion Criteria:<br>Hospital-acquired Pneumonia (HAP): Patients with pneumonia symptoms that started more than 48 hours after hospital admission.<br>Immunocompromised Status: Patients with known HIV/AIDS, organ transplant recipients, or those on chronic corticosteroids or immunosuppressive therapy.<br>Chronic Respiratory Diseases: Patients with chronic obstructive pulmonary disease (COPD) or asthma requiring hospitalization in the past year.	signs (crepitus/wheezing, bronchial breathing sounds, local dullness to percussion); d) leukocytosis >10×10^9/L and/or shift of band neutrophils (>10%).<br><br>SpO2 ≥95% according to pulse oximetry.<br>Overall CRB-65 score "0" points.<br>Patients willing to use reliable methods of contraception during the study (men and women of reproductive potential).<br>Patients who have signed the patient information sheet and informed consent.<br><br>Exclusion Criteria:<br><br>Any indications for patient hospitalization.<br>Suspected infiltrative pulmonary tuberculosis.<br>Medical history of/suspected malignant neoplasm of any location, including primary lung cancer and metastases to the lung parenchyma.<br>Suspected pulmonary embolism and pulmonary infarction.<br>Medical history of chronic obstructive pulmonary disease, bronchial asthma, chronic respiratory failure.<br>Medical history of immune system diseases: systemic vasculitis, lupus pneumonitis, allergic bronchopulmonary aspergillosis, obliterating bronchiolitis, idiopathic pulmonary fibrosis, eosinophilic pneumonia, bronchocentric granulomatosis.<br>Chronic heart failure III or IV FC (according to the New York Heart Association classification, 1964).<br>Suspicion of drug-induced (toxic) pneumopathy, aspiration of foreign body, sarcoidosis, pulmonary alveolar proteinosis, lipoid pneumonia, rounded atelectasis.<br>Exacerbated or decompensated chronic diseases, including diabetes mellitus, affecting a patient's ability to participate in the clinical trial.<br>Prior diagnosis of immunodeficiency of any etiology.

		Pregnancy or Lactation: Due to unknown effects of the drug on fetuses or infants.<br>Allergy to Raphamin or similar compounds: Known hypersensitivity to the investigational drug or its excipients.<br>Severe Comorbidities: End-stage renal disease, liver failure, or any terminal illness with a life expectancy of less than 6 months.<br><br>Sex/Gender: ALL<br>The trial will include both male and female participants to ensure the findings are applicable across genders.<br><br>Ages:<br>Participants aged 18 years and older. This encompasses the adult population most at risk for CAP and excludes pediatric cases which may have different treatment responses and safety profiles.<br><br>Arms and Interventions:<br>Experimental Arm: Participants will receive Raphamin tablets orally, dosage as per the protocol, for a duration specified by the study design.<br>Placebo Arm: Participants will receive placebo tablets identical in appearance to Raphamin, administered orally for the same duration as the experimental arm.<br><br>Primary Outcome Measures:	Patients having unstable angina pectoris or myocardial infarction in the previous 6 months.<br>Prior history of chronic kidney disease (categories C3-5 A3).<br>Prior history of hepatic failure (Child-Pugh class C).<br>Any surgery within the previous 3 months.<br>Patients who received any medicine specified in the section "Prohibited concomitant treatment" within 3 months preceding the inclusion in this study or who require the use of medicines that are prohibited within this trial.<br>Malabsorption syndrome, including congenital or acquired lactase deficiency (or any other disaccharidase deficiency) and galactosemia.<br>Hypersensitivity to any of the components of medications used in the treatment.<br>Pregnancy, breast-feeding; childbirth less than 3 months prior to the inclusion in the trial, unwillingness to use contraceptive methods during the trial.<br>Patients who, from the investigator's point of view, will not comply with study monitoring requirements or with study drug administration requirements.<br>Prior history of a psychiatric disorder, alcoholism or drug abuse, which in the opinion of the investigator may compromise compliance with the study protocol.<br>Participation in other clinical studies within 3 month prior to enrollment in the study.<br>Patients who are related to any of the on-site research personnel directly involved in the conduct of the trial or are an immediate relative of the study investigator. 'Immediate relative' means husband, wife, parents, children, or siblings, regardless of whether they are natural or adopted.<br>Patients who work for OOO "NPF "MATERIA MEDICA HOLDING" (i.e. the company's employees, temporary contract employees, persons designated officials responsible for

		Clinical Improvement: Defined as resolution or significant improvement in baseline symptoms of pneumonia (e.g., cough, fever, dyspnea) within 7 days of treatment initiation. This outcome measure is chosen as it directly reflects the efficacy of Raphamin in treating CAP.<br>Radiological Resolution: Improvement in chest X-ray or CT findings consistent with pneumonia resolution at 14 days post-treatment initiation. This provides an objective measure of treatment efficacy.<br><br>Secondary Outcome Measures:<br>Time to Clinical Stability: Time from treatment initiation to achieving clinical stability based on vital signs and clinical judgment, measured up to 14 days.<br>Hospitalization Duration: Length of hospital stay, if applicable, from the date of treatment initiation to discharge, measured up to 30 days.<br>Adverse Events (AEs): Incidence and severity of AEs and serious adverse events (SAEs) from the time of first dose until 30 days post-treatment.<br><br>Clinical Reasoning:<br>Inclusion and Exclusion Criteria: Designed to select a population with confirmed CAP of moderate severity, excluding those with conditions that could confound treatment	carrying out the research or any immediate relatives of the aforementioned).<br><br>Sex/Gender: All<br><br>Ages: 18 Years to 65 Years (Adult, Older Adult)<br><br>Arms and Interventions:<br>Experimental: Raphamin<br>Oral administration, without food. The tablet should be held in the mouth until complete dissolution.<br><br>On the first day 8 tablets are administered using the following scheme: 1 tablet every 30 minutes in the first 2 hours (5 tablets in total within 2 hours), followed by three more tablets at regular intervals during the rest of the day. From day 2 onwards, 1 tablet taken 3 times daily. The treatment period is 7 days.<br><br>Interventions:<br>Drug: Raphamin<br>Placebo Comparator: Placebo<br>Oral administration, without food. The tablet should be held in the mouth until complete dissolution.<br><br>Placebo is administered according to the Raphamin regimen for 7 days.<br><br>Interventions:<br>Drug: Placebo<br><br>Primary Outcome Measures:<br>Percentage of patients with clinical cure at the time of cure (TOC) visit [Time Frame: On 8 days]<br>Percentage of patients with clinical cure at the TOC visit (on day 8 after randomization based on the physician's assessment of clinical symptoms).<br><br>Secondary Outcome Measures:<br>Average duration of antibacterial therapy [Time Frame: 15 days]<br>Average duration of antibacterial therapy in two groups

		outcomes or pose safety risks.<br>Sex/Gender and Ages: Including all genders and focusing on adults ensures broad applicability of the results while minimizing risks associated with pediatric or elderly populations.<br>Arms and Interventions: A placebo-controlled design allows for the assessment of Raphamin's efficacy over standard care without the intervention.<br>Outcome Measures: Primary outcomes focus on clinical and radiological resolution to assess efficacy comprehensively. Secondary outcomes provide additional safety and efficacy data, including the impact on healthcare resources and patient quality of life	
16	Initial Trial Description<br>Official Title: A Phase 3 Randomized, Open-label Induction, Double-blind Maintenance, Parallel-group, Multicenter Protocol to Evaluate the Efficacy, Safety, and Pharmacokinetics of Guselkumab in Pediatric Participants With Moderately to Severely Active Ulcerative Colitis<br><br>Brief Summary: The purpose of this study is to evaluate the efficacy of guselkumab in pediatric participants	Inclusion Criteria:<br>Pediatric participants aged 6 to 17 years, inclusive, at the time of consent.<br>Diagnosed with moderately to severely active ulcerative colitis at least 3 months prior to screening, confirmed by endoscopy.<br>Previous inadequate response, loss of response, or intolerance to conventional therapy or biologics.<br>Able to provide assent (if applicable) and informed consent by a parent or legal guardian.<br><br>Exclusion Criteria:<br>Diagnosis of Crohn's disease or indeterminate colitis.	Inclusion Criteria:<br><br>Weight greater than or equal to (>=) 10 kilogram (kg) at the time of consent for screening<br>Documented diagnosis of ulcerative colitis (UC). A biopsy report supporting the diagnosis must be available in the source documents<br>Moderately to severely active UC, defined by a baseline modified Mayo (without physician's global assessment) score of 5 through 9 inclusive, with a screening Mayo endoscopy subscore >= 2 as determined by a central review of the video of the endoscopy, and a baseline Mayo rectal bleeding subscore >=1<br>Medically stable on the basis of physical examination, medical history, and vital signs, performed at screening. Any abnormalities must be consistent with the underlying illness in the study population and this

	with moderately to severely active ulcerative colitis at the end of maintenance therapy among participants who were induction responders.<br><br>Study Type: Interventional<br><br>Study Phase: Phase 3<br><br>Study Design:<br>Allocation: Randomized<br>Interventional Model: Parallel Assignment<br>Masking: Double (Participant Investigator)<br>Primary Purpose: Treatment<br><br>Conditions:<br>Colitis, Ulcerative<br><br>Intervention / Treatment:<br>Drug: Guselkumab. Guselkumab will be administered either intravenously or subcutaneously.<br>Other Names:<br>CNTO1959<br>TREMFYA<br>Drug: Matching Placebo. Week 12 induction responders will be administered placebo (matching guselkumab up to Week 56) SC at protocol specified time points to maintain the blind.	Presence of a stoma or ileoanal pouch.<br>History of colectomy.<br>Severe or fulminant colitis requiring immediate surgical intervention.<br>History of malignancy or lymphoproliferative disease.<br>Active tuberculosis or other severe infections.<br>Previous treatment with guselkumab or participation in another clinical trial involving an investigational product within 30 days or 5 half-lives of the investigational product, whichever is longer, prior to screening.<br><br>Sex/Gender:<br>ALL (Both male and female participants will be included to ensure the study results are generalizable across the pediatric population with ulcerative colitis.)<br><br>Ages:<br>6 to 17 years<br><br>Arms and Interventions:<br>Participant Group/Arm 1: Guselkumab Treatment<br>Intervention/Treatment: Guselkumab administered either intravenously or subcutaneously as per the dosing schedule outlined in the protocol.<br><br>Participant Group/Arm 2: Placebo Comparator<br>Intervention/Treatment: Matching placebo administered subcutaneously at protocol specified time points to maintain the blind.	determination must be recorded in the participant's source documents and acknowledged by the investigator<br>Participants must have had an inadequate response and/or intolerance to biologic therapy and/or conventional therapies or be dependent upon corticosteroids<br><br>Exclusion Criteria:<br><br>Have UC limited to the rectum only or to less than (<) 20 centimeter of the colon<br>Presence of a stoma<br>Has had any kind of bowel resection within 6 months or any other intra-abdominal surgery within 3 months of baseline<br>Have severe colitis or have evidence of Crohn's Disease (CD)<br><br>Sex/Gender: All<br><br>Ages: 2 Years to 17 Years (Child)<br><br>Arms and Interventions:<br>Experimental: Open-label Induction Phase: Guselkumab Intravenously (IV)<br>Participants will receive a guselkumab dose IV based on their body weight (BW) during the 12-week open-label induction phase.<br>Interventions:<br>Drug: Guselkumab<br>Experimental: Open-label Induction Phase: Guselkumab Subcutaneously (SC)<br>Participants will receive a guselkumab dose SC based on their BW during the 12-week open-label induction phase.<br>Interventions:<br>Drug: Guselkumab<br>Experimental: Double-blind Maintenance Phase: Guselkumab SC or Guselkumab SC and Placebo SC<br>At the end of the induction phase, Week 12 responders will be randomized into the double-blind maintenance phase to receive a guselkumab dose SC based on their BW or a guselkumab dose SC based

		Primary Outcome Measures:<br>Outcome Measure: Proportion of participants achieving clinical remission<br>Measure Description: Clinical remission defined as a Pediatric Ulcerative Colitis Activity Index (PUCAI) score of ≤10.<br>Time Frame: Week 56<br><br>Secondary Outcome Measures:<br>Outcome Measure: Proportion of participants achieving endoscopic improvement<br>Measure Description: Endoscopic improvement defined as a Mayo endoscopic subscore of 0 or 1.<br>Time Frame: Week 56<br><br>Outcome Measure: Change in quality of life scores from baseline<br>Measure Description: Assessed by the Pediatric Ulcerative Colitis Quality of Life (PUCAI-QoL) questionnaire.<br>Time Frame: Baseline to Week 56<br><br>Clinical Reasoning:<br><br>Inclusion Criteria: Focused on pediatric participants with a confirmed diagnosis to ensure the study population is representative of those with active disease who may benefit from the intervention. Prior inadequate response to therapies ensures	on their BW and placebo SC up to Week 56.<br>Interventions:<br>Drug: Guselkumab<br>Drug: Matching Placebo<br>Experimental: Open-label Maintenance Phase: Guselkumab SC Week 12 non-responders will enter an open-label maintenance phase to receive guselkumab SC dosing regimen based on their body weight up to Week 56.<br>Interventions:<br>Drug: Guselkumab<br><br>Primary Outcome Measures:<br>Percentage of Participants with Clinical Remission at Week 56 [Time Frame: Week 56]<br>Percentage of participants with clinical remission as assessed by modified Mayo score at Week 56 among participants who were induction responders will be reported. Clinical remission per modified Mayo score is defined as a stool frequency subscore of 0 or 1, a rectal bleeding subscore of 0, and an endoscopy subscore of 0 or 1 with no friability present on the endoscopy, where the stool frequency subscore has not increased from induction baseline.<br><br>Secondary Outcome Measures:<br>Percentage of Participants with Clinical Remission at Week 12 [Time Frame: Week 12]<br>Percentage of participants with clinical remission at Week 12 as assessed by modified Mayo score will be reported. Clinical remission per modified Mayo score is defined as a stool frequency subscore of 0 or 1, a rectal bleeding subscore of 0, and an endoscopy subscore of 0 or 1 with no friability present on the endoscopy, where the stool frequency subscore has not increased from induction baseline.<br>Percentage of Participants With Pediatric Ulcerative Colitis Activity Index (PUCAI) Remission at Week 12 [Time Frame: Week 12]<br>Percentage of participants with PUCAI remission at Week 12 will be

		participants have a need for alternative treatments.<br><br>Exclusion Criteria: Aimed at ensuring participant safety by excluding those with conditions that could confound efficacy assessments or pose a risk with the intervention. Excluding participants with recent investigational product exposure ensures clear assessment of guselkumab's effects.<br><br>Sex/Gender and Ages: Including all genders and a wide age range of pediatric participants ensures the findings are applicable to the broader pediatric population with ulcerative colitis.<br><br>Arms and Interventions: A placebo comparator is essential for assessing the true efficacy and safety of guselkumab, while the choice of administration routes allows evaluation of the most effective and patient-friendly method.<br><br>Primary and Secondary Outcome Measures: These measures are chosen to comprehensively assess the efficacy of guselkumab in inducing clinical remission, improving endoscopic outcomes, and enhancing quality of life, which are critical for evaluating the overall benefit in pediatric ulcerative colitis management	reported. It comprises 6 scales and ranges between 0 and 85 points. The scales are abdominal pain, rectal bleeding, stool consistency, number of stools, nocturnal bowel movement, and activity level. The PUCAI score is calculated as the sum of the 6 subscores. A PUCAI score of less than (<) 10 indicates remission.<br>Percentage of Participants with Symptomatic Remission at Week 12 [Time Frame: Week 12]<br>Percentage of participants with symptomatic remission at Week 12 will be reported. Symptomatic remission is defined as a stool frequency subscore of 0 or 1 and a rectal bleeding subscore of 0, where the stool frequency subscore has not increased from induction baseline.<br>United States: Percentage of Participants with Endoscopic Improvement at Week 12 [Time Frame: Week 12]<br>Percentage of participants with endoscopic improvement as assessed by Mayo endoscopy subscore at Week 12 will be reported. Endoscopic improvement is defined as the Mayo endoscopy subscore of 0 or 1 with no friability present on the endoscopy.<br>European Union: Percentage of Participants with Endoscopic Healing at Week 12 [Time Frame: Week 12]<br>Percentage of participants with endoscopic healing as assessed by Mayo endoscopy subscore at Week 12 will be reported. Endoscopic healing is defined as the Mayo endoscopy subscore of 0 or 1 with no friability present on the endoscopy.<br>Percentage of Participants with Clinical Response at Week 12 [Time Frame: Week 12]<br>Percentage of participants with clinical response as assessed by modified Mayo score at Week 12 will be reported. Modified Mayo score is a 3-component (stool frequency, rectal bleeding, and endoscopy subscores) assessment and does not include the physician's global assessment. A decrease from baseline in the modified Mayo score

			by greater than or equal to (>=) 30 percent and >= 2 points, with either a decrease from baseline in the rectal bleeding subscore of >= 1 or a rectal bleeding subscore of 0 or 1.<br>Percentage of Participants with Symptomatic Remission at Week 56 [Time Frame: Week 56]<br>Percentage of participants with symptomatic remission at Week 56 will be reported. Symptomatic remission is defined as a stool frequency subscore of 0 or 1 and a rectal bleeding subscore of 0, where the stool frequency subscore has not increased from induction baseline.<br>United States: Percentage of Participants With Endoscopic Improvement at Week 56 [Time Frame: Week 56]<br>Percentage of participants with endoscopic improvement as assessed by Mayo endoscopy subscore at Week 56 will be reported. Endoscopic improvement is defined as the Mayo endoscopy subscore of 0 or 1 with no friability present on the endoscopy.<br>European Union: Percentage of Participants With Endoscopic Healing at Week 56 [Time Frame: Week 56]<br>Percentage of participants with endoscopic healing as assessed by Mayo endoscopy subscore at Week 56 will be reported. Endoscopic healing is defined as the Mayo endoscopy subscore of 0 or 1 with no friability present on the endoscopy.<br>Percentage of Participants with Corticosteroid-free Clinical Remission at Week 56 [Time Frame: Week 56]<br>Percentage of participants with corticosteroid-free clinical remission at Week 56 will be reported. Corticosteroid free clinical remission is defined as a Mayo stool frequency subscore of 0 or 1, a rectal bleeding subscore of 0, and an endoscopy subscore of 0 or 1 with no friability present on the endoscopy, where the stool frequency subscore has not increased from induction baseline (Week 0), and not receiving corticosteroids for at least 8 weeks prior to Week 56

			Percentage of Participants with Clinical Response at Week 56 [Time Frame: Week 56]<br>Percentage of participants with clinical response as assessed by modified Mayo score at Week 56 will be reported. Modified Mayo score is a 3-component (stool frequency, rectal bleeding, and endoscopy subscores) assessment and does not include the physician's global assessment. A decrease from baseline in the modified Mayo score by >= 30 percent and >= 2 points, with either a decrease from baseline in the rectal bleeding subscore of >= 1 or a rectal bleeding subscore of 0 or 1.<br>Percentage of Participants Histo-endoscopic Mucosal Improvement at Week 56 [Time Frame: Week 56]<br>Percentage of participants histo-endoscopic mucosal healing per endoscopy subscore and histologic improvement at Week 56 will be reported. Histologic-endoscopic mucosal healing is defined as achieving a combination of histologic improvement and endoscopic improvement (US) or endoscopic healing (EU) (endoscopy subscore of 0 or 1).<br>Percentage of Participants with Symptomatic Remission at Week 56 Among Participants who had Symptomatic Remission at Week 12 [Time Frame: Week 12, Week 56]<br>Percentage of participants with symptomatic remission at Week 56 among participants who had symptomatic remission at Week 12 will be reported. Symptomatic remission score is defined as a stool frequency subscore of 0 or 1 and a rectal bleeding subscore of 0, where the stool frequency subscore has not increased from induction baseline.<br>Percentage of Participants Who Achieve Endoscopic Normalization at Week 56 [Time Frame: Week 56]<br>Percentage of participants who achieve endoscopic normalization with an endoscopy subscore of 0 at Week 56 will be reported.

			Percentage of Participants With PUCAI Remission at Week 56 [Time Frame: Week 56]<br>Percentage of participants with PUCAI remission at Week 56 will be reported. PUCAI comprises of 6 scales and ranges between 0 and 85 points. The scales are: abdominal pain, rectal bleeding, stool consistency, number of stools, nocturnal bowel movement, and activity level. The PUCAI score is calculated as the sum of the 6 subscores. A PUCAI score of less than (<) 10 indicates remission.<br>Serum Concentration of Guselkumab During Induction Phase [Time Frame: From Week 0 to Week 12]<br>Serum samples will be analyzed to determine concentrations of guselkumab overtime.<br>Serum Concentration of Guselkumab During Maintenance Phase [Time Frame: From Week 12 to Week 56]<br>Serum samples will be analyzed to determine concentrations of guselkumab over time.<br>Number of Participants with Incidence of Anti-guselkumab Antibodies [Time Frame: Up to Week 68]<br>Number of participants with anti-guselkumab antibodies for all study treatment regimens will be assessed.<br>Percentage of Participants with Adverse Events (AEs) [Time Frame: Up to Week 68]<br>Percentage of participants with AEs will be reported. An AE is any untoward medical occurrence in a clinical study participant administered a pharmaceutical (investigational or non-investigational) product. An AE does not necessarily have a causal relationship with the intervention.<br>Percentage of Participants with Serious Adverse Events (SAEs) [Time Frame: Up to Week 68]<br>Percentage of participants with SAEs will be reported. An SAE is an AE resulting in any of the following outcomes or deemed significant for any other reason: death; initial or prolonged inpatient hospitalization; life-threatening experience

			(immediate risk of dying); persistent or significant disability or incapacity; congenital anomaly.<br>Percentage of Participants with AEs Leading to Discontinuation of Study Intervention [Time Frame: Up to Week 68]<br>Percentage of participants with AEs leading to discontinuation of study intervention will be reported.
17	Initial Trial Description<br>Official Title: A Randomized, Double-blind, Placebo-controlled Clinical Study to Evaluate Mavacamten in Adolescents (Age 12 Years to < 18 Years) With Symptomatic Obstructive Hypertrophic Cardiomyopathy<br><br>Brief Summary: The purpose of this study is to evaluate the efficacy, safety, and pharmacokinetics of mavacamten in adolescent patients with symptomatic obstructive hypertrophic cardiomyopathy (HCM).<br><br>Study Type: Interventional<br><br>Study Phase: Phase 3<br><br>Study Design: Allocation: Randomized<br>Interventional Model: Parallel Assignment<br>Masking: Quadruple (Participant Care Provider Investigator Outcomes Assessor)<br>Primary Purpose: Treatment	Inclusion Criteria:<br>Adolescents aged 12 years to <18 years at the time of consent.<br>Clinically diagnosed with symptomatic obstructive hypertrophic cardiomyopathy (HCM), confirmed by echocardiography (left ventricular outflow tract (LVOT) gradient ≥30 mmHg at rest or with provocation).<br>New York Heart Association (NYHA) functional class II or III.<br>Stable medical regimen for at least 4 weeks prior to randomization.<br>Ability to provide informed assent (and consent from a parent or legal guardian).<br><br>Exclusion Criteria:<br>Previous heart transplant or listed for heart transplantation.<br>Major cardiovascular event (e.g., myocardial infarction, stroke) within the last 6 months.<br>Any planned major surgery, including septal reduction therapy, within the next 6 months.<br>Concomitant use of other investigational drugs or treatments for HCM.<br>Known hypersensitivity to mavacamten or any component of the formulation.	Inclusion Criteria:<br><br>Diagnosis of HCM<br>Presence of LVOT obstruction<br>Presence of symptoms<br><br>Exclusion Criteria:<br><br>Phenocopy diseases resulting in myocardial hypertrophy not related to sarcomere dysfunction<br>Evidence of LVEF <50% in prior 6 months<br>Planned escalation in HCM therapy or upcoming intervention (eg, major cardiac surgery, HCM medication dose increase)<br><br>Sex/Gender: All<br><br>Ages: 12 Years to 17 Years (Child)<br><br>Arms and Interventions:<br>Experimental: Mavacamten<br>Participants assigned to this arm will receive mavacamten from day 1 to end of treatment at week 56.<br>Interventions:<br>Drug: Mavacamten<br>Experimental: Placebo<br>Participants assigned to this arm will receive mavacamten from week 28 to end of treatment at week 56.<br>Interventions:<br>Drug: Placebo<br><br>Primary Outcome Measures:<br>Change from baseline in Valsalva left ventricular outflow tract (LVOT) (VLVOT) gradient [Time Frame: At Week 28]<br><br>Secondary Outcome Measures:

	Conditions: Cardiomyopathy, Hypertrophic<br><br>Intervention / Treatment:<br>Drug: Mavacamten. Specified dose on specified days<br>Other Names: BMS-986427<br>Drug: Placebo. Specified dose on specified days	Pregnant or breastfeeding females.<br><br>Sex/Gender: ALL<br><br>Ages: 12 years to <18 years<br><br>Arms and Interventions:<br>Participant Group/Arm 1: Mavacamten Treatment<br>Intervention/Treatment: Drug: Mavacamten. Administered orally once daily at a specified dose for 24 weeks.<br><br>Participant Group/Arm 2: Placebo Comparator<br>Intervention/Treatment: Drug: Placebo. Administered orally once daily at a specified dose for 24 weeks.<br><br>Primary Outcome Measures:<br>Outcome Measure: Change in peak oxygen consumption (VO2) from baseline to Week 24.<br>Measure Description: This will assess the improvement in exercise capacity as a result of treatment.<br>Time Frame: Baseline and 24 weeks.<br><br>Secondary Outcome Measures:<br>Outcome Measure: Change in NYHA functional class from baseline to Week 24.<br>Measure Description: This will evaluate the improvement in symptoms and functional status.<br>Time Frame: Baseline and 24 weeks.<br><br>Outcome Measure: Change in LVOT	Change from baseline in resting LVOT gradient [Time Frame: At Week 28]<br>Change from baseline in post-exercise peak LVOT gradient [Time Frame: At Week 28]<br>Change from baseline in maximal wall thickness [Time Frame: At Week 28]<br>Change from baseline in ratio between early mitral inflow velocity and mitral annular early diastolic velocity (E/e') [Time Frame: At Week 28]<br>Proportion of participants achieving an increase from baseline to Week 28 in peak oxygen uptake test (pVO2) [Time Frame: From baseline up to Week 28]<br>Proportion of participants achieving a reduction from baseline to Week 28 in maximal LVOT gradient to < 30 mmHg [Time Frame: From baseline up to Week 28]<br>Proportion of participants with at least 1 class improvement in New York Heart Association (NYHA) class from baseline to Week 28 [Time Frame: From baseline up to Week 28]<br>Proportion of participants with at least 1 grade improvement in mitral regurgitation at Week 28 [Time Frame: From baseline up to Week 28]<br>Number of participants with treatment-emergent adverse events (TEAEs) [Time Frame: Up to Week 74]<br>Number of participants with treatment-emergent serious adverse events (TESAEs) [Time Frame: Up to Week 74]<br>Change from baseline in electrocardiogram (ECG) (QT interval) [Time Frame: At Week 28]<br>Number of participants with left ventricular ejection fraction (LVEF) ≤ 30% [Time Frame: Up to Week 56]<br>Number of participants with LVEF ≤ 50% [Time Frame: Up to Week 56]<br>Trough observed plasma concentration (Ctrough) [Time Frame: Up to Week 56]

		gradient from baseline to Week 24.<br>Measure Description: This will assess the reduction in obstruction within the heart.<br>Time Frame: Baseline and 24 weeks.<br><br>Outcome Measure: Safety and tolerability assessed by the incidence of adverse events.<br>Measure Description: This will monitor the safety profile of mavacamten in the adolescent population.<br>Time Frame: Up to 24 weeks.<br><br>Clinical Reasoning:<br><br>Inclusion Criteria: Focused on adolescents with symptomatic obstructive HCM to evaluate the efficacy and safety of mavacamten in this specific population, ensuring participants are in a specific stage of disease severity (NYHA class II or III) for uniformity.<br>Exclusion Criteria: Aimed at minimizing risks by excluding those with recent major cardiovascular events, planned surgeries, or conditions that could confound the study outcomes. Excluding pregnant or breastfeeding females is standard to avoid potential drug effects on the fetus or infant.<br>Sex/Gender and Ages: Including all genders and a specific age range ensures the study is representative of the	Post-dose plasma concentration of mavacamten [Time Frame: Up to Week 56]<br>Maximum observed concentration (Cmax) [Time Frame: Up to Week 56]<br>Area under the concentration-time curve (AUC) [Time Frame: Up to Week 56]<br>Proportion of participants who evaluate taste and swallowability as neutral or better using taste and swallowability scales [Time Frame: At Day 1 and Week 11]<br>Change from baseline in the Hypertrophic Cardiomyopathy Symptom Questionnaire - Shortness of Breath (HCMSQ SoB) domain [Time Frame: At Week 28]

		adolescent population with HCM, maximizing the applicability of the study findings.<br>Arms and Interventions: A placebo-controlled design allows for a clear evaluation of mavacamten's efficacy and safety, with parallel assignment ensuring equal and unbiased distribution of participants.<br>Primary and Secondary Outcome Measures: Chosen to comprehensively evaluate the impact of mavacamten on exercise capacity, symptom severity, physiological obstruction, and safety within the adolescent population, providing a holistic view of its potential benefits and risks.	
18	Initial Trial Description<br>Official Title: A Phase III, Multicentre, Randomized, Double-blind, Single-Dose, 2-Arm, 2-Period, Crossover Study to Investigate the Efficacy of PT027 Compared With Placebo on Exercise-Induced Bronchoconstriction in Adult Patients With Asthma (BREATH)<br><br>Brief Summary: The purpose of this Phase III, multicentre, randomized, double-blind, single-dose, 2-period, crossover study is to assess the efficacy and safety of PT027	Inclusion Criteria:<br>Diagnosis of Asthma: Participants must have a physician-diagnosed history of asthma for at least 6 months prior to enrollment.<br>Age: Participants aged 18 to 65 years. This age range is chosen to focus on adults with a broad range of physical capabilities and to exclude pediatric and older populations who may have different physiological responses or increased risk of adverse events.<br>Exercise-Induced Bronchoconstriction (EIB): Participants must have a documented history of exercise-induced bronchoconstriction or	Inclusion Criteria:<br><br>Female or male aged 18 to 70 years at the time of informed consent.<br>Documented history of asthma for at least 6 months prior to Visit 1<br>Receiving 1 of the following asthma therapies with stable dosing for at least the 4 weeks before Visit 1 (no other asthma therapies are permitted during the study):<br><br>Short-acting β 2-adrenoreceptor agonist (SABA) used as needed;<br>Low- to medium-dose maintenance therapy with inhaled corticosteroid (ICS) and SABA used as needed.<br>Demonstrate acceptable MDI administration technique (use of a spacer device during the treatment phase is not permitted)<br><br>Exclusion Criteria:<br><br>Chronic obstructive pulmonary disease or other significant lung

	(budesonide/albuterol sulfate) metered-dose inhaler compared with placebo on exercise-induced bronchoconstriction in adult patients with asthma. Subjects will receive each study treatment on separate visits and undergo a treadmill exercise challenge test so that the effect of study treatment on exercise-induced bronchoconstriction can be evaluated<br><br>Study Type: Interventional<br><br>Study Phase: Phase 3<br><br>Study Design:<br>Allocation: Randomized<br>Interventional Model: Crossover Assignment<br>Masking: Quadruple (Participant Care Provider Investigator Outcomes Assessor)<br>Primary Purpose: Treatment<br><br>Conditions: Asthma, Exercise-Induced<br><br>Intervention / Treatment:<br>Drug: Budesonide/albuterol metered-dose inhaler 160/180 µg<br>Budesonide/albuterol combination aerosol for inhalation, single dose (given as 2 actuations of 80/90 µg)<br>Other Names:	demonstrate EIB during a screening treadmill exercise challenge test, defined as a decrease in FEV1 (Forced Expiratory Volume in 1 second) of ≥10% from baseline.<br>Stable Asthma: Participants must have stable asthma, defined as no asthma exacerbations requiring systemic corticosteroids or hospitalization within the last 3 months.<br><br>Exclusion Criteria:<br>Other Respiratory Diseases: Participants with other significant respiratory diseases (e.g., COPD, cystic fibrosis) that could interfere with the study outcomes.<br>Recent Respiratory Infections: Participants who have had a respiratory tract infection within 4 weeks prior to the study start.<br>Pregnancy or Breastfeeding: Women who are pregnant, planning to become pregnant, or breastfeeding, due to unknown effects of the drug on fetal and infant health.<br>Severe Asthma: Participants with severe asthma as defined by the requirement for oral corticosteroids or high-dose inhaled corticosteroids.<br>Allergy to Study Medications: Participants with known hypersensitivity to budesonide, albuterol, or any component of the inhaler formulation.	disease (eg, chronic bronchitis, emphysema, bronchiectasis with the need of treatment, cystic fibrosis, bronchopulmonary dysplasia), including regular or occasional use of oxygen.<br>Systemic corticosteroids (SCS) use (any dose and any indication) within 3 months before Visit 1.<br>History of life-threatening asthma, defined by past intubations for asthma, or intensive care unit admission for asthma within the prior 24 months.<br>Receiving regular maintenance treatment with prohibited anti-inflammatory or long-acting bronchodilator asthma medication (inhaled, nebulized, oral, or systemic) within 1 month prior to Visit 1.<br>Unable to tolerate the lung function testing performed after exercise challenge test without use of rescue medication.<br>Current smokers, former smokers with >10 pack-years history, or former smokers who stopped smoking <6 months before Visit 1 (including all forms of tobacco, e-cigarettes [vaping], and marijuana).<br>Completed treatment for lower respiratory infection within 6 weeks prior to Visit 1, regardless if resulting in accompanying asthma symptoms aggravation or not.<br>Upper respiratory infection involving antibiotic treatment not resolved within 7 days prior to Visit 1.<br>Received any marketed (eg, omalizumab, mepolizumab, reslizumab, benralizumab, dupilumab) or investigational biologic within 3 months before Visit 1, or any other prohibited medication.<br>Historical or current evidence of a clinically significant disease.<br>History of psychiatric disease or intellectual deficiency.<br>Having a scheduled or planned hospitalization during the study.<br>Inability (and/or unwillingness) to abstain from protocol-defined prohibited medications during the study.

	PT027 (BDA MDI) 160/180 µg<br>Drug: Placebo metered-dose inhale. Placebo aerosol for inhalation, single dose (given as 2 actuations)<br>Other Names: Placebo MDI	Sex/Gender:<br>ALL: The study will include both male and female participants to ensure the findings are applicable across genders.<br><br>Ages:<br>18 to 65 years: This age range includes a broad adult population while minimizing risks associated with pediatric and older populations.<br><br>Arms and Interventions:<br>Active Treatment Arm: Participants will receive a single dose of PT027 (budesonide/albuterol metered-dose inhaler 160/180 µg).<br>Placebo Arm: Participants will receive a single dose of a placebo metered-dose inhaler.<br><br>Primary Outcome Measures:<br>Change in FEV1 from baseline following exercise challenge test. This will assess the efficacy of PT027 in preventing exercise-induced bronchoconstriction.<br>Measure Description: Percentage change in FEV1 from baseline to post-exercise challenge at each visit. Time Frame: Baseline and 30 minutes post-exercise challenge.<br><br>Secondary Outcome Measures:<br>Time to onset of EIB: Time from the start of the exercise challenge to the onset of a 10% reduction in FEV1 from	Use of any herbal products by inhalation or nebulizer within 2 weeks of Visit 1 and/or the unwillingness to stop during the study duration.<br>Significant abuse of alcohol or drugs.<br><br>Sex/Gender: All<br><br>Ages: 18 Years to 70 Years (Adult, Older Adult)<br><br>Arms and Interventions:<br>Experimental: A/B - Treatment with PT027 (BUDESONIDE/ALBUTEROL) 160/180 µg followed by treatment with Placebo<br>Subjects randomized to receive a single dose of PT027 160/180 µg in treatment Period 1, and a single dose of Placebo in treatment Period 2.<br>Interventions:<br>Drug: Budesonide/albuterol metered-dose inhaler 160/180 µg<br>Drug: Placebo metered-dose inhaler<br>Experimental: B/A - Treatment with Placebo followed by treatment with PT027 (BUDESONIDE/ALBUTEROL) 160/180 µg<br>Subjects randomized to receive a single dose of Placebo in treatment Period 1, and a single dose of PT027 160/180 in treatment Period 2.<br>Interventions:<br>Drug: Budesonide/albuterol metered-dose inhaler 160/180 µg<br>Drug: Placebo metered-dose inhaler<br><br>Primary Outcome Measures:<br>Maximum Percentage Fall From Post-dose, Pre-exercise Baseline in Forced Expiratory Volume in 1 Second (FEV_1) Observed up to 60 Minutes Post-exercise Challenge [Time Frame: Up to 60 minutes post-exercise challenge]<br>Lung function was measured by spirometry. Spirometry assessments were completed 5 minutes before dosing, 30 minutes after dosing (baseline; 5 minutes before the exercise challenge), and then 5, 10, 15, 30, and 60 minutes after the exercise challenge. A reduction in

		the post-dose baseline. Time Frame: During exercise challenge test.<br>Duration of EIB: Duration for which the FEV1 remains ≥10% below the post-dose baseline following the exercise challenge. Time Frame: During exercise challenge test.<br>Participant-reported asthma symptoms: Assessed using a standardized asthma symptom questionnaire. Time Frame: Pre-dose, immediately post-exercise, and 30 minutes post-exercise.<br><br>Clinical Reasoning:<br>Inclusion and Exclusion Criteria: These criteria are designed to select a homogenous group of adult patients with asthma who are specifically affected by exercise-induced bronchoconstriction, while excluding those with conditions or situations that could confound the study results or pose additional risks.<br>Sex/Gender and Age: Including all genders and a broad range of adult ages ensures the results are generalizable to the adult asthma population while minimizing risks to vulnerable groups.<br>Arms and Interventions: The crossover design allows each participant to serve as their own control, increasing the study's power to detect differences between the active treatment and placebo.	FEV_1 was expected due to the effects of asthma and exercise on breathing and lung function. The percentage fall in FEV_1 was calculated based on the baseline value and maximum percentage fall value during the 60-minute assessment period.<br><br>Secondary Outcome Measures:<br>Percentage of Subjects With a Maximum Percentage Fall in FEV_1 Post-exercise Challenge of <10% and <20% [Time Frame: Up to 60 minutes post exercise challenge]<br>The percentage fall in FEV_1 was calculated based on the baseline value and maximum percentage fall value during the 60-minute assessment period, and the percentage of subjects with a maximum percentage fall <10% and <20% was determined<br>Percentage Fall From Post-dose, Pre-exercise Baseline in FEV1 at Each Time Point Within 60 Minutes Post-exercise Challenge [Time Frame: Up to 60 minutes post exercise challenge]<br>The percentage fall in FEV_1 at each time point was calculated based on the baseline value and percentage fall value during the 60-minute assessment period at each time point.<br>Post-Exercise FEV1 Area Under the Curve from 0 to 30 Minutes (AUC0-30min) [Time Frame: Up to 30 minutes post exercise challenge]<br>FEV1 AUC0-30min will be derived for the changes from the post-dose, pre-exercise baseline using the trapezoidal rule and will be normalized by dividing by the actual time (in minutes) from dosing to the last included measurement, scheduled at 30 minutes post-exercise challenge.<br>Time To Recovery, Defined As The Time From Completion Of The Exercise Challenge To The First Measured Post-exercise Challenge FEV1 Value Within 10% Of The Post-dose, Pre-exercise Challenge

		Primary and Secondary Outcome Measures: These measures are chosen to comprehensively evaluate the efficacy of PT027 in preventing and managing exercise-induced bronchoconstriction, from physiological measures (FEV1) to patient-reported outcomes (symptoms), providing a holistic view of the treatment's impact.	Baseline FEV1 [Time Frame: Up to 60 minutes post exercise challenge] Time to recovery will be derived as the time (minutes) post-exercise challenge in which the FEV1 result returns to within 10% of the value recorded at the post-dose, pre-exercise baseline.
19	Initial Trial Description<br>Official Title: Effectiveness & Safety of Perfenidone in Type 2 Diabetic Patients With Diabetic Neuropathy: A Randomized Controlled Trial<br><br>Brief Summary: The purpose of this study is to determine the effectiveness & safety of pirfenidone in type 2 diabetic patients with diabetic nephropathy<br><br>Study Type: Interventional<br><br>Study Phase: Phase 4<br><br>Study Design:<br>Allocation: Randomized<br>Interventional Model: Parallel Assignment<br>Masking: Single (Participant)<br>Primary Purpose: Treatment<br><br>Conditions:	Inclusion Criteria:<br>Diagnosis of type 2 diabetes mellitus (T2DM) based on ADA criteria.<br>Clinical diagnosis of diabetic nephropathy, confirmed by albuminuria (albumin-to-creatinine ratio [ACR] > 30 mg/g) and/or reduced eGFR (30-60 mL/min/1.73 m^2) in the context of T2DM.<br>Age 18 years or older.<br>Stable glycemic control (HbA1c ≤ 8.5%) on current regimen for at least 3 months prior to enrollment.<br>Ability to provide informed consent.<br><br>Exclusion Criteria:<br>Type 1 diabetes mellitus.<br>Advanced chronic kidney disease (CKD) (eGFR < 30 mL/min/1.73 m^2).<br>History of hypersensitivity or adverse reaction to pirfenidone.<br>Current or recent (within 3 months) use of immunosuppressive therapy.	Inclusion Criteria:<br><br>Diabetic Nephropathy Stage 3 (eGFR 30-59 mL/min/1.73m2) Type 2 Diabetes HbA1c <7.5% Patient taking ACEi/ARB for at least 3 months BP <140/90<br><br>Exclusion Criteria:<br><br>History of photosensitivity rash History of decompensated liver or cardiac disease History of urinary tract infection Pregnancy or lactation History of nephrotoxic drugs or hakeem medication Polycystic kidney disease History of autoimmune disease History of hypersensitivity to study drugs<br><br>Sex/Gender: All<br><br>Ages: 30 Years to 65 Years (Adult, Older Adult)<br><br>Arms and Interventions:<br>Experimental: Group A (Pirfenidone) Capsule Pirfenidone 200mg, 2 X 8hrly for 3 months<br>Interventions:<br>Drug: Pirfenidone<br>Placebo Comparator: Group B Placebo capsules - 2 X 8hrly for 3 months<br>Interventions:<br>Other: Placebo<br><br>Primary Outcome Measures:

	Diabetic Nephropathies<br>Type 2 Diabetes<br><br>Intervention / Treatment:<br>Drug: Pirfenidone. Capsule Pirfenidone 1200mg/ day in divided doses for 3 months<br>Other: Placebo 2 Capsules 3 times a day for 3 months	Pregnant or breastfeeding women.<br>Active liver disease or hepatic dysfunction (ALT or AST > 2.5 times the upper limit of normal).<br>Participation in another clinical trial involving investigational products within the last 30 days.<br><br>Sex/Gender:<br>All (both male and female participants are eligible).<br><br>Ages:<br>18 years and older.<br><br>Arms and Interventions:<br>Experimental Group/Arm: Participants will receive Pirfenidone 1200mg per day in divided doses for 3 months.<br>Control Group/Arm: Participants will receive a placebo (2 capsules 3 times a day) for 3 months.<br><br>Primary Outcome Measures:<br>Change in eGFR from baseline to 3 months. This will assess the impact of pirfenidone on kidney function in participants with diabetic nephropathy.<br>Change in Albuminuria (ACR) from baseline to 3 months. This will evaluate the effect of pirfenidone on urinary albumin excretion, a marker of nephropathy progression.<br><br>Secondary Outcome Measures:<br>Change in HbA1c levels from baseline to 3 months. Although not	15% improvement in eGFR [Time Frame: 6 months]<br>15% improvement in eGFR<br><br>Secondary Outcome Measures:<br>Safety - Adverse Effects of Drugs [Time Frame: 6 months]<br>Adverse Effects of the drugs

		the primary focus, it's essential to monitor glycemic control throughout the study.<br>Incidence of Adverse Events throughout the study duration. This will assess the safety and tolerability of pirfenidone in this population.<br>Quality of Life Assessment using a validated scale (e.g., SF-36) at baseline and 3 months. This will evaluate the impact of treatment on participants' quality of life.<br><br>Clinical Reasoning:<br>Inclusion Criteria are designed to select a population with T2DM and moderate diabetic nephropathy, who may benefit from pirfenidone treatment. Stable glycemic control is required to minimize confounding effects on nephropathy progression.<br>Exclusion Criteria aim to ensure participant safety by excluding those with conditions that could be adversely affected by pirfenidone or confound its effects on diabetic nephropathy.<br>Sex/Gender and Age criteria allow for a broad representation of the adult T2DM population with nephropathy, enhancing the generalizability of the findings.<br>Arms and Interventions are structured to compare the effectiveness and safety of pirfenidone against a placebo, following a	

		standard parallel assignment model.<br>Primary Outcome Measures directly assess the impact of pirfenidone on markers of nephropathy progression, which is the primary goal of the study.<br>Secondary Outcome Measures provide additional safety information, assess the broader impact of treatment on glycemic control and quality of life, offering a comprehensive view of pirfenidone's potential benefits and risks in this population.	
20	Initial Trial Description<br>Official Title: A Phase 3, Randomized, Placebo-controlled, Double-blind Study to Investigate the Long Term Safety of Fezolinetant in Japanese Women Suffering From Vasomotor Symptoms (Hot Flashes) Associated With Menopause	Inclusion Criteria:<br>Female participants of Japanese descent.<br>Ages 40-65 years, experiencing natural menopause or surgical menopause (bilateral oophorectomy).<br>Reporting moderate to severe vasomotor symptoms (hot flashes) associated with menopause, defined as experiencing at least 7 moderate to severe hot flashes per day or at	Inclusion Criteria:<br><br>Participant confirmed as menopausal per one of the following criteria at the screening visit (visit 1):<br><br>For a post-menopausal participant: Spontaneous amenorrhea for >/=12 consecutive months; Spontaneous amenorrhea for >/=6 months with biochemical criteria of menopause (follicle-stimulating hormone (FSH) > 40 IU/L); Having had bilateral oophorectomy >/=6 weeks prior to the screening visit (visit 1) (with or without hysterectomy); Having had hysterectomy without bilateral

	Brief Summary: Hot flashes are the most common reason women going through menopause seek medical attention. Hormone replacement therapy, or HRT, is most often prescribed to treat hot flashes. However, HRT can't be used by all women or for as long as may be needed. Researchers want to find other ways to treat hot flashes. Fezolinetant is a medicine to treat hot flashes in women going through menopause. Fezolinetant is an approved medicine in the US. Further studies are needed before it is available in other regions such as Asia. In this study fezolinetant will be used to treat hot flashes in Japanese women going through menopause. This study will confirm the safety of fezolinetant and how well the women tolerate the treatment. Women will either take fezolinetant or a placebo. This is decided by chance alone. The placebo looks like fezolinetant but will not have any medicine in it. The women will take 1 tablet of the study medicine (fezolinetant or the placebo) once a day for up to 52 weeks. During the study, the women will visit their study clinic	least 50 per week at baseline.<br>Willing and able to comply with all study procedures and available for the duration of the study.<br>Able to give informed consent.<br><br>Exclusion Criteria:<br>History of breast cancer or other hormone-sensitive cancers.<br>Current or recent (within the last 6 months) use of hormone replacement therapy or other medications known to affect vasomotor symptoms.<br>Uncontrolled hypertension (BP >160/100 mmHg).<br>Significant liver disease or renal impairment.<br>History of thromboembolic events or conditions predisposing to thrombosis.<br>Presence of any psychiatric or cognitive condition that, in the opinion of the investigator, would interfere with the participant's ability to comply with the study protocol.<br><br>Sex/Gender: Female<br><br>Ages: 40-65 years<br><br>Arms and Interventions:<br>Participant Group/Arm 1 - Fezolinetant: Participants in this arm will receive Fezolinetant, orally, once daily for 52 weeks.<br>Participant Group/Arm 2 - Placebo: Participants in this arm will receive a	oophorectomy with the biochemical criteria of menopause (FSH > 40 IU/L); or Having been confirmed to be post-menopausal in the 2693-CL-0310 study.<br>For a peri-menopausal participant: Spontaneous amenorrhea for >/=60 days but < 6 consecutive months 2 times in the 2 most recent menstrual cycles with biochemical criteria of peri-menopause (FSH > 25 IU/L); or Spontaneous amenorrhea for >/=6 months but < 12 consecutive months with biochemical criteria of peri-menopause (FSH > 25 IU/L and ≤ 40 IU/L); Having had hysterectomy without bilateral oophorectomy with the biochemical criteria of menopause (FSH > 25 IU/L and ≤ 40 IU/L).<br>Participant is seeking treatment for relief of vasomotor symptoms (VMS) associated with menopause.<br>Female participant:<br><br>Is not pregnant and at least 1 of the following conditions apply: Not a women of childbearing potential (WOCBP); WOCBP who has a negative urine pregnancy test day 1 (visit 2) and agrees to follow the contraceptive guidance from the time of informed consent through at least 21 days after final study intervention administration.<br>Must not be breastfeeding or lactating starting at screening and throughout the investigational period and for 21 days after final study intervention administration.<br>Must not donate ova starting at first administration of study intervention and throughout the investigational period and for 21 days after final study intervention administration<br>Participant agrees not to participate in another interventional study while participating in the present study.<br><br>Exclusion Criteria:<br><br>Participant has a history of an undiagnosed uterine bleeding within the 6 months prior to the screening visit (visit 1).

	for a check-up about every 4 weeks for up to 52 weeks (1 year). At each visit they will be asked if they had any medical problems. Other checks will include a medical examination and vital signs (temperature, blood pressure and pulse). At some visits, the women will have an ECG to check their heart rhythm and some blood and urine samples will be taken for laboratory tests. During a couple of visits, women who have a womb (uterus) will also have a test called a transvaginal ultrasound. A probe is gently placed inside the vagina. Sound waves will create a picture of the organs in the pelvis. This will allow the study doctor to look more closely at the uterus and surrounding organs. The last clinic visit will be 3 weeks after the women take their final tablet of the study medicine (fezolinetant or the placebo).<br><br>Study Type: Interventional<br><br>Study Phase: Phase 3<br><br>Study Design:<br>Allocation: Randomized<br>Interventional Model: Parallel Assignment<br>Masking: Triple (Participant	placebo, orally, once daily for 52 weeks.<br><br>Primary Outcome Measures:<br>Outcome Measure: Change in the frequency of moderate to severe hot flashes from baseline to Week 12.<br>Measure Description: The average number of moderate to severe hot flashes per day will be recorded by participants in a diary. The change from baseline to Week 12 will be calculated.<br>Time Frame: Baseline and Week 12.<br><br>Secondary Outcome Measures:<br>Outcome Measure: Change in the severity of hot flashes from baseline to Week 12.<br>Measure Description: Participants will rate the severity of each hot flash in their diary. The change in average severity from baseline to Week 12 will be calculated.<br>Time Frame: Baseline and Week 12.<br>Outcome Measure: Quality of Life assessment using the Menopause-Specific Quality of Life Questionnaire (MENQOL).<br>Measure Description: Change in MENQOL scores from baseline to Week 52.<br>Time Frame: Baseline and Week 52.<br>Outcome Measure: Safety and tolerability assessed by the incidence of adverse events.	Participant has a current malignant tumor or history (except for a participant who has not received treatment for malignant tumors for at least 5 years before informed consent acquisition and was not considered to have recurrence) of a malignant tumor except for non-metastatic basal cell carcinoma of the skin.<br>Participant has a medical condition or chronic disease (including history of neurological [including cognitive], hepatic, renal, cardiovascular, gastrointestinal, pulmonary [e.g., moderate asthma], endocrine, or gynecological disease) that could confound interpretation of the study outcome.<br>Participant uses a prohibited therapy (hormone therapy, hormone replacement therapy (HRT), hormonal contraceptive, any treatment for VMS [prescription medications, over-the-counter, or herbal/Kampo medicines] or strong or moderate cytochrome P450 1A2 (CYP1A2) inhibitors and is not willing to wash out or discontinue use of such drugs from screening visit (visit 1) through the follow-up visit (visit 16) or it is not medically appropriate to discontinue such drugs for the duration of the study.<br>Participant has been randomized/registered in a clinical trial with fezolinetant previously or had previous exposure to marketed fezolinetant elsewhere.<br>Participant has a present or previous history of participation in this study.<br>Participant has received any investigational therapy within 28 days or 5 half-lives, whichever is longer, prior to screening visit (visit 1).<br>Participant has an unacceptable result from the transvaginal ultrasound (TVU) assessment at screening (i.e., full length of endometrial cavity cannot be visualized or presence of clinically significant abnormal findings).<br>Participant has documentation of a clinically significant abnormal Papanicolaou (Pap) test (or

	Investigator Outcomes Assessor)<br>Primary Purpose: Treatment<br><br>Conditions:<br>Hot Flashes<br><br>Intervention / Treatment:<br>Drug: Fezolinetant oral<br>Other Names:<br>ESN364;<br>VEOZAH™<br>Drug: Placebo oral	Measure Description: The number and severity of adverse events will be recorded throughout the study.<br>Time Frame: Up to 55 weeks (52 weeks of treatment plus 3 weeks follow-up).<br><br>Clinical Reasoning:<br><br>Inclusion/Exclusion Criteria: The criteria are designed to select a homogeneous group of postmenopausal women who are experiencing vasomotor symptoms, ensuring that the study population is representative of the target population for Fezolinetant. Excluding women with hormone-sensitive cancers or those on hormone therapy ensures that the effects measured are attributable to Fezolinetant and not confounded by other treatments.<br>Sex/Gender and Ages: The study focuses on women, as menopause and its symptoms are specific to females. The age range is chosen to encompass the typical age range for menopause while ensuring participants are likely to be experiencing menopausal symptoms.<br><br>Arms and Interventions: A placebo-controlled design is essential for assessing the efficacy and safety of Fezolinetant in a blinded manner, reducing bias.<br>Primary and Secondary Outcome Measures: The	equivalent cervical cytology) within 52 weeks prior to the screening visit (visit 1) or at screening.<br>Participant has active liver disease, jaundice, or elevated liver aminotransferases (alanine aminotransferase (ALT) or aspartate aminotransferase (AST)), elevated total bilirubin (TBL) or direct bilirubin (DBL), elevated international normalized ratio (INR), or elevated alkaline phosphatase (ALP) at screening. A participant with mildly elevated ALT or AST up to < 1.5 × upper limit of normal (ULN) can be enrolled if TBL and DBL are normal. Participant with mildly elevated ALP (up to < 1.5 × ULN) can be enrolled if cholestatic liver disease is excluded and no cause other than fatty liver is diagnosed. Participant with Gilbert's syndrome with elevated TBL may be enrolled as long as DBL, hemoglobin and reticulocytes are normal.<br>Participant has creatinine > 1.5 × ULN or estimated glomerular filtration rate using the Modification of Diet in Renal Disease formula </=30 mL/min/1.73 m^2 at screening.<br>Participant has positive hepatitis serology panel (i.e., positive hepatitis B surface (HBs) antigen and/or positive hepatitis C virus (HCV) antibody) at screening. If HCV antibody test result is equivocal, hepatitis C virus ribonucleic acid (HCV RNA) test at study site is allowed. Participant can be enrolled if that result is normal or not abnormal.<br>Participant is not in good general health as determined on the basis of medical history and general physical examination performed at the screening; hematology parameters, biochemistry parameters, pulse rate, blood pressure, electrocardiogram (ECG) outside the reference range for the population studied, or is showing clinically relevant deviations.<br>Participant has a history of suicide attempt or suicidal behavior within 52 weeks prior to study enrollment or suicidal ideation within 52 weeks prior to study enrollment (a response of "yes" to question 4 or 5 on the

		primary outcome directly measures the efficacy of Fezolinetant on the frequency of hot flashes, which is the primary symptom of interest. Secondary outcomes provide additional information on the severity of symptoms, quality of life, and safety, offering a comprehensive understanding of Fezolinetant's effects.	suicidal ideation portion of the Columbia Suicide Severity Rating Scale (C-SSRS)), or is at significant risk to commit suicide at day 1 (visit 2).<br>Participant is unable or unwilling to complete the study procedures.<br>Participant has any condition which makes the participant unsuitable for study participation.<br>Participant has a known or suspected hypersensitivity to fezolinetant or any components of the formulation used.<br>Participant is the investigator or a member of the study site staff.<br>Participant is an employee of Astellas, the study-related contract research organizations (CROs) or site management organizations.<br><br>Sex/Gender: Female<br><br>Ages: 40 Years to 65 Years (Adult, Older Adult)<br><br>Arms and Interventions:<br>Experimental: Fezolinetant<br>Participants will receive fezolinetant once daily for 52 weeks.<br>Interventions:<br>Drug: Fezolinetant<br>Experimental: Placebo<br>Participants will receive matching placebo once daily for 52 weeks.<br>Interventions:<br>Drug: Placebo<br><br>Primary Outcome Measures:<br>Number of participants with Adverse Events (AEs) [Time Frame: Up to Week 55]<br>An AE is any untoward medical occurrence in a patient or clinical study participant, temporally associated with the use of study intervention, whether or not considered related to the study intervention.<br><br>An AE can therefore be any unfavorable and unintended sign (including an abnormal laboratory finding), symptom, or disease (new or exacerbated) temporally associated with the use of study intervention.

			This includes events related to the comparator, if applicable, and events related to the (study) procedures.<br><br>Secondary Outcome Measures:<br>Change from baseline in endometrial thickness in post-menopausal participants [Time Frame: Baseline and up to Week 52]<br>Endometrial thickness is a measure of how thick the lining of the uterus is. Endometrial thickness will be measured by transvaginal ultrasound (TVU).<br>Number of participants with laboratory value abnormalities and/or AEs [Time Frame: Up to Week 55]<br>Number of participants with potentially clinically significant laboratory values.<br>Number of participants with vital sign abnormalities and/or AEs [Time Frame: Up to Week 55]<br>Number of participants with potentially clinically significant vital sign values.<br>Number of participants with electrocardiogram (ECG) abnormalities and/or AEs [Time Frame: Up to Week 52]<br>Number of participants with potentially clinically significant ECG values.<br>Pharmacokinetics (PK) of fezolinetant in plasma: Concentration [Time Frame: Up to Week 52]<br>Concentration will be recorded from the PK plasma samples collected.<br>Pharmacokinetics (PK) of metabolite ES259564 in plasma: Concentration [Time Frame: Up to Week 52]<br>Concentration will be recorded from the PK plasma samples collected.

Table S3: NLP based statistical analytical scoring on LLM-based clinical trial designs

	Overall design	Eligibility criteria	Recruitment	Intervention	Outcome Measures
BLEU	0.04478295	0.04132682	0.25689486	0.07282843	0.0313343
ROUGE-L	0.19870998	0.18931406	0.61798858	0.28428445	0.17543501
METEOR	0.17572606	0.19684976	0.51055223	0.27311108	0.15082139

Table S4: Qualitative scoring by two intendent human clinical experts on defined Likert sales. A. Published RCTs; B. Registered RCTs.

A. Published RCTs:

Reviewer 1

Ground Truth	Safety	Clinical accuracy	Objectivity (Bias)	Pragmatic	Inclusivity and Diversity
1	3	3	3	2	2
2	3	3	3	3	2
3	3	3	3	3	2
4	3	3	3	3	2
5	3	3	3	3	3
6	3	3	2	2	2
7	3	3	3	2	3
8	3	3	3	2	2
9	3	3	2	2	2
10	3	2	3	2	2

LLM	Safety	Clinical accuracy	Objectivity (Bias)	Pragmatic	Inclusivity and Diversity
1	3	3	2	2	2
2	3	2	3	2	3
3	3	3	3	2	2
4	3	3	3	3	2
5	3	3	3	3	2
6	3	3	3	3	2
7	3	2	3	2	1
8	3	3	3	3	3
9	3	3	3	3	2
10	2	2	3	1	2

Reviewer 2

Ground Truth	Safety	Clinical accuracy	Objectivity (Bias)	Pragmatic	Inclusivity and Diversity
1	3	3	3	1	1
2	3	3	3	3	2
3	3	3	3	3	2
4	3	3	3	3	2
5	3	3	3	3	3
6	3	3	2	3	1
7	3	3	3	2	3
8	3	3	3	2	2
9	3	3	2	2	1
10	3	2	3	1	2

LLM	Safety	Clinical accuracy	Objectivity (Bias)	Pragmatic	Inclusivity and Diversity
1	3	3	2	2	2
2	3	2	3	2	3
3	3	3	3	2	2
4	3	3	3	3	2
5	3	3	3	3	2
6	3	3	3	3	2
7	3	2	3	2	1
8	3	3	3	3	3
9	3	3	3	3	2
10	2	2	3	1	2

B. Registered RCTs:

Reviewer 1

Ground Truth	Safety	Clinical accuracy	Objectivity (Bias)	Pragmatic	Inclusivity and Diversity
1	3	3	3	1	1
2	1	1	2	1	2
3	3	3	3	3	3
4	2	3	3	2	2
5	2	2	2	2	2
6	2	3	3	2	3
7	2	2	2	2	3
8	3	3	3	3	3
9	3	3	3	2	3
10	3	3	3	2	2

LLM	Safety	Clinical accuracy	Objectivity (Bias)	Pragmatic	Inclusivity and Diversity
1	3	3	3	2	2
2	2	2	3	3	3
3	3	3	3	3	2
4	2	3	3	2	2
5	2	3	3	2	3
6	3	3	3	2	3
7	2	2	3	2	3
8	3	3	3	2	3
9	3	3	3	2	2
10	2	3	3	2	3

Reviewer 2

Ground Truth	Safety	Clinical accuracy	Objectivity (Bias)	Pragmatic	Inclusivity and Diversity
1	3	3	3	2	1
2	1	1	2	2	2
3	3	3	3	3	3
4	2	2	2	2	2
5	2	2	3	2	2
6	2	3	3	2	3
7	2	2	2	2	3
8	3	3	3	3	3
9	3	3	3	3	3
10	3	3	3	3	3

LLM	Safety	Clinical accuracy	Objectivity (Bias)	Pragmatic	Inclusivity and Diversity
1	2	2	3	3	2
2	2	2	3	3	3
3	3	3	3	3	2
4	2	2	2	2	2
5	1	2	3	2	3
6	3	3	3	2	3
7	2	2	3	2	3
8	3	3	3	2	3
9	3	3	3	3	3
10	2	2	3	2	3
